# One‐Step Assembly of α‐Aryl‐Substituted DOTAs as Superior and Universal Platforms for Multifunctional Theranostics

**DOI:** 10.1002/anie.202519204

**Published:** 2025-10-15

**Authors:** Pak‐Lun Lam, Yue Wu, Lijia Yao, Hei‐Yui Kai, Ho‐Fai Chau, Qian Zhang, Wanqi Zhou, Jean‐Claude G. Bünzli, Ka‐Leung Wong

**Affiliations:** ^1^ Department of Applied Biology and Chemical Technology The Hong Kong Polytechnic University Hung Hom Hong Kong SAR China; ^2^ Department of Chemistry Hong Kong Baptist University 224 Waterloo Rd, Kowloon Tong Kowloon Hong Kong SAR China; ^3^ Institute of Chemical Sciences and Engineering (ISIC) Swiss Federal Institute of Technology (EPFL) Lausanne CH‐1015 Switzerland

**Keywords:** α‐Substituted DOTAs, Bioconjugation, Macrocyclic chelators, Multi‐component reactions (MCRs)

## Abstract

α‐Substituted DOTAs are promising chelators for MRI contrast agents owing to the improved coordination stability and relaxivity of the corresponding Gd(III) complexes. However, their broader application is limited by significant synthetic challenges arising from their multi‐component nature. In this work, we report—for the first time—the use of multi‐component reactions (MCRs) to assemble all necessary building blocks of α‐aryl‐substituted DOTAs in a single step. This strategy yields derivatives with faster coordination kinetics. Furthermore, we extend their application to luminescent lanthanide probes, achieving improved photophysical properties. This MCR approach offers a versatile solution for establishing a library of functionalized diagnostic and therapeutic agents. We are convinced that this work will reshape the field, inspiring broader exploration of α‐aryl‐substituted DOTA derivatives and unlocking their full potential in next‐generation biomedical applications.

## Graphical abstract

A one‐step multi‐component synthetic approach is reported to construct α‐aryl‐substituted DOTAs, which required tedious multi‐step synthesis in all previous works. The synthesized α‐aryl‐substituted DOTAs showed improved performance for developing multifunctional theranostics and represent a universal platform for bioconjugation.

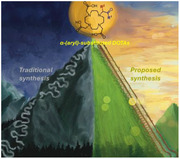

## Introduction

Macrocyclic chelators based on 1,4,7,10‐tetraazacyclododecane‐1,4,7,10‐tetraacetic acid (DOTA) are ideal to form stable complexes with various metal ions for valuable biomedical applications like diagnostic imaging and therapy.^[^ ^1^ ^]^ DOTA‐like chelators are suitable for a variety of radioisotopes, which include positron emitters (e.g., ^64^Cu^2+^, ^68^Ga^3+^) for PET scan, gamma‐emitters (e.g., ^111^In^3+^) for SPECT scan, as well as beta‐emitters (e.g., ^90^Y^3+^, ^161^Tb^3+^, ^177^Lu^3+^) for radiotherapy.^[^ ^2^, ^3^, ^4^, ^5^, ^6^ ^]^ Conjugation of these radioactive complexes to targeting motifs has enabled successful clinical application of the resulting radiopharmaceuticals in the diagnosis and treatment of specific cancers. For instance, based on SSTR‐targeting cyclic peptides and DOTA‐like chelators, a series of radiopharmaceuticals have been approved by the US FDA (e.g., ^68^Ga‐DOTATOC,^[^ ^7^ ^] 177^Lu‐DOTATATE^[^ ^8^ ^]^). In addition to radioisotopes, DOTA‐like chelators are also suitable for gadolinium (III) ion to develop magnetic resonance imaging (MRI) contrast agents. Thanks to the strong paramagnetic effects from seven unpaired electrons of the Gd^3+^ ion, gadolinium‐based contrast agents (GBCAs) became the most used contrast agents for MRI.^[^ ^9^, ^10^ ^]^ Currently, a series of GBCAs based on DOTA‐like chelators have been approved by the US FDA (e.g., Dotarem, Gadovist, and ProHance). Some candidates, like MT218, are undergoing clinical trials, but no targeting GBCAs have been approved.^[^ ^11^ ^]^ Besides, with a suitable organic chromophore, DOTA‐like chelators can be modified to become luminescent lanthanide probes, which provide the advantages of long luminescence lifetimes, enabling time‐resolved imaging techniques that effectively reduce background autofluorescence and improve signal‐to‐noise ratio.^[^ ^12^, ^13^ ^]^ Luminescent lanthanide probes also have sharp emission peaks and large Stokes shifts. This enhances spectral resolution and enables precise multiplexing without spectral overlap. Furthermore, compared to organic dyes, the superior photostability of luminescent lanthanide probes ensures consistent imaging results over extended periods, minimizing photobleaching while maintaining image quality. We have previously reported a few targeting luminescent lanthanide probes,^[^ ^14^, ^15^, ^16^, ^17^, ^18^ ^]^ including work on targeting Cyclin A^[^ ^15^, ^16^ ^]^ and LMP1 of EBV.^[^ ^18^ ^]^

**Figure 1:**
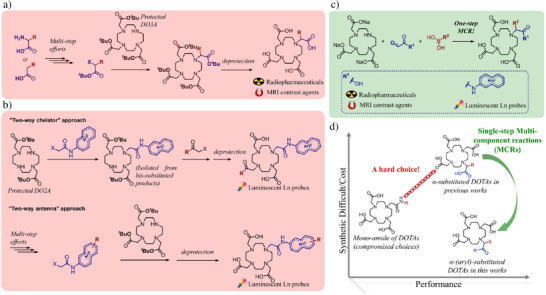
a) Traditional synthetic approaches for α‐substituted DOTAs: multi‐step synthetic efforts were inevitable; b) Traditional synthetic approaches for luminescent lanthanide probes. Three‐component nature of desired products makes them tedious to synthesize. c) This work: A single‐step multi‐component approach for α‐aryl‐substituted DOTAs that is compatible with luminescent lanthanide probes; the tedious synthetic route for (b) and (c) can be bypassed; d) A simple scheme to compare this work with previous works. Previously, a dilemma between synthetic difficulties/cost and performance was raised when choosing a chelator. Although α‐substituted DOTAs show better performances, their synthetic difficulties/costs are significantly higher than the alternative, less performing choice involving DOTA mono‐amides. The strategy developed in this work for α‐aryl‐substituted DOTAs results in synthetic difficulties/cost lower than those for DOTA mono‐amides. In addition, more advantages of α‐aryl‐substituted DOTAs are revealed/developed in this study.

Owing to the increasing demand for targeted diagnosis and therapy, targeting vectors that can specifically localize on the desired biotarget are becoming more popular for conjugation to functional complexes. The most common conjugating site for DOTA‐like chelators is one of their four acetic pendants, giving the mono‐amide of DOTA, like two FDA‐approved radiopharmaceuticals mentioned in the paragraph above. Since the *tris*‐substituted building block *tris*‐*^t^* Bu‐DO3A can be synthesized from 1,4,7,10‐tetraazacyclododecane (cyclen) and *tert*‐butyl haloacetate by an established protocol controllably and on a large scale,^[^ ^19^ ^]^ the targeting motifs or functional groups for conjugation are usually introduced as the substituent of the last nitrogen atom with an *N*‐substituted haloacetamide followed by removing the *tert*‐butyl protecting groups. Although the mono‐amide approach is relatively easy and affordable in synthesis, the resulting products showed decreased coordination stability compared to the conventional DOTA, as one of the acetic pendant groups is converted to amide and contributes less to coordination.^[^ ^20^ ^]^ Previous studies tried to introduce substituents on 1) the backbone of cyclen;^[^ ^21^, ^22^, ^23^, ^24^, ^25^ ^]^ 2) the α‐position of an acetic pendant^[^ ^26^, ^27^, ^28^, ^29^ ^]^ (Figure 1a). Both approaches give potential conjugating site(s) for further bioconjugation while retaining strong coordination ability. The coordination stability and relaxivity of the Gd^3+^ complexes are improved compared with the simple DOTA complex due to the increasing rigidity of the macrocyclic chelators, yet the synthetic difficulties and cost are greatly increased. Synthesizing backbone‐substituted DOTAs requires reconstructing the entire cyclen skeleton by a multistep reaction, while α‐substituted DOTAs have a three‐component nature with a DO3A core (black), an α‐substituted acetic pendant (navy blue), and an α‐substituent (red). α‐Substituted DOTAs are usually synthesized from *tris*‐*^t^* Bu‐DO3A and an α‐halo/α‐tosyl carboxylic acid; the latter has to be prepared from an α‐amino acid or 2‐arylacetic acid by multi‐step synthetic efforts that involve repeated transformation/protection/deprotection of functional groups (Figure 1a).^[^ ^28^, ^30^ ^]^ Therefore, backbone‐substituted DOTA and α‐substituted DOTA are still less studied and applied, although their superiority has been demonstrated for decades. Many FDA‐approved drugs that have undergone clinical trials and other drugs in recent publications still use the mono‐amide approach for conjugating targeting motifs despite the low thermodynamic stability. This may be ascribed to a compromise between performance and synthetic difficulty/cost for both researchers and industries.

For targeting luminescent lanthanide probes, the desired products always consist of three components (Figure 1b): a DOTA‐like chelator (black), an antenna (navy blue), and a targeting vector (or conjugating site for targeting vector, red). Moreover, to ensure good energy transfer efficiency, the antenna is usually deployed close to the lanthanide ion, and the conjugated system of the antenna preferably takes part in coordination. In previous studies, such three‐component products always required tedious multi‐step synthetic tasks to link two of them together, followed by conjugating to the third one. Some studies use two‐way DOTA‐like chelators^[^ ^15^, ^16^ ^]^ to accommodate both antenna and targeting vector, resulting in more tedious chemistries to modify DOTA‐like chelators (usually from a protected DO2A building block), which further sacrifices the coordination ability as one more acetic pendant is converted to amide. Also, the quantum yield & brightness of the corresponding lanthanide complexes may decrease, as these complexes have one net positive charge that is considered disadvantageous for their photophysical properties.^[^ ^31^, ^32^ ^]^ Some previous work functionalized the antennae instead; this also elongates the synthetic route to prepare a more complicated antenna.^[^ ^18^ ^]^ A simple synthetic approach to assemble the three parts together has not yet been reported.

As we identified the synthetic difficulties of both α‐substituted DOTAs and targeted luminescent lanthanide probes arising from their multi‐component nature, we turned to multi‐component reactions (MCRs) to overcome these difficulties. The Petasis reaction is an MCR between an amine (primary or secondary), an aldehyde (bearing a coordination site for boron atoms nearby the aldehyde group), and an aryl‐/vinyl‐boronic acid.^[^ ^33^, ^34^, ^35^, ^36^ ^]^ As the Petasis reaction is compatible with ─COOH, we used unprotected DO3A as the core of chelators, making our synthetic approach free of any protecting group. A glyoxylic acid or an alpha‐oxo aldehyde of the antenna group can provide the fourth ─COOH group or amide that can be used for developing either MRI contrast agents/radiopharmaceuticals or luminescent lanthanide probes. The arylboronic acid group introduces an α‐aryl‐substituent, which is beneficial to coordination stability, and provides the conjugating site for targeting vectors or serves as the targeting motif directly (Figure 1c). In this work, we successfully developed such a three‐component approach to assemble the required parts in a single‐step reaction, giving a library of valuable synthetic building blocks and potential target‐specific bio‐probes. Furthermore, we revealed the positive effect of the α‐aryl‐substituent on coordination kinetics and photophysical properties, in addition to the well‐known effect on coordination stability and relaxivity. Cellular imaging experiments with the luminescent lanthanide probes synthesized in this work demonstrated their potential for simultaneous multi‐color imaging of multiple biomarkers.

## Results and Discussion

**Figure 2:**
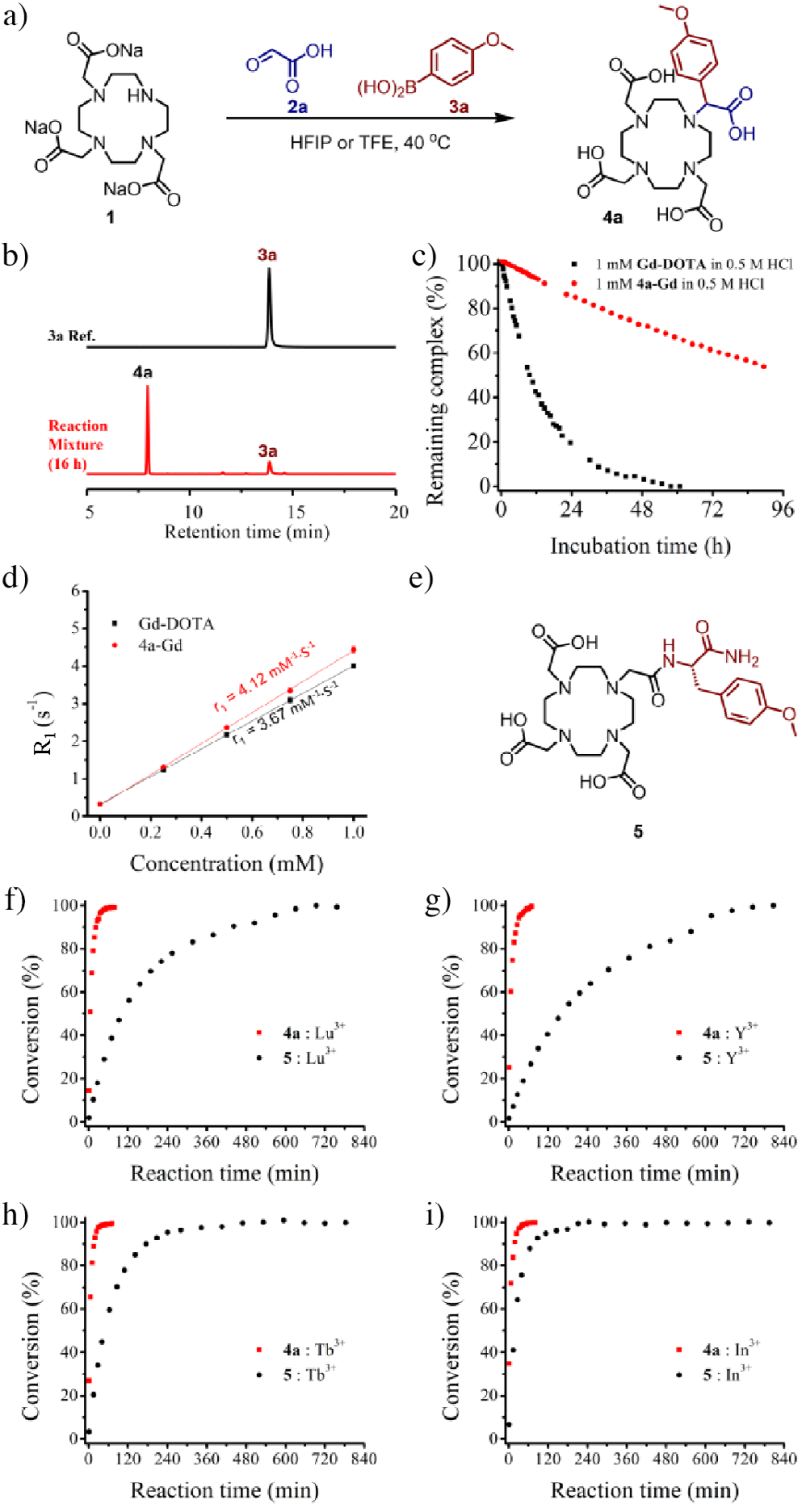
Synthesis and investigation of properties of α‐4‐methoxyphenyl‐substituted DOTA (**4a**). a) Synthetic scheme; b) HPLC chromatograms of reactant **3a** and the reaction mixture (HFIP, 40 °C, 16 h) showed the good conversion of the reaction; c) Comparison of the stability of **4a‐Gd** and **Gd‐DOTA** in 0.5 M HCl, the percentage of remaining complexes was determined by HPLC automatically; d) Comparison of relaxivity of **4a‐Gd** and **Gd‐DOTA** (B_0_ = 1.47 T (60 MHz) and T = 298 K); e) The structure of **5**, a DOTA mono‐amide was synthesized for comparison in labelling experiments; f)–i) Comparison of coordination kinetics of **4a** and **5** by labelling with non‐radioactive Lu^3+^, Y^3+^, Tb^3+^, and In^3+^.

At the beginning, we tried to react three commercially available reagents, sodium salt of unprotected DO3A (**1**), glyoxylic acid (**2a**), and 4‐methoxyphenylboronic acid (**3a**) in various solvents (Table S7). As a result, a quantitative conversion can be achieved to yield an α‐paramethoxyphenyl‐substituted DOTA (**4a**) when tetrafluoroethylene (TFE, 40 °C, 16 h) and hexafluoro‐2‐propanol (HFIP, 40 °C, 16 h) were used as solvents (Figure 2a,b). The isolated yield of **4a** from this single‐step reaction was 43%, which is far better than the overall yields of previously reported multi‐step approaches.^[^ ^28^, ^29^, ^37^, ^38^ ^]^ This approach completely avoids the use of protecting groups and repeated transformation of functional groups, significantly increasing the atom economy. Only one equivalent of nontoxic boronic acid will be generated as a by‐product.

To see if α‐aryl‐substituted DOTAs synthesized by our MCR approach are superior chelators for GBCAs, we prepared the corresponding Gd^3+^ complex **4a‐Gd** and verified its coordination stability and relaxivity. 1 mM **4a‐Gd** and FDA‐approved GBCAs **Gd‐DOTA** were incubated in 0.5 M HCl at 25 °C. The dissociation of the complexes was monitored automatically by HPLC (Figure 2c). As a result, around half of the **Gd‐DOTA** was dissociated after 12 h incubation, whereas less than 10% of **4a‐Gd** dissociated during the same period. **Gd‐DOTA** dissociated completely after incubating for around 60 h, while there is still around 50% of **4a‐Gd** remaining after 4 d. This experiment verified the largely improved stability of the α‐aryl‐substituted DOTA synthesized by our MCR approach. The T_1_ relaxivity of **4a‐Gd** was determined as 4.10 mM^−1^ S^−1^, around 11% higher than **Gd‐DOTA**’s 3.68 mM^−1^ S^−1^ measured under the same conditions (Figure 2d). The improved coordination stability and relaxivity is in accordance with previous works.^[^ ^28^, ^29^ ^]^

Apart from coordination stability, coordination kinetics is also a critical parameter for radiopharmaceuticals due to the limited half‐life of radioisotopes. An ideal chelator for radiopharmaceuticals should label radioactive metal ions quickly (to diminish the decay of radioisotopes before administration) under milder conditions (to avoid the decomposition of the agent during radiolabeling) at lower concentrations (as radiopharmaceuticals are always applied in extremely low dosages). The coordination kinetics of α‐substituted DOTAs has been less studied in previous work.^[^ ^39^, ^40^ ^]^ To see if the α‐aryl‐substituted DOTAs synthesized by our MCR approach are ideal chelators for radiopharmaceuticals, we systematically investigated the coordination kinetics of **4a** with a series of non‐radiative counterparts of commonly used radionuclides. A mono‐amide of DOTA conjugated with *O*‐methyl‐l‐tyrosine (**5**, Figure 2e) was prepared for comparison. The labelling experiments were conducted with 50 µM ligand (**4a** or **5**) and 500 µM metal ion in 0.5 M acetate buffer (pH 5.6), and the conversion was monitored automatically by HPLC. **4a** showed a significantly faster coordination rate compared to **5** for all four metal ions, including Lu^3+^, Y^3+^, Tb^3+^, and In^3+^ (Figure 2f–i). These experiments unprecedentedly demonstrate the superiority of α‐aryl‐substituted DOTAs synthesized by the MCR approach as potential chelators for radiopharmaceuticals.

**Figure 3:**
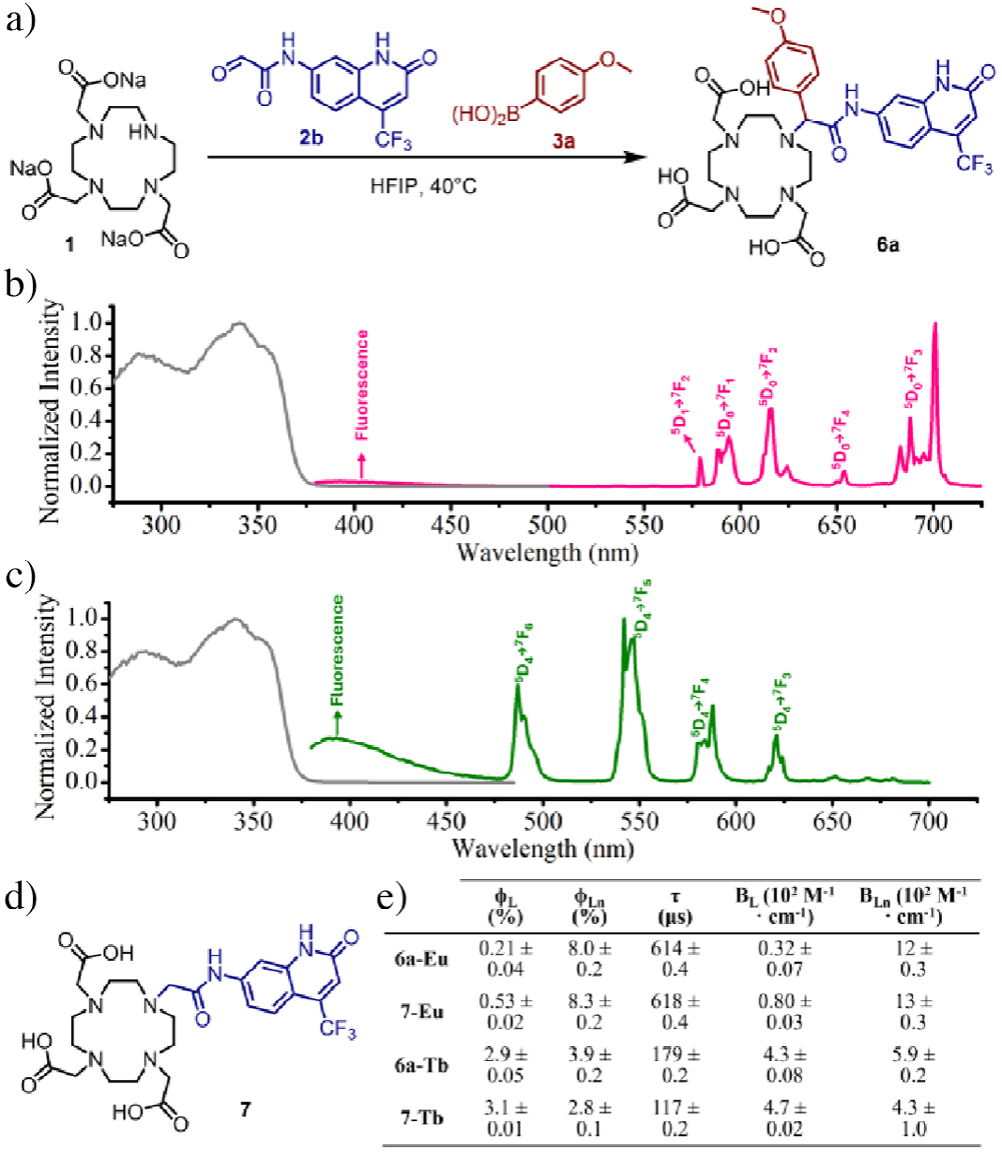
Synthesis and investigation of photophysical properties of α‐(4‐methoxyphenyl)‐substituted ligand for luminescent lanthanide complexes (**6a‐Ln**). a) Synthetic scheme for **6a**; b),c) Normalized emission (λ_exc_ = 342 nm, red and green) and excitation (grey) spectra of **6a‐Eu** (λ_emi_ = 616 nm for excitation spectrum) and **6a‐Tb** (λ_emi_ = 546 nm for excitation spectrum). d) Ligand for lanthanide complexes without α‐substituent (**7**) synthesized for the comparison of photophysical property; e) Comparison of photophysical properties of Eu and Tb complexes with **6a** and **7** (λ_exc_ = 342 nm); B values have been calculated with ε(342) = 15 167 M^−1^ cm^−1^ from ref. [32]. Uncertainties are statistical errors based on at least 3 repeat measurements (reproducibility); experimental errors are larger: 5%–10% on ϕ values, and 2%–3% on τ values.

We tried to extend our synthetic approach to luminescent lanthanide complexes. The well‐known antenna 7‐amino‐4‐trifluoromethyl‐2‐(*1H*)‐quinolinone (CS124‐CF_3_) was chosen, and the corresponding building block was modified as an α‐oxoaldehyde counterpart (**2b**) by simple treatment (Scheme S1). The desired product **6a** was obtained in a considerable yield (Figure 3a) under the same conditions as for synthesizing **4a**, and we further metalated it to yield complexes **6a‐Eu** and **6a‐Tb**. Both showed typical emission patterns as expected (Figure 3b,c). The corresponding complexes without an α‐substituent were also synthesized for comparison (**7‐Eu and 7‐Tb**). No difference in absorption and emission patterns was found between **6a‐Eu/Tb** and **7‐Eu/Tb**, while the α‐aryl‐substituted product **6a‐Tb** showed modest improvement in photophysical properties. The luminescent quantum yields of **7‐Tb** and **6a‐Tb** are 2.8 ± 0.1% and 3.9 ± 0.1%, respectively, and their luminescence lifetimes are 117 ± 0.2 µs and 179 ± 0.2 µs. Compared with the Tb complex without an α‐substituent, introducing such a substituent led to ∼40% and ∼50% improvement in the quantum yield and lifetime, respectively, proving the positive effect of the α‐substituent. For the Eu complexes **7‐Eu** and **6a‐Eu**, no significant difference in the quantum yield and lifetime was observed. Ln‐centered NIR luminescence was also observed for Nd, Sm, and Yb complexes (Figures S3–S6). Luminescent lanthanide probes with α‐substituent were not reported in previous studies. Our MCR approach provides an easy way to such a new layout of luminescent lanthanide probes, and suitable modifications of the α‐substituent should result in improved photophysical properties.

**Figure 4:**
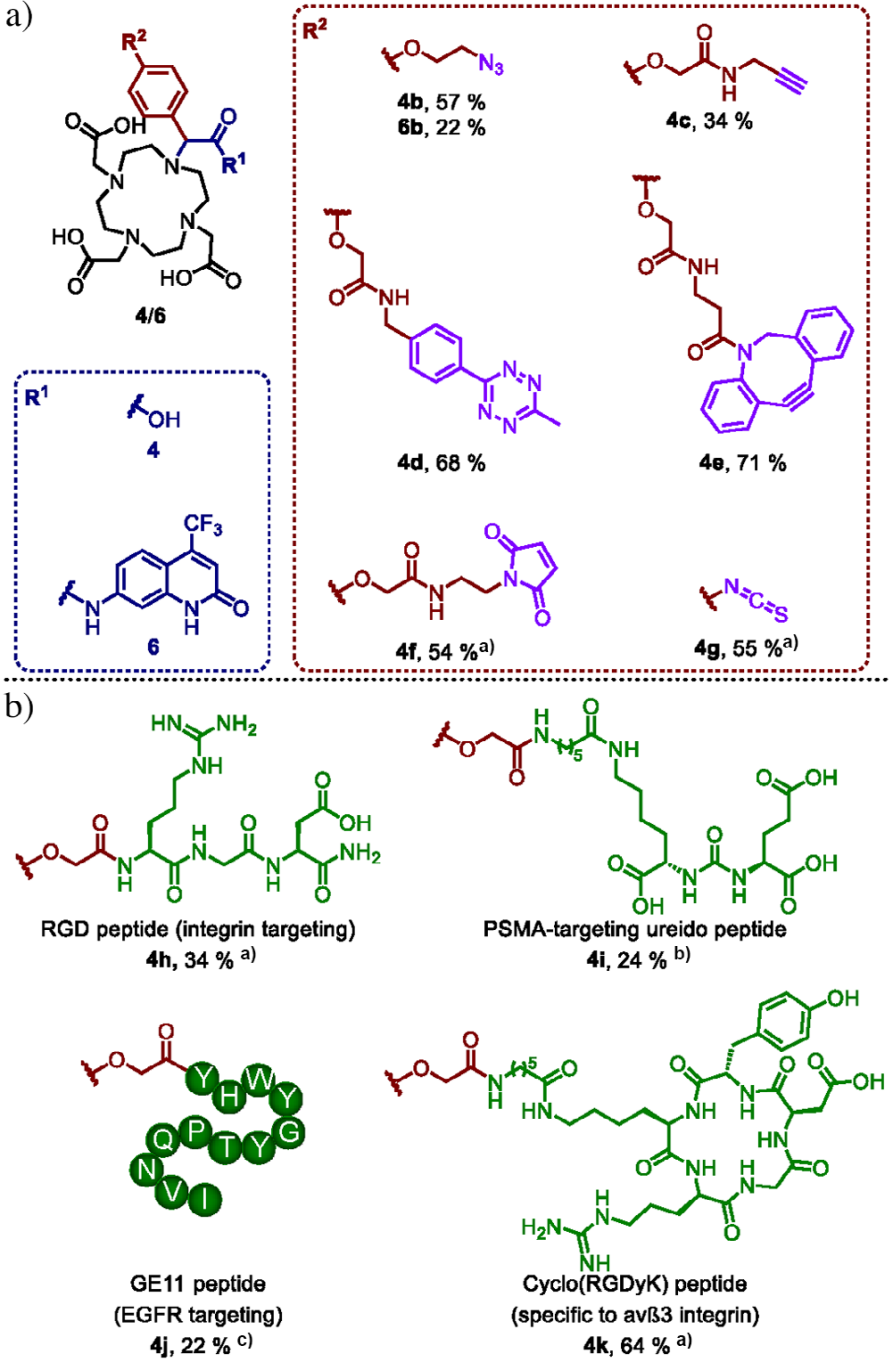
The synthetic building blocks of α‐aryl‐substituted DOTA prepared by MCR approach and their applications in bioconjugation. The percentages represent the isolated yield of the corresponding product. a) Examples of synthetic building blocks with functional group for bioconjugation. ^a)^Additional steps are required for the desired product; b) The examples of direct peptide functionalization with our MCR approach. ^a)^Purified boronopeptide was used for the reaction; ^b)^Boronopeptide was prepared by solution‐phase conjugation, and the crude reaction mixture was used directly for the reaction; ^c)^Boronopeptide was prepared by solid‐phase conjugation, and the crude post‐cleavage mixture was used directly for the reaction.

In addition to the positive effect on coordination, magnetic, and photophysical properties, α‐substituents are also prone to bear targeting vectors or functional groups for bioconjugation. We therefore synthesized a series of building blocks by the MCR approach (Figure 4a). A series of α‐aryl‐substituted DOTAs (**4**) and α‐aryl‐substituted luminescent lanthanide complexes (**6**) with extra functional groups as their α‐substituent were obtained. The azido‐ (**4b** & **6b**), alkyne‐ (**4c**), tetrazine‐ (**4d**), and DBCO‐ (**4e**) containing building blocks can be prepared by simply replacing 4‐methoxyphenylboronic acid (**3a**) with the corresponding arylboronic acid, as in the synthesis of **4a** and **6a**. For maleimide‐ (**4f)** and isothiocyanate‐ (**4g**) containing building blocks, further functional group transformation was required after the Petasis reaction (Schemes S5 and S6). Furthermore, we also used boronopeptides in the MCR to give α‐aryl‐substituted DOTAs with targeting peptides to get the corresponding peptide conjugates in considerable yields (Figure 4b), including arginylglycyl aspartic acid (RGD) peptide (**4h**), prostate‐specific membrane antigen (PSMA) targeting ureido peptide (**4i**), epidermal growth factor receptor (EGFR) targeting peptide GE11 (**4j**), and integrin α_v_β3‐targeting peptide *cyclo*(RGDyK) (**4k**).

**Figure 5:**
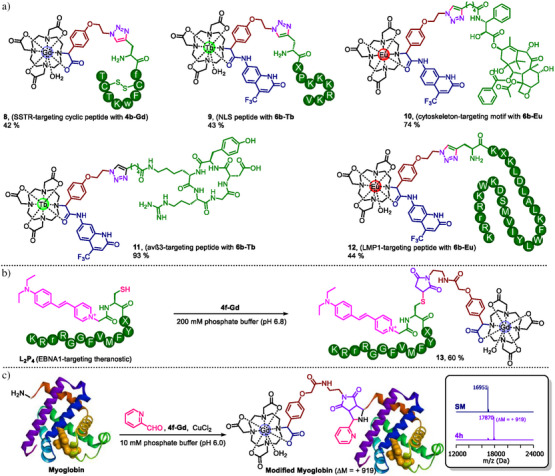
The application of synthesized α‐aryl‐substituted DOTA building blocks for bioconjugation. a) The examples of peptide functionalization by CuAAC with azide‐containing building blocks. b) Functionalization of peptide **L2P4** with maleimide‐containing building block by thiol‐Michael addition; c) Functionalization of the N‐terminal of protein myoglobin with maleimide‐containing building blocks by copper‐catalyzed [3 + 2] cycloaddition. Deconvoluted mass spectrum (ESI) showed complete conversion of the protein to the desired product.

To demonstrate the application of the above‐synthesized building blocks, we functionalized them with biomolecules. For example, the Gd complex of azido‐containing building block **4b‐Gd** can be clicked to alkyne‐containing somatostatin receptor (SSTR) targeting cyclic peptide to give an analogue of DOTATATE **8** by the copper‐catalyzed azide‐alkyne cycloaddition (CuAAC) (Figure 5a). Similarly, the Tb complex of azido‐containing building block **6b‐Tb** was clicked to alkyne‐containing nuclear localization signal (NLS) peptide and α_v_β_3_‐targeting peptide, respectively, to give the potential luminescent probes **9** and **11**, while the Eu counterpart **6b‐Eu** was clicked to alkyne‐containing docetaxel derivative and LMP1‐targeting peptide P19, respectively, to give **10** and **12**. As another example, the Gd complex of maleimide‐containing building block **4f‐Gd** was reacted with **L_2_P_4_**, an EBNA1‐targeting theranostic agent reported in our previous study, to give a potential EBNA1‐targeting fluorescent and MR dual‐modal probe **13** (Figure 5b).^[^ ^14^, ^41^, ^42^, ^43^ ^]^ We demonstrated the functionalization of protein myoglobin with maleimide building block by using a recently reported copper‐catalyzed [3 + 2] cycloaddition (Figure 5c)^[^ ^44^ ^]^ to achieve quantitative conversion. These examples show exciting potential for the MCR protocol to achieve valuable synthetic building blocks for bioconjugation, which is vital for the development of targeting therapeutic and diagnostic agents.

**Figure 6:**
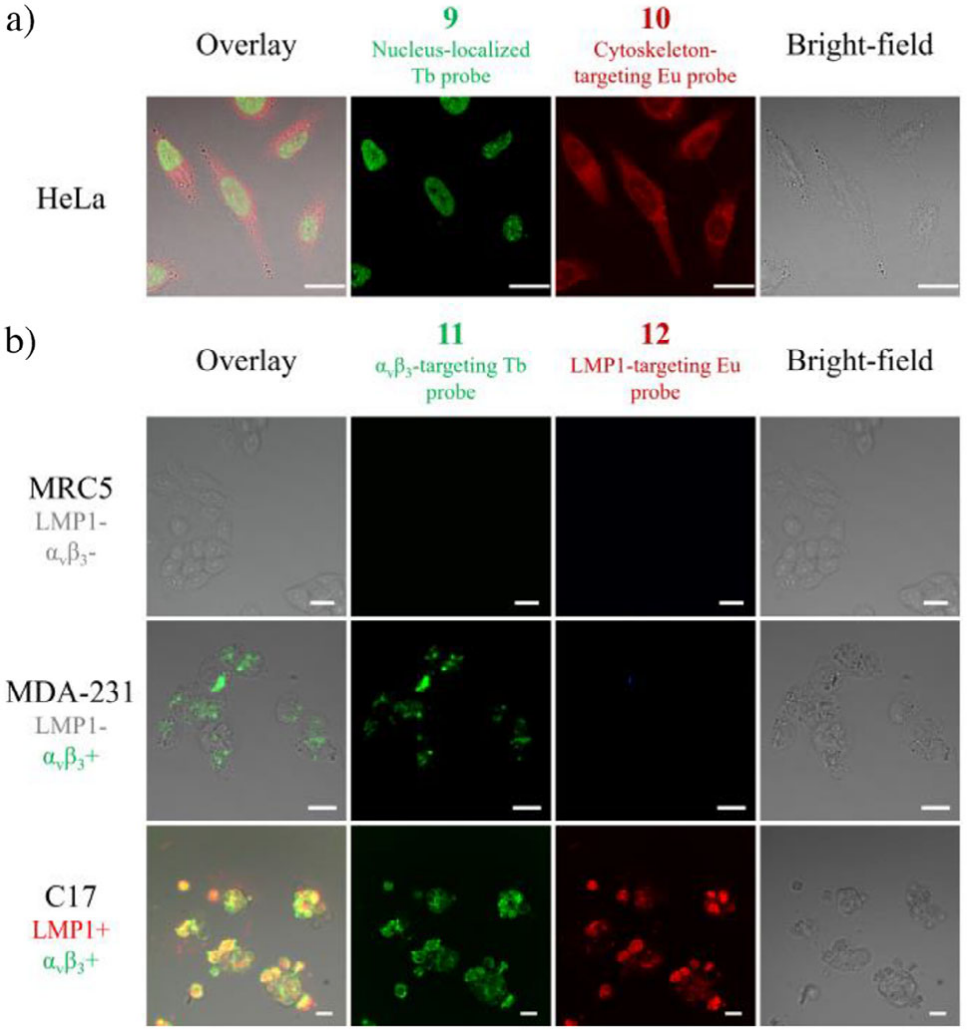
The application of synthesized probes **9**–**12** in cellular imaging (scale bar: 20 µm, concentration of probe: 10 µM). a) Co‐imaging of nucleus‐localized Tb probe **9** and cytoskeleton‐localized Eu probe **10** in HeLa cell (4 h incubation); b) Co‐imaging of α_v_β_3_‐targeting Tb probe **11** and LMP1‐targeting Eu probe **12** in various cell lines.

Finally, we conducted a proof‐of‐concept cellular imaging experiment involving the targeting of luminescent lanthanide probes **9**–**12**. Probes **9** and **10** were used for cellular imaging of the HeLa cell line (Figure 6a). After 4 h of incubation, **9** was localized into the nucleus, while **10** was distributed in the cytoplasm with fiber‐like objects whose appearance was in accordance with microtubule bundles. The behaviors of **9** and **10** were very similar to the probes with the same targeting motif for nucleus^[^ ^45^, ^46^, ^47^ ^]^ and cytoskeleton^[^ ^48^, ^49^ ^]^ in several previously published works. For probes **11** and **12**, their specificity for α_v_β_3_ and LMP1 oncoprotein was verified first individually (Figure S7) and then together in a co‐imaging experiment with various cell lines, displaying different luminescent signals and targeting specificities (Figure 6b). In the MRC5 cell line, which expressed neither α_v_β_3_ nor LMP1 oncoprotein, no signal was observed. On the contrary, for the MDA‐231 cell line with α_v_β_3_ but not LMP1 overexpression, only Tb luminescence from **11** was found. For C17 cell line, both α_v_β_3_ and LMP1 are overexpressed, and signals from both probes were detected. These experiments demonstrated the potential of the synthesized probes to visualize different cellular organelles and to differentiate various types of cell lines by multi‐color imaging techniques.

## Conclusions

In this work, we present a universal one‐step, protecting‐group‐free strategy based on the concept of multicomponent reactions for synthesizing bioconjugated/bioconjugatable α‐aryl‐substituted DOTAs. For decades, the adoption of α‐substituted DOTAs for the development of diagnostic and therapeutic agents has been limited by their tedious synthetic procedure, despite the superior stability and relaxivity of their Gd complexes. Until now, DOTA mono‐amides have been the preferred choice for both industry and researchers since they were considered the cheapest pathway to DOTA‐based targeting bioagents, albeit at the cost of reduced coordination stability. Fortunately, such a compromise is no longer needed, as the new approach presented here for the synthesis of α‐aryl‐substituted DOTAs no longer requires multi‐step preparation with protection, deprotection, and transformation of functional groups. Remarkably, α‐aryl‐substituted DOTAs can now be synthesized more easily than DOTA mono‐amides.

By uniting synthetic simplicity with multifunctional strategy, this work redefines the application field of α‐aryl‐substituted DOTAs. These chelating agents not only enable rapid radiolabeling but also deliver enhanced relaxivity and photophysical properties, including NIR emission. Exploiting the versatility of the MCR synthetic platform, we have generated chelators and complexes with diverse functional groups well suited for bioconjugation with a broad range of biomolecules—including drugs, peptides, and proteins. Selected probes, integrating specific targeting motifs and luminescent lanthanide ions, were used for multi‐color cellular imaging, demonstrating their potential in biomedical applications. We believe the new simplified MCR route transforms the synthesis of α‐aryl‐substituted DOTAs, opening the door to next‐generation, high‐performing theranostics/bioprobes and their associated biological and medical applications.

## Conflict of Interests

The authors declare no conflict of interest.

## Supporting information



Supporting Information

## Data Availability

The data that support the findings of this study are available in the Supporting Information of this article.
