# Supplementary material for: One‐Step Assembly of α‐Aryl‐Substituted DOTAs as Superior and Universal Platforms for Multifunctional Theranostics
Source: Angew Chem Int Ed Engl. 2025 Oct 15;64(50):e202519204. doi: 10.1002/anie.202519204 (PMC12684321; doi:10.1002/anie.202519204)
Supplement: Supplementary file 1 — Supporting Information [file ANIE-64-e202519204-s001.docx]

One-step Assembly of α-Aryl-substituted DOTAs as Superior and Universal Platforms for Multifunctional Theranostics

Pak-Lun Lam^[a]+^, Yue Wu^[a]+^*, Lijia Yao^[a]^, Hei-Yui Kai^[a]^, Ho-Fai Chau^[a]^, Qian Zhang^[a]^, Wanqi Zhou^[a][b]^, Jean-Claude G. Bünzli^[a][c]^*, Ka-Leung Wong^[a]^*

[a] P.-L. Lam, Dr Y. Wu, L. Yao, H.-Y. Kai, H.-F. Dr Chau, Q. Zhang, W. Zhou, Prof. Dr J.-C. G. Bünzli, Prof. Dr K.-L. Wong
Department of Applied Biology and Chemical Technology,
The Hong Kong Polytechnic University,
Hung Hom, Hong Kong SAR, China.
E-mail: [klgwong@polyu.edu.hk](mailto:klgwong@polyu.edu.hk) (Prof. K.-L. Wong); [kurt-yue.wu@polyu.edu.hk](mailto:kurt-yue.wu@polyu.edu.hk) (Dr. Y. Wu)

[b] W. Zhou

Department of Chemistry,

Hong Kong Baptist University,

224 Waterloo Rd, Kowloon Tong, Kowloon, Hong Kong SAR, China.

[c] Prof. Dr J.-C. G. Bünzli

Institute of Chemical Sciences and Engineering (ISIC)

Swiss Federal Institute of Technology (EPFL)

CH-1015 Lausanne, Switzerland

E-mail: [jean-claude.bunzli@epfl.c](mailto:jean-claude.bunzli@epfl.c)h

+ P.-L. Lam and Y. Wu contributed equally to this work.

Supporting information for this article is given via a link at the end of the document.

# Supporting information

# Content

[Content 1](#_Toc204867825)

[Chemistry 3](#_Toc204867826)

[Reagents & Instruments 3](#_Toc204867827)

[Synthesis and characterization of compounds 7](#_Toc204867828)

[Coordination stability, kinetics, and magnetic resonance experiments 50](#_Toc204867829)

[Photophysical experiments 51](#_Toc204867830)

[Biological experiments 54](#_Toc204867831)

[Reference 55](#_Toc204867832)

[HPLC chromatograms and HRMS spectra of products 56](#_Toc204867833)

[NMR spectra of products 142](#_Toc204867834)

# Chemistry

## Reagents & Instruments

Sources of reagents:

All amino acid building blocks for Fmoc-SPPS were purchased from Bidepharm. All peptides used for SPPS were built on Rink Amide resins that were purchased from GL Biochem(Shanghai) Ltd. Most organic and inorganic compounds were purchased from Bidepharm and Dieckmann, respectively, unless otherwise specified. DIPEA was purchased from Energy. TFE and HFIP were purchased from Macklin. Tris-*tert*-butyl 1,4,7,10-tetraazacyclododecane-1,4,7-triacetate (*tris*-*^t^*Bu-DO3A) and 7-Amino-4-(trifluoromethyl)quinolin-2(1*H*)-one (CS124^CF3^) were synthesized according to previous literature.^1,2^ All other reagents and solvents were purchased from commercial sources and used without further purification.

HPLC systems:

**Analytical HPLC:** High-performance liquid chromatography for analytical purposes was carried out on an Agilent 1260 Infinity II LC system equipped with a G7112B Binary Pump, a G7129A Vialsampler, a G7115A Diode Array Detector (DAD), and an Agilent HC-C18(2) reverse phase HPLC column of inner diameter 4.6 mm, length 250 mm, and particle size 5 µm. The instrument was purchased from Agilent Technologies. The mobile phases were Milli-Q water and acetonitrile acidified with 0.1% TFA. The gradients are shown below:

Table S1. Gradient I for analytical HPLC.

| **Time**  **(minute)** | **A%**  **(Milli-Q water + 0.1% TFA)** | **B%**  **(MeCN + 0.1% TFA)** | **Flow**  **(mL/min)** |
| --- | --- | --- | --- |
| 0 | 99 | 1 | 1 |
| 20 | 90 | 10 | 1 |
| 21 | 0 | 100 | 1 |
| 26 | 0 | 100 | 1 |
| 27 | 99 | 1 | 1 |
| 30 | 99 | 1 | 1 |

Table S2. Gradient II for analytical HPLC.

| **Time**  **(minute)** | **A%**  **(Milli-Q water + 0.1% TFA)** | **B%**  **(MeCN + 0.1% TFA)** | **Flow**  **(mL/min)** |
| --- | --- | --- | --- |
| 0 | 98 | 2 | 1 |
| 20 | 80 | 20 | 1 |
| 21 | 0 | 100 | 1 |
| 26 | 0 | 100 | 1 |
| 27 | 98 | 2 | 1 |
| 30 | 98 | 2 | 1 |

Table S3. Gradient V for analytical HPLC.

| **Time**  **(minute)** | **A%**  **(Milli-Q water + 0.1% TFA)** | **B%**  **(MeCN + 0.1% TFA)** | **Flow**  **(mL/min)** |
| --- | --- | --- | --- |
| 0 | 95 | 5 | 1 |
| 20 | 50 | 50 | 1 |
| 21 | 0 | 100 | 1 |
| 26 | 0 | 100 | 1 |
| 27 | 95 | 5 | 1 |
| 30 | 95 | 5 | 1 |

Table S4. Gradient X for analytical HPLC.

| **Time**  **(minute)** | **A%**  **(Milli-Q water + 0.1% TFA)** | **B%**  **(MeCN + 0.1% TFA)** | **Flow**  **(mL/min)** |
| --- | --- | --- | --- |
| 0 | 90 | 10 | 1 |
| 20 | 40 | 60 | 1 |
| 21 | 0 | 100 | 1 |
| 26 | 0 | 100 | 1 |
| 27 | 90 | 10 | 1 |
| 30 | 90 | 10 | 1 |

Table S5. Gradient XX for analytical HPLC.

| **Time**  **(minute)** | **A%**  **(Milli-Q water + 0.1% TFA)** | **B%**  **(MeCN + 0.1% TFA)** | **Flow**  **(mL/min)** |
| --- | --- | --- | --- |
| 0 | 80 | 20 | 1 |
| 20 | 20 | 80 | 1 |
| 21 | 0 | 100 | 1 |
| 26 | 0 | 100 | 1 |
| 27 | 80 | 20 | 1 |
| 30 | 80 | 20 | 1 |

Table S6. Gradient XXX for analytical HPLC.

| **Time**  **(minute)** | **A%**  **(Milli-Q water + 0.1% TFA)** | **B%**  **(MeCN + 0.1% TFA)** | **Flow**  **(mL/min)** |
| --- | --- | --- | --- |
| 0 | 70 | 30 | 1 |
| 20 | 0 | 100 | 1 |
| 21 | 0 | 100 | 1 |
| 26 | 0 | 100 | 1 |
| 27 | 70 | 30 | 1 |
| 30 | 70 | 30 | 1 |

**Preparative HPLC:** Purification of crude products was performed, according to the need, after every reaction, on either instrument (a) or (b). The details of (a) and (b) are as follows:

1. An Agilent 1260 Infinity II Preparative LC System, which was composed of a G7157A Preparative AutoSampler, a G7161A Preparative Binary Pump, a G7115A DAD, a G1364E Fraction collector, and an Agilent 5 Prep C18 column of inner diameter 21.2 mm, length 50 mm, and particle size 5 μm. It was operated at a flow rate of 21.24 ml/min. The gradients were analogous to those for analytical HPLC, but those at the first 20 minutes were scaled to 4 minutes.
2. A Waters semi-preparative system, which was composed of a Waters 2707 Autosampler, a Waters 1525 Binary HPLC Pump, a Waters 2998 Photodiode Array (PDA) Detector, a Waters Fraction Collector, and a Waters Altantis dC18 T3 Prep OBD^TM^ column of inner diameter 19 mm, length 250 mm, and particle size 5 μm. It was operated at a flow rate of 8.53 ml/min while the gradients of the analytical HPLC at the first 20 minutes were scaled to 40 minutes.

The fractions were identified by LC/MS before collection.

Mass Spectrometry:

Preliminary identification of compounds through mass-to-charge ratio (m/z) was conducted by an Agilent analytical HPLC with most equipment the same as the aforementioned one but with an Agilent Poroshell 120 EC-C18 Threaded Column of inner diameter 3 mm, length 150 mm, and particle size 2.7 μm instead and an Agilent G6125B LC/MSD coupled after DAD. High-resolution mass spectra were obtained from either (a) Agilent 6460 Triple Quadrupole LC/MS or (b) Bruker UltrafleXtreme MALDI-TOF/TOF MS.

Nuclear Magnetic Resonance Spectroscopy:

The NMR spectra reported were obtained from either a Bruker Avance-III 400 MHz FT-NMR or a Bruker Avance-III 600 MHz FT-NMR spectrometer system. The ^1^H and ^13^C chemical shifts were referenced to corresponding solvent peaks DMSO-*d*_6_ at 2.50 ppm and 39.52 ppm, respectively. Peak multiplicities were abbreviated as the following: s = singlet; d = doublet; t = triplet; dd = doublet of doublets; m = multiplet; br = broad.

## Synthesis and characterization of compounds

General procedures of solid-phase peptide synthesis:

Fmoc-SPPS was performed manually at ambient temperature with Torviq Plastic reaction vessels with frits, designed for solid-phase organic reactions. The Rink AM resins were swollen in DMF before the reaction proceeded. Unless specified, every portion of 0.1 mmol peptide-bound resins was treated with 4 ml of solvent. The Fmoc protecting group on the resin was removed by shaking the resin with 20% (v/v) 4-methylpiperidine in DMF for 2 h. The first amino acid was incorporated by shaking the resin with a mixture of Fmoc-protected amino acids (4 equiv.), PyBOP (4 equiv.), and DIPEA (8 equiv.) in DMF overnight. The resins were washed thoroughly with DMF three times. Then, the *N*-Fmoc protecting group on the peptide’s terminal was removed by shaking the resin with 20% (v/v) 4-methylpiperidine in DMF for 30 min. The resins were washed thoroughly with DMF three times afterward. The procedures of subsequent amino acid incorporations (2 – 24 h) and Fmoc deprotections (30 min) were repeated, with the conditions and reagents the same as those for the first amino acid, until the desired peptide sequence was obtained. The resins were dried after three thorough washings with DCM, followed by another three with Et_2_O before the resins were dried under a high vacuum. The peptide’s substitution value on the resin was determined according to the reported protocol.

Incorporation of phenylboronic acid building block onto the resin-bound peptides. Phenylboronic acid building block S4 or S6 (4 equiv.), PyBOP (4 equiv.), and DIPEA (12 equiv.) in DMF (4 mL for each 0.1 mmol resin) were shaken with peptide-bound resins in DMF for 2 to 24 h. The post-treatment followed generalized procedures.

**Global cleavage of the peptide from the Rink AM resins.** The dried resins were treated with a solution of TFA/TIPS/H_2_O, v/v/v, 95/2.5/2.5 (4 mL for each 0.1 mmol resin), for 2 h. The solution was diluted with a 10-times-volume of Et_2_O to allow the peptide’s precipitation. The precipitation was repeatedly concentrated and re-suspended in the same volume of Et_2_O two times. The pellet was dried in a high vacuum before further processing.

Synthetic procedures and characterization data of aldehyde-bearing antenna:

**Scheme S1.** The synthetic route of Oxo-Ac-CS124^CF3^ **2b**.

**Boc-Ser(*^t^*Bu)-CS124^CF3^ S1.** To the solution of Boc-Ser(*^t^*Bu)-OH (1.31 g, 5.0 mmol) and DEPBT (1.65 g, 5.5 mmol) in THF (25 ml), DIPEA (1.92 ml, 11 mmol) was added. The reaction mixture was stirred for 10 minutes before CS124^CF3^ (1.37 g, 6.0 mmol) was added. The reaction mixture was stirred for 16 hrs at 66℃. The reaction mixture was diluted with DI water (250 ml). The precipitate was collected and washed with DI water (3 × 50 ml). Complete desiccation yielded the crude product **S1** (1.76 g), which proceeded to the next step without further purification. Several mg of **S1** were further purified via HPLC for characterization. White solid. Analytical HPLC: Gradient XX, retention time: 18.3 min. ^1^H NMR (600 MHz, DMSO-*d*_6_) δ 12.29 (s, 1H), 10.42 (s, 1H), 7.99 (d, *J* = 2.1 Hz, 1H), 7.65 (d, *J* = 8.8 Hz, 1H), 7.41 (dd, *J* = 8.9, 2.1 Hz, 1H), 6.89 (d, *J* = 7.9 Hz, 1H), 6.82 (s, 1H), 4.23 (dd, *J* = 6.6 Hz, 1H), 3.58 – 3.44 (m, 2H), 1.38 (s, 9H), 1.08 (s, 9H). ^13^C NMR (101 MHz, DMSO-*d*_6_) δ 170.14, 160.41, 155.21, 141.68, 140.81, 136.30 (q, *J* = 30.5 Hz), 124.94, 122.58 (q, *J* = 275.3 Hz), 114.88, 108.82, 105.07, 78.36, 72.95, 61.78, 55.76, 28.17, 27.18. HRMS (ESI-QTOF): calc. for C_22_H_29_F_3_N_3_O_5_^+^ [M+H]^+^ 472.2054, found 472.2057.

**H-Ser-CS124^CF3^ S2.** The crude **S1** (864 mg) was stirred in a solution of TFA/TIPS/H_2_O, v/v/v, 95/2.5/2.5 (26 ml), for 4 hrs. The solution was diluted with Et_2_O (130 ml). The precipitate was collected and washed with Et_2_O (2 × 20 ml). The precipitate was dissolved in 0.1% TFA (aq) on the filter. Completely drying the filtrate yielded the TFA salt of crude product **S2** (643 mg), which proceeded to the next step without further purification. Several mg of **S2** were further purified via HPLC for characterization. White solid. Analytical HPLC: Gradient V, retention time: 13.1 min. ^1^H NMR (400 MHz, DMSO-*d*_6_) δ 12.39 (s, 1H), 10.92 (s, 1H), 8.32 (s, 3H), 7.99 (d, *J* = 2.0 Hz, 1H), 7.82 – 7.60 (m, 2H), 7.41 (dd, *J* = 9.0, 2.1 Hz, 1H), 6.85 (s, 1H), 4.05 (d, *J* = 4.5 Hz, 1H), 4.01 – 3.75 (m, 3H). ^13^C NMR (151 MHz, DMSO-*d*_6_) δ 166.57, 160.68, 141.10, 140.90, 136.50 (q, *J* = 30.8 Hz), 125.36, 122.67 (q, *J* = 275.7 Hz), 120.01, 115.07, 109.50, 105.59, 60.28, 55.36. HRMS (ESI-QTOF): calc. for C_13_H_13_F_3_N_3_O_3_^+^ [M+H]^+^ 316.0904, found 316.0903.

**Oxo-Ac-CS124^CF3^ 2b.** The crude TFA salt of **S2** (609 mg) was stirred with sodium periodate (609 mg) in 100 mM sodium phosphate buffer (pH = 7) for 2 hrs. The precipitate was collected and washed with DI water (4 ml × 3) and MeOH (1 ml × 2). Complete desiccation of precipitate resulted in **2b** (622 mg, purity by weight: 64%) for further use without further purification. Overall yield: 60%. White solid. Analytical HPLC: Gradient X, retention time: 12.2 min. ^1^H NMR (400 MHz, 5% D_2_O in Methanol-*d*_4_) δ 8.17 (d, *J* = 2.1 Hz, 1H), 7.77 (dd, *J* = 9.0, 2.1 Hz, 1H), 7.44 (dd, *J* = 9.0, 2.1 Hz, 1H), 6.91 (s, 1H), 5.00 (s, 1H). ^13^C NMR (101 MHz, 5% D_2_O in Methanol-*d*_4_) δ 170.31, 163.52, 142.49, 141.78, 139.73 (q, *J* = 31.5 Hz), 126.57, 123.92 (q, *J* = 274.7 Hz), 119.92 (q, *J* = 5.5 Hz), 117.23, 111.91, 107.49, 95.53. HRMS (ESI-QTOF): calc. for C_12_H_10_F_3_N_2_O_4_^+^ [M+H_2_O+H]^+^ 303.0587, found 303.0593.

Synthetic procedures and characterization data of phenylboronic acid building blocks:

**Scheme S2.** The synthetic route of acetic acid **S4**.

***t*-Butyl ester S3.** Upon vigorous stirring, *t*-butyl bromoacetate (3.85 ml, 26 mmol) was added dropwise to the mixture of 4-hydroxyphenylboronic acid (2.76 g, 20 mmol) and caesium carbonate (7.82 g, 24 mmol) in DMF (40 ml) at rt. The reaction mixture was then stirred for 16 hrs at 80°C. Having cooled and then concentrated, the reaction mixture was re-diluted in EA and then filtered. The filtrate was concentrated to 20 ml and then diluted with *n*-hexane (160 ml) to induce precipitation. The collected precipitate was washed with *n*-hexane/DCM, v/v, 8/1 (50 ml × 2), followed by DI water (50 ml × 2). Complete desiccation yielded crude **S3** (3.25 g), which proceeded to the next step without further purification. Pale brown solid. Analytical HPLC: Gradient XXX, retention time: 9.9 min. ^1^H NMR (400 MHz, DMSO-*d*_6_) δ 7.88 (s, 1H), 7.71 (d, *J* = 8.7 Hz, 1H), 6.84 (d, *J* = 8.7 Hz, 1H), 4.65 (s, 1H), 1.42 (s, 4H). ^13^C NMR (101 MHz, DMSO-*d*_6_) δ 167.84, 159.33, 135.77, 113.41, 81.38, 64.70, 27.70. HRMS (ESI-QTOF): calc. for C_24_H_36_B_2_NO_9_^+^ [2M-H_2_O+NH_4_]^+^ 504.2571, found 504.2576; calc. for C_24_H_32_B_2_NaO_9_^+^ [2M-H_2_O+Na]^+^ 509.2125, found 509.2131.

**Acetic acid S4.** The solution of **S3** (2.08 g) and TIPS (4.38 ml) in TFA/DCM, v/v, 1/1 (70 mL) was stirred for 15 mins. The reaction mixture was diluted with Et_2_O (350 ml) for precipitation. The collected precipitate was washed with DCM (50 ml × 2). Removing the solvent yielded nearly pure **S4** (2.45 g, purity by weight: 65%). The product was quantified by the analytical HPLC with 4-methoxyphenylboronic acid as an internal standard. The crude product was used without further purification. Pale brown solid. Two-step yield: 64%. Analytical HPLC: Gradient V, retention time: 10.2 min. ^1^H NMR (400 MHz, DMSO-*d*_6_) δ 13.01 (s, 1H), 7.88 (s, 4H), 7.71 (d, *J* = 8.6 Hz, 2H), 6.85 (d, *J* = 8.7 Hz, 2H), 4.68 (s, 2H). ^13^C NMR (101 MHz, DMSO-*d*_6_) δ 170.31, 159.48, 135.77, 113.41, 64.38. HRMS (ESI-QTOF): calc. for C_8_H_8_BO_5_^-^ [M-H]^-^ 195.0470, found 195.0469.

**Scheme S3.** The synthetic route of acetic acid **S6**.

**Methyl hexanoate S5.** The crude **S4** (1.40 g, 4.64 mmol) was stirred with DEPBT (2.56 g, 8.57 mmol) and DIPEA (6.22 ml, 35.7 mmol) in MeCN (35 ml) for 5 mins. Methyl 6-aminohexanoate hydrochloride (1.69 g, 9.29 mmol) for 16 hrs. Having concentrated, the reaction mixture was re-diluted in DI water (40 ml) for precipitation. The collected precipitate was washed” with DI water (40 ml × 3). Complete desiccation of the precipitate gave pure **S5** (1.32 g), which was used without further purification. White solid. Analytical HPLC: Gradient V, retention time: 16.9 min. ^1^H NMR (400 MHz, DMSO-*d*_6_) δ 8.07 (t, *J* = 5.8 Hz, 1H), 7.88 (s, 5H), 7.73 (d, *J* = 8.6 Hz, 2H), 6.90 (d, *J* = 8.7 Hz, 2H), 4.46 (s, 2H), 3.57 (s, 3H), 3.10 (q, *J* = 6.8 Hz, 2H), 2.27 (t, *J* = 7.4 Hz, 2H), 1.50 (p, *J* = 7.4 Hz, 2H), 1.41 (p, *J* = 7.3 Hz, 2H), 1.29 – 1.13 (m, 2H). ^13^C NMR (151 MHz, DMSO-*d*_6_) δ 173.36, 167.43, 159.42, 135.79, 113.71, 66.80, 51.22, 38.08, 33.21, 28.75, 25.78, 24.14. HRMS (ESI-QTOF): calc. C_16_H_23_BNO_8_^-^ [M+FA-H]^-^ for 368.1522, found 368.1527; calc. C_17_H_25_BNO_8_^-^ [M-OH+OMe+FA-H]^-^ for 382.1679, found 382.1688.

**Hexanoic acid S6**. The crude **S5** (759 mg) was stirred in 1M NaOH (aq)/MeOH, v/v, 1/1 (48 ml) for 16 hrs. After MeOH had been removed, the mixture was acidified with 1M HCl to pH 1 for precipitation. The precipitate was washed with DI water (5 ml × 3). Complete desiccation of the precipitate gave **S6** (743 mg, purity by weight: 74%), which was used without further purification. The product was quantified by the analytical HPLC with 4-methoxyphenylboronic acid as an internal standard. White solid. Two-step yield: 67%. Analytical HPLC: Gradient V, retention time: 13.5 min. ^1^H NMR (600 MHz, DMSO-*d*_6_) δ 12.00 (s, 1H), 8.07 (t, *J* = 5.9 Hz, 1H), 7.88 (s, 3H), 7.81 – 7.50 (m, 3H), 7.12 – 6.57 (m, 3H), 4.46 (s, 3H), 3.10 (q, *J* = 7.0 Hz, 3H), 2.18 (t, *J* = 7.4 Hz, 3H), 1.48 (p, *J* = 7.4 Hz, 2H), 1.42 (p, *J* = 7.6 Hz, 2H), 1.28 – 1.18 (m, 3H). ^13^C NMR (151 MHz, DMSO-*d*_6_) δ 174.47, 167.38, 159.41, 135.78, 113.70, 66.79, 38.13, 33.58, 28.82, 25.88, 24.20. HRMS (ESI-QTOF): calc. C_14_H_19_BNO_6_^-^ [M-H]^-^ for 308.1311, found 308.1310.

**General synthetic procedures of phenylboronic acid building blocks bearing different functional groups.** The crude **S4** (1 equiv.) was incubated with DEBPT (0.9 equiv.) and DIPEA (4 equiv.) for 5 mins in MeCN (1 ml/0.2 mmol **S4**) at rt. The amine bearing a certain functional group (1.2 equiv.) was stirred with the incubated mixture for 16 hrs. Having concentrated, the reaction mixture was diluted with DI water (4 ml/0.2 mmol **S4**) for precipitation. The precipitate was washed with DI water twice (4 ml/0.2 mmol **S4**). Complete desiccation of the precipitate yielded a phenylboronic acid building block, which was used without further purification.

**Alkyne-bearing phenylboronic acid 3c.** The crude **S4** (181 mg, 0.6 mmol), DEPBT (162 mg, 0.54 mmol), DIPEA (418 μL, 2.4 mmol), propargylamine (46 μL, 0.72 mmol), and MeCN (3 mL) were used to obtain the product via the general synthetic procedure of phenylboronic acid building blocks. Pale brown solid. Yield: 82% (103 mg). Analytical HPLC: Gradient V, retention time: 12.1 min. ^1^H NMR (400 MHz, DMSO-*d*_6_) δ 8.58 (t, *J* = 5.7 Hz, 1H), 7.91 (s, 2H), 7.73 (d, *J* = 8.6 Hz, 2H), 6.90 (d, *J* = 8.6 Hz, 2H), 4.51 (s, 2H), 3.92 (dd, *J* = 5.7, 2.5 Hz, 2H), 3.12 (t, *J* = 2.5 Hz, 1H). ^13^C NMR (101 MHz, DMSO-*d*_6_) δ 167.54, 159.32, 135.80, 113.70, 81.04, 72.90, 66.57, 27.74. HRMS (ESI-QTOF): calc. C_11_H_13_BNO_4_^+^ [M+H]^+^ for 234.0932, found 234.0938.

**Tetrazine-bearing phenylboronic acid 3d.** The crude **S4** (197 mg, 0.65 mmol), DEPBT (147 mg, 0.49 mmol), DIPEA (455 μL, 2.61 mmol), (4-(6-methyl-1,2,4,5-tetrazin-3-yl)phenyl)methanamine trifluoroacetate (103 mg, 0.33 mmol), and MeCN (3.25 mL) were used to obtain the product via the general synthetic procedure of phenylboronic acid building blocks, with additional washing with MeCN (3.25 mL × 2). Purple solid. Yield: 93% (115 mg). Analytical HPLC: Gradient X, retention time: 16.2 min. ^1^H NMR (400 MHz, DMSO-*d*_6_) δ 8.80 (t, *J* = 6.2 Hz, 1H), 8.40 (d, *J* = 8.3 Hz, 2H), 7.94 (s, 1H), 7.76 (d, *J* = 8.7 Hz, 2H), 7.52 (d, *J* = 8.4 Hz, 2H), 6.96 (d, *J* = 8.7 Hz, 2H), 4.62 (s, 2H), 4.48 (d, *J* = 6.1 Hz, 2H), 2.98 (s, 3H). ^13^C NMR (151 MHz, DMSO-*d*_6_) δ 168.09, 167.13, 163.21, 159.39, 144.15, 135.85, 130.45, 128.12, 127.43, 113.81, 66.82, 41.72, 20.87. HRMS (ESI-QTOF): calc. C_18_H_19_BN_5_O_4_^+^ [M+H]^+^ for 380.1525, found 380.1532.

**DBCO-bearing phenylboronic acid 3e.** The crude **S4** (72 mg, 0.24 mmol), DEPBT (65 mg, 0.22 mmol), DIPEA (167 μL, 0.96 mmol), DBCO-amine (80 mg, 0.29 mmol), and MeCN (1.2 mL) were used to obtain the product via the general synthetic procedure of phenylboronic acid building blocks, with additional washing with MeCN (0.6 mL × 2). Pale brown solid. Yield: 71% (70 mg). Analytical HPLC: Gradient XX, retention time: 14.9 min. ^1^H NMR (400 MHz, DMSO-*d*_6_) δ 7.90 (t, *J* = 5.8 Hz, 3H), 7.70 (d, *J* = 8.6 Hz, 2H), 7.68 – 7.23 (m, 8H), 6.82 (d, *J* = 8.6 Hz, 2H), 4.35 (s, 2H), 4.34 (dd, *J* = 562.9, 14.0 Hz, 2H), 3.32 – 2.92 (m, 2H), 2.60 – 1.75 (m, 2H). ^13^C NMR (151 MHz, DMSO) δ 170.16, 167.35, 159.18, 151.32, 148.37, 135.77, 132.42, 129.56, 129.00, 128.28, 128.09, 127.74, 126.84, 125.23, 122.44, 121.42, 114.31, 113.66, 108.07, 66.56, 54.86, 34.77, 34.14. HRMS (ESI-QTOF): calc. C_26_H_24_BN_2_O_5_^+^ [M+H]^+^ for 455.1773, found 455.1783.

**Maleimide-bearing phenylboronic acid 3f.** The crude **S4** (603 mg, 2.0 mmol), DEPBT (539 mg, 1.8 mmol), DIPEA (1.39 mL, 8.0 mmol), 1-(2-Aminoethyl)maleimide hydrochloride (424 mg, 2.4 mmol), and MeCN (10 mL) were used to obtain the product via the general synthetic procedure of phenylboronic acid building blocks, with additional washing with MeCN (10 mL × 2). White solid. Yield: 79% (452 mg). Analytical HPLC: Gradient X, retention time: 9.76 min. ^1^H NMR (400 MHz, DMSO-*d*_6_) δ 8.22 (t, *J* = 6.2 Hz, 1H), 7.88 (s, 2H), 7.72 (d, *J* = 8.7 Hz, 2H), 7.00 (s, 2H), 6.87 (d, *J* = 8.7 Hz, 2H), 4.39 (s, 2H), 3.51 (t, *J* = 5.9 Hz, 2H), 3.27 (q, *J* = 6.1 Hz, 2H). ^13^C NMR (151 MHz, DMSO) δ 171.08, 168.01, 159.34, 135.78, 134.53, 113.70, 66.64, 36.94, 36.90. HRMS (ESI-QTOF): calc. C_14_H_16_BN_2_O_6_^+^ [M+H]^+^ for 319.1096, found 319.1101.

**(*N*-Boc)Amine-bearing phenylboronic acid 3f-NHBoc.** The crude **S4** (603 mg, 2.0 mmol), DEPBT (539 mg, 1.8 mmol), DIPEA (1.39 mL, 8.0 mmol), *N*-Boc-ethylenediamine (381 μL, 2.4 mmol), and MeCN (10 mL) were used to obtain the product via the general synthetic procedure of phenylboronic acid building blocks. Pale brown solid. Yield: 68% (414 mg). Analytical HPLC: Gradient V, retention time: 17.0 min. ^1^H NMR (400 MHz, DMSO-*d*_6_) δ 8.12 (t, *J* = 5.8 Hz, 1H), 7.90 (s, 2H), 7.73 (d, *J* = 8.6 Hz, 2H), 7.00 – 6.80 (m, 3H), 4.46 (s, 2H), 3.15 (q, *J* = 6.2 Hz, 2H), 3.01 (q, *J* = 6.2 Hz, 2H), 1.37 (s, 9H). ^13^C NMR (151 MHz, DMSO) δ 167.78, 159.34, 155.76, 135.81, 113.71, 77.75, 66.71, 38.69, 28.24. HRMS (ESI-QTOF): calc. C_15_H_23_BN_2_NaO_6_^+^ [M+Na]^+^ for 361.1541, found 361.1550; calc. C_16_H_25_BN_2_NaO_6_^+^ [M-OH+OMe+Na]^+^ for 375.1698, found 375.1703.

**Synthetic procedures and characterization data of peptide-bound phenylboronic acids:**

**Phenylboronic acid of RGD conjugate 3h**. The product was produced via the general procedure of solid-phase peptide synthesis. The reaction proceeded with the crude **S4** (341 mg, 1.1 mmol), PyBOP (588 mg, 1.1 mmol), RGD-bearing resin (loading 0.565 mmol/g, 500 mg, 0.28 mmol), and DIPEA (591 μL, 3.4 mmol). White solid. Isolated yield: 56 % (101 mg). Analytical HPLC: Gradient II, retention time: 14.2 min. ^1^H NMR (600 MHz, DMSO-*d*_6_) δ 8.37 (t, *J* = 5.8 Hz, 1H), 8.22 (d, *J* = 7.8 Hz, 1H), 8.14 (d, *J* = 8.1 Hz, 1H), 7.73 (d, *J* = 8.5 Hz, 2H), 7.62 (t, *J* = 5.8 Hz, 1H), 7.52 – 6.76 (m, 8H), 4.61 – 4.52 (m, 2H), 4.49 (td, *J* = 8.0, 5.4 Hz, 1H), 4.36 (td, *J* = 8.1, 5.5 Hz, 1H), 3.75 (ddd, *J* = 52.9, 16.6, 5.7 Hz, 2H), 3.09 (q, *J* = 6.9 Hz, 2H), 2.67 (dd, *J* = 16.5, 5.5 Hz, 1H), 2.49 (dd, *J* = 17.0, 7.8 Hz, 1H), 1.75 (dt, *J* = 7.3, 4.9 Hz, 1H), 1.59 (ddt, *J* = 13.4, 8.4, 4.8 Hz, 1H), 1.55 – 1.39 (m, 2H). ^13^C NMR (151 MHz, DMSO) δ 172.63, 171.94, 171.61, 168.63, 167.86, 159.41, 156.78, 135.89, 113.70, 66.47, 52.04, 49.41, 42.06, 40.42, 36.23, 29.10, 24.99. HRMS (MALDI-TOF): calc. C_20_H_30_N_7_O_7_^+^ [M-B(OH)_2_+2H]^+^ for 480.2201, found 480.3448; calc. C_20_H_30_N_7_O_8_+ [M-B(OH)_2_+H_2_O]^+^ for 496.2150, found 496.3347; calc. C_20_H_29_BN_7_O_8_^+^ [M-OH]^+^ for 506.2165, found 506.3308.

**Phenylboronic acid of PSMA-sequence conjugate 3i.** The crude **S6** (70 mg, 0.17 mmol) was pre-incubated with DEPBT (15 mg, 0.051 mmol) and DIPEA (78 μL, 0.45 mmol) in DMF (1.7 mL) for 5 mins. Glu-urea-Lys hydrochloride (20 mg, 0.056 mmol) had been added to the reaction mixture, stirring for 16 hrs. The mixture was diluted with DI water (2 mL/0.1 mmol **S6**). It was partitioned with EA (2 mL/0.1 mmol **S6**) three times. After purification with HPLC, the mixture of **3i** and its side-product with one additional PSMA-sequence was obtained. The mixture was used without further separation. The identification and quantification of **3i** were performed in LC-DAD and LC-MS. Yield: 47% (28 mg, purity by weight: 51 %). Analytical HPLC: Gradient V, retention time: 12.1 min. MS (ESI): calc. C_26_H_38_BN_4_O_11_^+^ [M-2OH-H]^+^ for 575.3, found 575.3.

**Phenylboronic acid of GE11 conjugate 3j.** The product was produced via the general procedure of solid-phase peptide synthesis. The reaction proceeded with the crude **S4** (121 mg, 0.40 mmol), PyBOP (210 mg, 0.40 mmol), the GE11-bearing resin (loading 0.183 mmol/g, 550 mg, 0.10 mmol), and DIPEA (211 μL, 1.2 mmol). The crude product was used directly for further reaction without purification. White solid. Yield: 48% (100 mg, purity by weight: 89 %). Analytical HPLC: Gradient V, retention time: 17.2 min. MS (ESI): calc. C_83_H_106_BN_17_O_23_^2+^ [M+2H]^2+^ for 859.9, found 860.0.

**Phenylboronic acid of *c*(RGDyK) conjugate 3k.** The crude **S6** (22 mg, 0.053 mmol) was pre-incubated with PyBOP (15 mg, 0.028 mmol) and DIPEA (25 μL, 0.14 mmol) in DMF (530 μL) for 5 min. The di-trifluoroacetate salt of *c*(RGDyK) (30 mg, 0.036 mmol) was added to the reaction mixture, stirring for 16 hrs. The mixture was diluted with DI water (4 mL). It was washed with EA (4 mL × 3). The product was purified with HPLC. White solid. Isolated yield: 61% (18 mg). Analytical HPLC: Gradient V, retention time: 13.2 min. ^1^H NMR (400 MHz, DMSO-*d*_6_) δ 8.38 (dd, *J* = 7.5, 4.4 Hz, 1H), 8.17 – 8.01 (m, 3H), 7.94 (d, *J* = 7.2 Hz, 1H), 7.78 (t, *J* = 5.6 Hz, 1H), 7.73 (d, *J* = 8.5 Hz, 2H), 7.62 (q, *J* = 6.8, 5.8 Hz, 2H), 6.90 (d, *J* = 8.8 Hz, 8H), 6.63 (d, *J* = 8.5 Hz, 2H), 4.63 (td, *J* = 8.5, 5.9 Hz, 1H), 4.46 (s, 2H), 4.34 (q, *J* = 7.2 Hz, 1H), 4.16 (q, *J* = 7.5 Hz, 1H), 4.04 (dd, *J* = 15.0, 7.6 Hz, 1H), 3.91 (ddd, *J* = 11.4, 7.0, 4.6 Hz, 1H), 3.24 (dd, *J* = 15.0, 4.1 Hz, 1H), 3.09 (p, *J* = 8.8, 7.8 Hz, 4H), 2.96 (q, *J* = 6.9 Hz, 2H), 2.85 – 2.64 (m, 3H), 2.36 (dd, *J* = 16.3, 5.7 Hz, 1H), 2.04 (t, *J* = 7.5 Hz, 2H), 1.79 – 1.65 (m, 1H), 1.61 – 1.16 (m, 13H), 1.13 – 0.98 (m, 2H). ^13^C NMR (151 MHz, DMSO) δ 172.22, 172.08, 171.75, 171.22, 170.89, 169.94, 169.59, 167.44, 159.43, 156.73, 155.84, 135.81, 130.04, 127.34, 114.97, 113.73, 66.81, 54.74, 54.65, 51.86, 48.91, 43.28, 40.31, 38.24, 38.19, 36.72, 35.41, 35.05, 30.93, 28.95, 28.70, 28.53, 26.11, 25.26, 25.08, 22.92. HRMS (ESI-QTOF): calc. C_41_H_58_BN_10_O_12_^+^ [M-OH]^+^ for 893.4323, found 893.4332.

Optimisation of reaction conditions for synthesizing an α-aryl-substituted DOTA via Petasis reaction:

**Scheme S4.** The synthesis of **4a**.

With vigorous stirring, 9M glyoxylic acid **2a** (aq.) (8.3 μL, 0.075 mmol, 1.5 equiv.) was introduced to the mixture of 4-methoxyphenylboronic acid **3a** (7.6 mg, 0.050 mmol, 1.0 equiv.) and tri-sodium DO3A salt (31 mg, 0.075 mmol, 1.5 equiv.) in HFIP (500 μL). The reactions were performed under different conditions, as tabulated below. The conversions of **3a** to product **4a** and unknown side-products were monitored by an analytical HPLC (Table S7).

Table S7. The conversions of 3a to 4a and the side products of reactions under different conditions.

| Conditions | Solvent | Temp. (°C) | Duration (h) | 4a (%) | Remaining 3a (%) | Side products (%) |
| --- | --- | --- | --- | --- | --- | --- |
| (a) | HFIP | 40 | 16 | 76 | 20 | 4 |
| (b) | HFIP | 40 | 24 | 95 | 0 | 5 |
| (c) | HFIP | 60 | 16 | 93 | 0 | 7 |
| (d) | TFE | 40 | 16 | 94 | 2 | 4 |
| (e) | TFE | 40 | 24 | 95 | 0 | 5 |
| (f) | MeOH | 40 | 16 | 16 | 84 | trace |
| (g) | EtOH | 40 | 16 | 11 | 89 | trace |
| (h) | *^i^*PrOH | 40 | 16 | 1 | 98 | trace |
| (i) | DMSO | 40 | 16 | 2 | 97 | 1 |
| (j) | DMF | 40 | 16 | trace | 100 | trace |
| (k) | THF | 40 | 16 | n.d. | 28 | 72 |
| (l) | MeCN | 40 | 16 | 1 | 37 | 62 |
| (m) | DCM | 40 | 16 | n.d. | 95 | 5 |


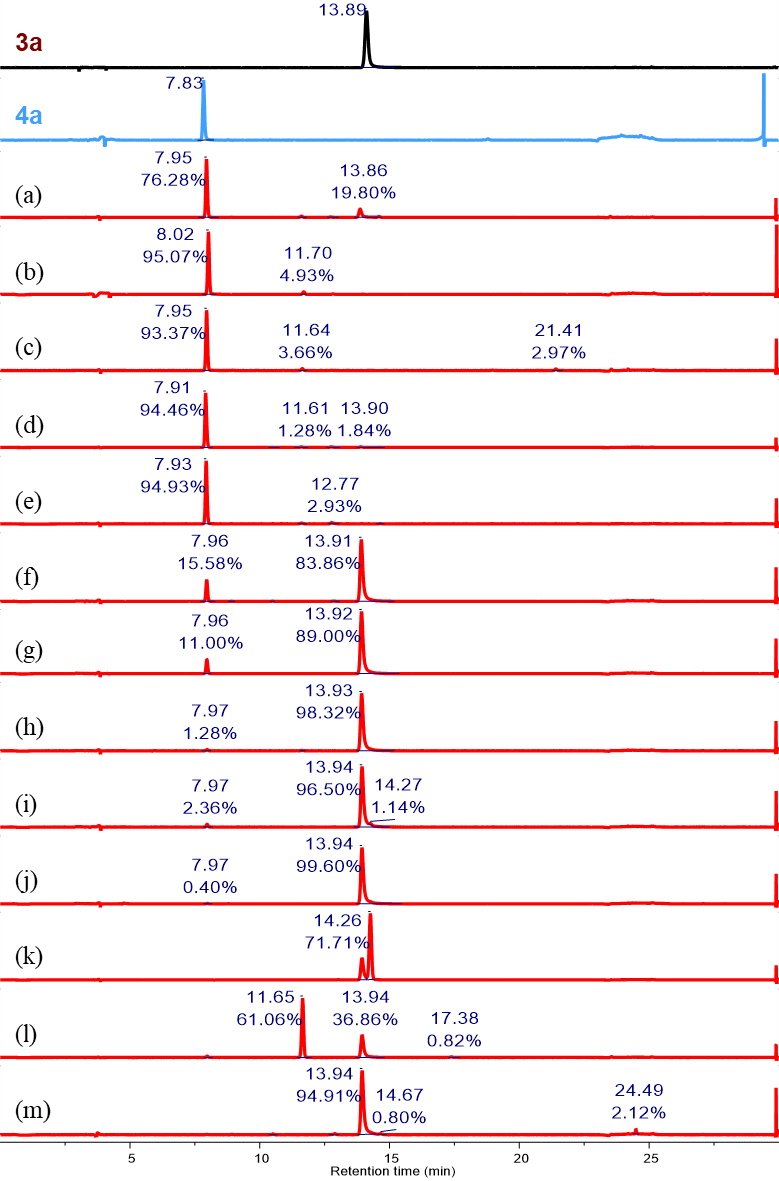


**Figure S1.** Analytical HPLC chromatograms of **3a**, **4a**, and the reaction mixture under conditions (a)-(m) **(**Gradient V, *λ*_abs_ = 280 nm)


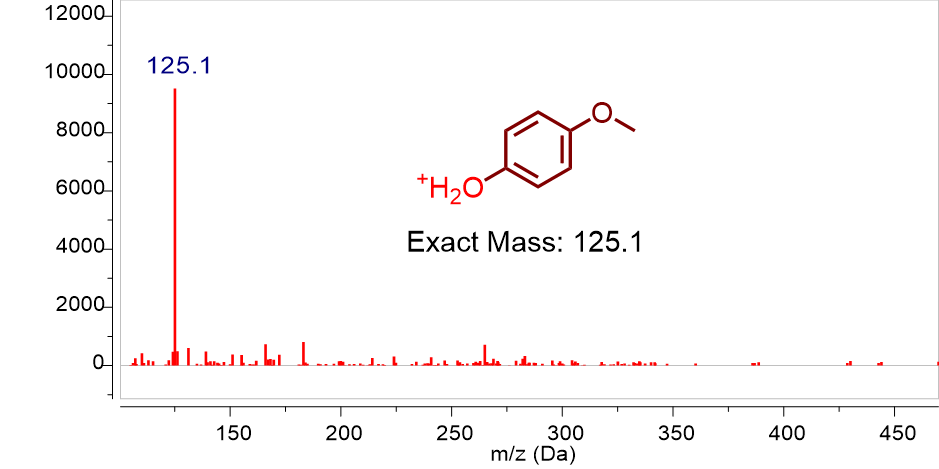


ESI-MS spectrum of the species at 14.3 min in the analytical HPLC chromatogram of the reaction mixture under condition (k), with a plausible structure of the species capable of generating the signal in MS


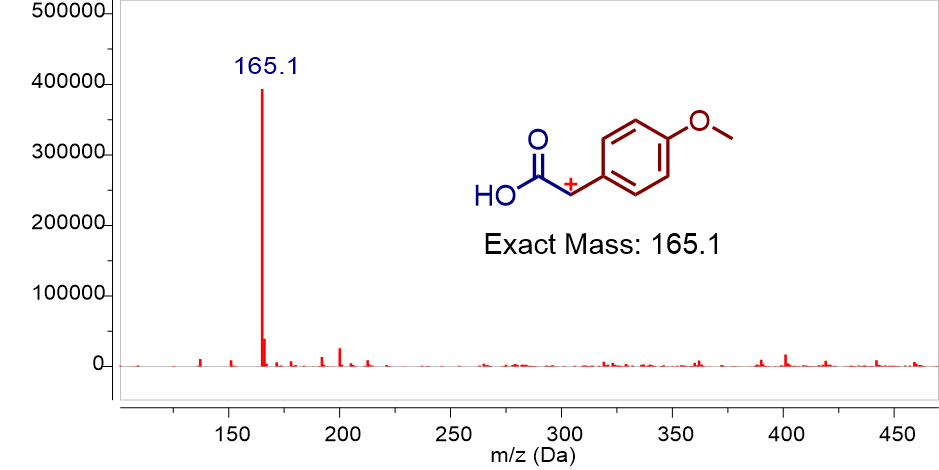


ESI-MS spectrum of the species at 11.7 min in the analytical HPLC chromatogram of the reaction mixture under condition (l), with a plausible structure of the species capable of generating the signal in MS

General synthetic procedures of α-aryl-substituted DOTA via Petasis reaction:

With vigorous stirring, 9M glyoxylic acid **2a** (aq.) (1.5 – 5.0 equiv.) was introduced to the mixture of RB(OH)_2_ (1.0 equiv.) and tri-sodium DO3A salt (1.5 – 5.0 equiv.) in HFIP (1 ml/0.1 mmol RB(OH)_2_). The reaction mixture was stirred for 24 to 48 h at 40℃. Upon the completion of the reaction, the reaction mixture was concentrated and re-diluted in DI water. In case of precipitation, the filtrate was obtained. The product was purified with a preparative HPLC. The quantification was performed by NMR with dibromomethane as an internal standard.

**α-Aryl-substituted DOTA (DOTAPMP) 4a.** The reaction proceeded with **3a** (61 mg, 0.40 mmol), tri-sodium DO3A salt (247 mg, 0.60 mmol), **2a** (66 µL, 0.60 mmol), and 4 mL HFIP for 24 h. White solid. Isolated yield: 42% (143 mg). Analytical HPLC: Gradient II, retention time: 12.9 min. ^1^H NMR (600 MHz, DMSO-*d*_6_) δ 7.34 (d, *J* = 8.1 Hz, 2H), 6.92 (d, *J* = 7.9 Hz, 2H), 5.15 (s, 1H), 3.76 (s, 3H), 4.80 – 1.55 (m, 22H). ^13^C NMR (151 MHz, DMSO) δ 173.29, 172.77, 168.39, 159.22, 131.74, 124.42, 113.87, 62.92, 55.17, 54.35, 52.85, 51.26, 49.48, 45.71, 43.58. HRMS (MALDI-TOF): calc. C_23_H_35_N_4_O_9_^+^ [M+H]^+^ for 511.2399, found 511.2977; calc. C_23_H_35_N_4_NaO_9_^+^ [M+Na]^+^ for 533.2218, found 533.2874.

**α-Aryl-substituted DOTA-azide building block 4b.** The reaction proceeded with (4-(2-azidoethoxy)phenyl)boronic acid (156 mg, 0.76 mmol), tri-sodium DO3A salt (623 mg, 1.5 mmol), **2a** (334 µL, 3.0 mmol), and 7.5 mL HFIP for 30 h. White solid. Isolated yield: 57% (391 mg). Analytical HPLC: Gradient V, retention time: 11.3 min. ^1^H NMR (400 MHz, DMSO-*d*_6_) δ 7.36 (d, *J* = 8.7 Hz, 2H), 6.95 (d, *J* = 8.5 Hz, 2H), 5.18 (s, 1H), 4.25 – 4.12 (m, 2H), 3.65 (t, *J* = 5.0 Hz, 2H), 4.92 – 1.45 (m, 22H). ^13^C NMR (151 MHz, DMSO) δ 173.23, 172.88, 168.23, 158.05, 131.91, 124.82, 114.44, 66.93, 62.52, 54.58, 52.75, 51.44, 51.00, 50.20, 49.67, 49.67, 45.79, 43.54. HRMS (ESI-QTOF): calc. C_24_H_36_N_7_O_9_^+^ [M+]^+^ for 566.2569, found 566.2568.

**α-Aryl-substituted DOTA-alkyne building block 4c.** The reaction proceeded with **3c** (15 mg, 0.065 mmol), tri-sodium DO3A salt (107 mg, 0.26 mmol), **2a** (11 µL, 0.098 mmol), and 650 µL HFIP for 36 h. White solid. Isolated yield: 34% (21 mg). Analytical HPLC: Gradient II, retention time: 11.6 min. ^1^H NMR (400 MHz, DMSO-*d*_6_) δ 8.63 (s, 1H), 7.36 (d, *J* = 8.7 Hz, 2H), 6.95 (d, *J* = 8.7 Hz, 2H), 5.12 (s, 1H), 4.53 (s, 2H), 3.93 (dd, *J* = 5.5, 2.4 Hz, 2H), 4.86 – 1.48 (m, 22H), 3.12 (s, 1H). ^13^C NMR (151 MHz, DMSO) δ 173.18, 172.90, 168.36, 167.50, 157.55, 131.69, 125.43, 114.58, 81.05, 72.96, 66.74, 62.83, 54.27, 52.89, 51.30, 45.73, 43.65, 27.76. HRMS (ESI-QTOF): calc. C_27_H_38_N_5_O_10_^+^ [M+]^+^ for 592.2613, found 592.2616.

**α-Aryl-substituted DOTA-tetrazine building block 4d.** The reaction proceeded with **3d** (14 mg, 0.037 mmol), tri-sodium DO3A salt (76 mg, 0.18 mmol), **2a** (20 µL, 0.18 mmol), and 370 µL HFIP for 42 h. Purple solid. Isolated yield: 68% (27 mg). Analytical HPLC: Gradient V, retention time: 14.3 min. ^1^H NMR (400 MHz, DMSO-*d*_6_) δ 8.86 (t, *J* = 5.8 Hz, 1H), 8.41 (d, *J* = 8.4 Hz, 2H), 7.54 (d, *J* = 8.4 Hz, 2H), 7.38 (d, *J* = 8.7 Hz, 2H), 7.00 (d, *J* = 8.7 Hz, 2H), 5.14 (s, 1H), 4.63 (s, 2H), 4.49 (d, *J* = 6.1 Hz, 2H), 4.98 – 1.80 (m, 22H), 2.99 (s, 3H). ^13^C NMR (151 MHz, DMSO) δ 173.21, 172.88, 172.76, 168.37, 168.04, 167.17, 163.25, 157.64, 144.16, 131.75, 130.48, 128.15, 127.48, 125.50, 114.67, 66.95, 62.95, 54.28, 52.94, 51.31, 45.73, 43.70, 41.74, 20.88. HRMS (ESI-QTOF): calc. C_34_H_44_N_9_O_10_^+^ [M+H]^+^ for 738.3210, found 738.3206.

**α-Aryl-substituted DOTA-DBCO building block 4e.** The reaction proceeded with **3e** (13 mg, 0.028 mmol), tri-sodium DO3A salt (45 mg, 0.11 mmol), **2a** (12 µL, 0.11 mmol), and 280 µL HFIP for 48 h. White solid. Isolated yield: 71% (23 mg). Analytical HPLC: Gradient X, retention time: 15.3 min. ^1^H NMR (400 MHz, DMSO-*d*_6_) δ 7.97 (t, *J* = 5.9 Hz, 1H), 7.63 (d, *J* = 7.6 Hz, 1H), 7.58 (dt, *J* = 5.5, 3.1 Hz, 1H), 7.53 – 7.28 (m, 7H), 7.25 (d, *J* = 7.3 Hz, 1H), 6.87 (d, *J* = 8.4 Hz, 2H), 5.12 (s, 1H), 5.04 (d, *J* = 14.0 Hz, 1H), 4.36 (s, 2H), 3.62 (d, *J* = 13.9 Hz, 1H), 4.76 – 2.03 (m, 22H), 3.33 – 3.17 (m, 1H), 3.05 (dt, *J* = 12.8, 6.4 Hz, 1H), 2.50 (dt, *J* = 14.3, 7.0 Hz, 1H), 1.90 (dt, *J* = 15.1, 7.2 Hz, 1H). ^13^C NMR (151 MHz, DMSO) δ 173.08, 172.85, 170.30, 168.55, 167.48, 157.51, 151.44, 148.48, 132.53, 131.69, 129.66, 129.13, 128.42, 128.21, 127.87, 126.97, 125.76, 125.35, 122.57, 121.54, 114.67, 114.44, 108.22, 66.82, 63.25, 54.98, 54.39, 53.20, 51.28, 50.97, 45.71, 44.06, 34.98, 34.26. HRMS (ESI-QTOF): calc. C_42_H_49_N_6_O_11_^+^ [M+H]^+^ for 813.3454, found 813.3459.

Modification procedures (if any) and characterization data of α-aryl-substituted DOTA building blocks after Petasis reaction:

**Scheme S5.** The synthetic route of α-aryl-substituted DOTA-maleimide building block **4f**.

**4f-NHBoc.** The product was prepared via general synthetic procedures of α-aryl-substituted DOTA via Petasis reaction. The reaction proceeded with **3f-NHBoc** (34 mg, 0.10 mmol), tri-sodium DO3A salt (206 mg, 0.50 mmol), **2a** (55 µL, 0.50 mmol), and 1 mL HFIP for 24 h. White solid. Analytical HPLC: Gradient V, retention time: 12.4 min. ^1^H NMR (400 MHz, DMSO-*d*_6_) δ 8.22 (t, *J* = 5.6 Hz, 1H), 7.35 (d, *J* = 8.3 Hz, 2H), 7.00 – 6.86 (m, 3H), 5.13 (s, 1H), 4.48 (s, 2H), 4.80 – 1.55 (m, 22H), 3.17 (q, *J* = 6.1 Hz, 2H), 3.02 (q, *J* = 6.1 Hz, 2H), 1.36 (s, 9H). ^13^C NMR (151 MHz, DMSO) δ 173.28, 172.87, 168.62, 168.45, 167.87, 155.91, 131.73, 125.66, 114.69, 77.85, 66.96, 63.16, 54.40, 53.06, 51.32, 50.94, 49.20, 45.82, 43.86, 39.59, 38.84, 28.33. HRMS (ESI-QTOF): calc. C_31_H_49_N_6_O_12_^+^ [M+H]^+^ for 697.3403, found 697.3411.

**4f-NH_2_.** The crude **4f-NHBoc** was treated with TFA/DCM/TIPS, v/v/v, 50/50/2.5 (2 mL) for 16 hrs. The reaction mixture was diluted with Et_2_O (20 mL). The precipitate was collected and washed with Et_2_O (20 mL × 2). Removing the solvent yielded nearly pure **4f-NH_2_**. White solid. Analytical HPLC: Gradient I, retention time: 7.7 min. ^1^H NMR (400 MHz, DMSO-*d*_6_) δ 8.45 (s, 1H), 8.01 (s, 3H), 7.35 (d, *J* = 8.8 Hz, 2H), 6.95 (d, *J* = 8.7 Hz, 2H), 5.08 (s, 1H), 4.51 (s, 2H), 3.64 (d, *J* = 308.4 Hz, 22H), 3.42 (ddt, *J* = 21.0, 14.2, 7.1 Hz, 2H), 2.93 (q, *J* = 6.1 Hz, 2H). ^13^C NMR (151 MHz, DMSO) δ 173.10, 172.85, 168.98, 168.63, 157.63, 131.58, 126.25, 114.82, 67.15, 63.64, 54.13, 53.25, 51.38, 51.02, 48.18, 45.70, 44.44, 38.64, 36.33. HRMS (ESI-QTOF): calc. C_26_H_41_N_6_O_10_^+^ [M+H]^+^ for 597.2879, found 597.2882.

**α-Aryl-substituted DOTA-maleimide building block 4f.** The mixture of **4f-NH_2_** and maleic anhydride (39 mg, 0.4 mmol) in AcOH (1 mL) was stirred for 4 hrs at 100°C. The reaction mixture was diluted with DI water before product **4f** was purified with a preparative HPLC. White solid. Isolated yield: 54% (3 steps, 55 mg). Analytical HPLC: Gradient II, retention time: 14.0 min. ^1^H NMR (400 MHz, DMSO-*d*_6_) δ 8.29 (t, *J* = 5.6 Hz, 1H), 7.36 (d, *J* = 8.7 Hz, 2H), 7.01 (s, 2H), 6.93 (d, *J* = 8.7 Hz, 2H), 5.14 (s, 1H), 4.42 (s, 2H), 3.52 (t, *J* = 5.8 Hz, 2H), 4.85 – 1.85 (m, 22H), 3.29 (q, *J* = 5.8 Hz, 2H). ^13^C NMR (151 MHz, DMSO) δ 173.29, 172.77, 171.16, 168.41, 168.06, 157.61, 134.59, 131.75, 125.44, 114.59, 66.78, 62.86, 54.32, 52.89, 51.32, 45.67, 43.61, 36.99. HRMS (ESI-QTOF): calc. C_30_H_41_N_6_O_12_^+^ [M+H]^+^ for 677.2777, found 677.2782.

**Scheme S6.** The synthetic route of α-aryl-substituted DOTA-isothiocyanate building block **4g**.

**4g-NHBoc.** The product was prepared via general synthetic procedures of α-aryl-substituted DOTA via Petasis reaction. The reaction proceeded with 4-(*N*-Boc-amino)phenylboronic acid (59 mg, 0.25 mmol), tri-sodium DO3A salt (309 mg, 0.75 mmol), **2a** (41 µL, 0.38 mmol), and 2.5 mL HFIP for 24 h. White solid. Analytical HPLC: Gradient V, retention time: 13.6 min. ^1^H NMR (400 MHz, DMSO-*d*_6_) δ 9.43 (s, 1H), 7.45 (d, *J* = 8.7 Hz, 2H), 7.25 (d, *J* = 8.8 Hz, 2H), 4.79 (s, 1H), 4.12 – 2.45 (m, 22H), 1.47 (s, 9H). ^13^C NMR (151 MHz, DMSO) δ 173.07, 172.17, 169.87, 169.73, 152.73, 139.52, 130.44, 127.58, 117.73, 79.18, 65.74, 54.19, 53.66, 51.92, 48.12, 45.59, 28.12. HRMS (ESI-QTOF): calc. C_27_H_42_N_5_O_10_^+^ [M+H]^+^ for 596.2926, found 596.2926.

**4g-NH_2_.** **4g-NHBoc** was treated with TFA/DCM/TIPS, v/v/v, 50/50/2.5 (5 mL) for 16 hrs. The reaction mixture was diluted with Et_2_O ten times the volume. The precipitate was collected and washed with Et_2_O. Removing the solvent yielded highly pure **4g-NH_2_**, which was used without further purification. The identification of **4g-NH_2_** was performed in LC-MS. White solid. MS (ESI): calc. C_22_H_34_N_5_O_8_^+^ [M+H]^+^ for 496.2, found 496.2.

**α-Aryl-substituted DOTA-isothiocyanate building block 4g.** To the mixture of crude **4g-NH_2_** (147 mg) in DI water (5 mL), 1M CSCl_2_ in CHCl_3_ (440 µL) was added. The mixture was stirred for 3 h. The mixture was partitioned between EA and DI water. Aqueous layer was collected and treated with a preparative HPLC to obtain pure **4g**. White solid. Isolated yield: 55% (3 steps, 121 mg). Analytical HPLC: Gradient V, retention time: 13.5 min. ^1^H NMR (400 MHz, DMSO-*d*_6_) δ 7.49 (d, *J* = 8.6 Hz, 2H), 7.41 (d, *J* = 8.4 Hz, 2H), 5.10 (s, 1H), 4.56 – 1.88 (m, 22H). ^13^C NMR (101 MHz, DMSO) δ 172.65, 168.77, 133.82, 131.69, 130.20, 129.86, 125.95, 63.98, 54.19, 53.36, 51.55, 51.21, 48.04, 44.99. HRMS (ESI-QTOF): calc. C_23_H_32_N_5_O_8_S^+^ [M+H]^+^ for 538.1966, found 538.1969.

**α-Aryl-substituted DOTA of RGD conjugate 4h.** The reaction proceeded with **3h** (16 mg, 0.025 mmol), tri-sodium DO3A salt (52 mg, 0.13 mmol), **2a** (14 µL, 0.13 mmol), and 250 μL HFIP for 48 h. White solid. Isolated yield: 34 % (11 mg). Analytical HPLC: Gradient II, retention time: 8.8 min. HRMS (MALDI-TOF): calc. C_23_H_32_GdN_4_O_9_^+^ [M+2H]^+^ for 666.1410, found 666.2157. ^1^H NMR (400 MHz, DMSO-*d*_6_) δ 8.37 (q, *J* = 6.0 Hz, 1H), 8.21 (d, *J* = 7.6 Hz, 1H), 8.14 (d, *J* = 8.2 Hz, 1H), 7.76 (d, *J* = 14.5 Hz, 1H), 7.35 (d, *J* = 8.2 Hz, 2H), 7.26 (s, 1H), 7.15 (s, 2H), 6.95 (d, *J* = 8.2 Hz, 2H), 5.12 (s, 1H), 4.58 (s, 2H), 4.49 (q, *J* = 7.8 Hz, 1H), 4.37 (q, *J* = 7.0 Hz, 1H), 3.75 (ddd, *J* = 37.5, 16.6, 5.6 Hz, 2H), 4.82 – 2.00 (m, 22H), 3.08 (t, *J* = 6.0 Hz, 2H), 2.67 (dd, *J* = 16.4, 5.4 Hz, 1H), 2.49 (dd, *J* = 17.6, 7.9 Hz, 1H), 1.85 – 1.31 (m, 4H). ^13^C NMR (151 MHz, DMSO) δ 172.56, 171.87, 171.55, 168.55, 167.69, 157.63, 156.86, 131.65, 126.02, 114.62, 66.66, 63.08, 54.26, 52.96, 51.99, 51.27, 50.86, 49.37, 45.64, 42.01, 40.29, 36.21, 29.18, 29.11, 25.01, 24.94. HRMS (ESI-QTOF): calc. C_36_H_56_N_11_O_15_^+^ [M+H]^+^ for 882.3952, found 882.3949.

**α-Aryl-substituted DOTA of PSMA-sequence conjugate 4i.** The reaction proceeded with **3i** (29 mg, 0.024 mmol), tri-sodium DO3A salt (49 mg, 0.12 mmol), **2a** (13 µL, 0.12 mmol), and 240 μL HFIP for 48 h. White solid. Isolated yield: 24 % (8 mg). Analytical HPLC: Gradient II, retention time: 17.5 min. ^1^H NMR (400 MHz, DMSO-*d*_6_) δ 8.09 (t, *J* = 5.9 Hz, 1H), 7.76 (t, *J* = 5.6 Hz, 1H), 7.34 (d, *J* = 8.2 Hz, 2H), 6.94 (d, *J* = 8.2 Hz, 2H), 6.35 (d, *J* = 8.2 Hz, 1H), 6.31 (d, *J* = 8.3 Hz, 1H), 5.07 (s, 1H), 4.47 (s, 2H), 4.08 (td, *J* = 8.5, 5.2 Hz, 1H), 4.03 (td, *J* = 8.2, 5.2 Hz, 1H), 4.76 – 2.37 (m, 22H), 3.10 (q, *J* = 6.8 Hz, 2H), 2.99 (q, *J* = 6.6 Hz, 2H), 2.23 (hept, *J* = 8.0, 7.1 Hz, 2H), 2.02 (t, *J* = 7.4 Hz, 2H), 1.91 (dq, *J* = 14.4, 6.9 Hz, 1H), 1.76 – 1.57 (m, 2H), 1.57 – 1.31 (m, 7H), 1.31 – 1.14 (m, 4H). ^13^C NMR (151 MHz, DMSO) δ 174.58, 174.21, 173.77, 173.13, 172.75, 171.95, 168.56, 167.35, 157.65, 157.35, 131.57, 125.80, 114.60, 66.94, 63.25, 54.12, 53.08, 52.31, 51.70, 51.42, 51.01, 45.59, 38.32, 38.27, 35.37, 31.82, 29.93, 28.96, 28.88, 27.54, 26.12, 25.08, 22.67. HRMS (ESI-QTOF): calc. C_42_H_65_N_8_O_18_^+^ [M+H]^+^ for 969.4411, found 969.4421.

**α-Aryl-substituted DOTA of GE11 conjugate 4j.** The reaction proceeded with **3j** (56 mg, 0.027 mmol), tri-sodium DO3A salt (57 mg, 0.14 mmol), **2a** (15 µL, 0.14 mmol), and 280 μL HFIP for 48 h. White solid. Isolated yield: 22 % (15 mg). Analytical HPLC: Gradient X, retention time: 12.5 min. HRMS (MALDI-TOF): calc. C_99_H_130_N_21_O_29_^+^ [M+H]^+^ for 2075.9498, found 2076.0454.

**α-Aryl-substituted DOTA of *c*(RGDyK) conjugate 4k.** The reaction proceeded with **3k** (25 mg, 0.024 mmol), tri-sodium DO3A salt (50 mg, 0.12 mmol), **2a** (13 µL, 0.12 mmol), and 240 μL HFIP for 48 h. White solid. Isolated yield: 64 % (26 mg). Analytical HPLC: Gradient V, retention time: 10.9 min. ^1^H NMR (400 MHz, DMSO-*d*_6_) δ 8.44 – 8.30 (m, 1H), 8.12 (d, *J* = 7.9 Hz, 3H), 7.93 (d, *J* = 7.2 Hz, 1H), 7.80 (t, *J* = 5.7 Hz, 1H), 7.75 (t, *J* = 5.4 Hz, 1H), 7.67 (d, *J* = 8.2 Hz, 1H), 7.36 (d, *J* = 8.7 Hz, 2H), 6.95 (d, *J* = 8.4 Hz, 2H), 6.92 (d, *J* = 8.7 Hz, 2H), 6.63 (d, *J* = 8.5 Hz, 2H), 5.15 (s, 1H), 4.63 (td, *J* = 8.5, 5.7 Hz, 1H), 4.48 (s, 2H), 4.34 (q, *J* = 7.3 Hz, 1H), 4.17 (q, *J* = 8.2 Hz, 1H), 4.03 (dd, *J* = 15.1, 7.6 Hz, 1H), 3.92 (dq, *J* = 7.4, 4.9, 3.8 Hz, 1H), 4.94 – 2.18 (m, 22H), 3.24 (dd, *J* = 15.3, 3.6 Hz, 1H), 3.10 (p, *J* = 6.8 Hz, 4H), 2.96 (q, *J* = 7.1 Hz, 2H), 2.84 – 2.63 (m, 3H), 2.35 (dd, *J* = 16.3, 5.7 Hz, 1H), 2.04 (t, *J* = 7.5 Hz, 2H), 1.81 – 1.64 (m, 1H), 1.64 – 1.13 (m, 13H), 1.06 (qd, *J* = 14.0, 13.5, 7.4 Hz, 2H). ^13^C NMR (151 MHz, DMSO) δ 172.89, 172.26, 172.13, 171.81, 171.21, 171.09, 170.87, 169.95, 169.61, 168.17, 167.39, 157.69, 156.87, 155.87, 131.75, 130.06, 127.40, 124.89, 115.00, 114.60, 66.93, 62.51, 54.78, 54.67, 54.47, 52.71, 51.83, 51.48, 51.29, 50.03, 48.92, 45.70, 43.42, 43.30, 40.32, 38.31, 38.23, 36.75, 35.43, 35.08, 30.99, 29.02, 28.67, 28.55, 26.18, 25.26, 25.13, 22.95. HRMS (ESI-QTOF): calc. C_57_H_85_N_14_O_19_^+^ [M+H]^+^ for 1269.6110, found 1269.6584.

**General procedures of metalation of α-aryl-substituted DOTA:**

The α-aryl-substituted DOTA (0.2 M) and lanthanide nitrate hydrate (1.2 equiv.) were stirred in 1M sodium acetate buffer (pH = 6) at ambient temperature for 16 hrs. The metal complex was purified with a preparative HPLC.

**α-Aryl-substituted Gd-DOTA (Gd-DOTAPMP) 4a-Gd.** The reaction proceeded with **4a** (21 mg, 0.025 mmol), Gd(NO_3_)_3_· 6H_2_O (13 mg, 0.030 mmol), and 125 µL buffer. White solid. Isolated yield: 94% (16 mg). Analytical HPLC: Gradient II, retention time: 16.7 min. HRMS (MALDI-TOF): calc. C_23_H_32_GdN_4_O_9_^+^ [M+2H]^+^ for 666.1410, found 666.2157.

**α-Aryl-substituted Gd-DOTA-Azide 4b-Gd.** The reaction proceeded with **4b** (27 mg, 0.029 mmol), Gd(NO_3_)_3_· 6H_2_O (16 mg, 0.035 mmol), and 150 µL buffer. White solid. Isolated yield: 91% (19 mg). Analytical HPLC: Gradient V, retention time: 12.3 min. HRMS (ESI-QTOF): calc. C_24_H_33_GdN_7_O_9_^+^ [M+2H]^+^ for 721.1581, found 721.1588.

**α-Aryl-substituted Gd-DOTA-Tetrazine 4d-Gd.** The reaction proceeded with **4d** (26 mg, 0.025 mmol), Gd(NO_3_)_3_· 6H_2_O (13 mg, 0.029 mmol), and 125 µL buffer. Purple solid. Isolated yield: 92% (20 mg). Analytical HPLC: Gradient V, retention time: 14.6 min. HRMS (ESI-QTOF): calc. C_34_H_41_GdN_9_O_10_^+^ [M+2H]^+^ for 893.2220, found 893.2214.

**α-Aryl-substituted Gd-DOTA-Maleimide 4f-Gd.** The reaction proceeded with **4f** (19 mg, 0.019 mmol), Gd(NO_3_)_3_· 6H_2_O (10 mg, 0.023 mmol), and 100 µL buffer. White solid (14 mg). Isolated yield: 89%. Analytical HPLC: Gradient V, retention time: 9.4 min. HRMS (ESI-QTOF): calc. C_30_H_38_GdN_6_O_12_^+^ [M+2H]^+^ for 832.1791, found 832.1787.

**α-Aryl-substituted Gd-DOTA-NCS 4g-Gd.** The reaction proceeded with **4g** (40 mg, 0.045 mmol), Gd(NO_3_)_3_· 6H_2_O (24 mg, 0.054 mmol), and 225 µL buffer. White solid. Isolated yield: 88% (27 mg). Analytical HPLC: Gradient V, retention time: 14.5 min. HRMS (ESI-QTOF): calc. C_23_H_29_GdN_5_O_8_S^+^ [M+2H]^+^ for 693.0976, found 693.0975.

**α-Aryl-substituted Gd-DOTA of RGD conjugate 4h-Gd.** The reaction proceeded with **4h** (6 mg, 0.0045 mmol), Gd(NO_3_)_3_· 6H_2_O (2 mg, 0.0054 mmol), and 25 µL buffer. White solid. Isolated yield: 97% (5 mg). Analytical HPLC: Gradient II, retention time: 12.4 min. HRMS (MALDI-TOF): calc. C_36_H_53_GdN_11_O_15_^+^ [M+2H]^+^ for 1037.2967, found 1037.3495.

**α-Aryl-substituted Gd-DOTA of PSMA-sequence conjugate 4i-Gd.** The reaction proceeded with **4i** (8 mg, 0.0063 mmol), Gd(NO_3_)_3_· 6H_2_O (3 mg, 0.0076 mmol), and 35 µL buffer. White solid. Isolated yield: 94% (7 mg). Analytical HPLC: Gradient II, retention time: 19.0 min. HRMS (ESI-QTOF): calc. C_42_H_62_GdN_8_O_18_^+^ [M+2H]^+^ for 1124.3428, found 1124.3425.

**α-Aryl-substituted DOTA of GE11 conjugate 4j-Gd.** The reaction proceeded with **4j** (14 mg, 0.0055 mmol), Gd(NO_3_)_3_· 6H_2_O (3 mg, 0.0066 mmol), and 30 µL buffer. White solid. Isolated yield: 92 % (12 mg). Analytical HPLC: Gradient V, retention time: 15.5 min. HRMS (MALDI-TOF): calc. C_99_H_126_GdN_22_Na_2_O_28_^+^ [M+2Na]^+^ for 2274.8166, found 2274.9376.

**α-Aryl-substituted DOTA of *c*(RGDyK) conjugate 4k-Gd.** The reaction proceeded with **4k** (14 mg, 0.0079 mmol), Gd(NO_3_)_3_· 6H_2_O (4 mg, 0.0095 mmol), and 40 µL buffer. White solid. Isolated yield: 93 % (11 mg). Analytical HPLC: Gradient V, retention time: 11.5 min. HRMS (ESI-QTOF): calc. C_57_H_82_GdN_14_O_19_^+^ [M+2H]^+^ for 1424.5130, found 1424.5131.

**Synthesis of analogous DO3A conjugate and its Gd chelate for comparison with 4a in terms of coordination stability and coordination kinetics:**

**DO3A-Ac-Tyr-CONH_2_ 5.** Fmoc-Tyr(Me)-OH (785 mg, 1.9 mmol) was incorporated onto the Rink AM resin (0.63 mmol/g, 300 mg, 0.19 mmol), followed by Fmoc deprotection via general procedures of solid-phase peptide synthesis. The resin was shaken with the solution of bromoacetic acid (261 mg, 1.9 mmol) and *N*,*N*'-diisopropylcarbodiimide (294 µL, 1.9 mmol) in DMF (8 mL) for an hour. The resin was washed as in the general procedures. The resin, tris-*^t^*Bu-DO3A (484 mg, 0.94 mmol), and DIEPA (328 µL, 1.9 mmol) were shaken in DMF (8 mL) for 16 hours. The resin was washed and then subjected to global cleavage with TFA/TIPS/H_2_O, v/v/v, 95/2.5/2.5 (3 mL) as in the general procedures. **5** was purified by preparative HPLC. White solid. Isolated yield: 26 % (51 mg). Analytical HPLC: Gradient II, retention time: 18.1 min. ^1^H NMR (400 MHz, DMSO-*d*_6_) δ 8.78 (d, *J* = 8.9 Hz, 1H), 7.42 (d, *J* = 192.7 Hz, 2H), 7.15 (d, *J* = 8.7 Hz, 2H), 6.82 (d, *J* = 8.7 Hz, 2H), 4.52 (td, *J* = 9.5, 4.6 Hz, 1H), 4.23 – 3.89 (m, 3H), 3.76 (d, *J* = 14.9 Hz, 1H), 3.71 (s, 3H), 3.66 – 2.74 (m, 20H), 3.03 (dd, *J* = 14.2, 4.2 Hz, 1H), 2.67 (dd, *J* = 13.7, 9.9 Hz, 1H). ^13^C NMR (151 MHz, DMSO) δ 172.38, 171.82, 168.73, 164.89, 157.92, 130.44, 129.58, 113.58, 55.02, 54.65, 54.24, 54.01, 52.67, 50.50, 48.71, 48.55, 47.78, 37.33. HRMS (ESI-QTOF): calc. C_26_H_41_N_6_O_9_^+^ [M+H]^+^ for 581.2930, found 581.2935.

**Gd-DO3A-Ac-Tyr-CONH_2_ 5-Gd.** The ligand **5** (51 mg, 0.049 mmol) and gadolinium nitrate hydrate (27 mg, 0.059 mmol) were stirred in 1M sodium acetate buffer (250 µL, pH = 6) at ambient temperature for 16 hrs. The metal complex was purified with a preparative HPLC. White solid. Isolated yield: 81 % (29 mg). Analytical HPLC: Gradient II, retention time: 16.6 min. HRMS (ESI-QTOF): calc. C_26_H_38_GdN_6_O_9_^+^ [M+H]^+^ for 736.1942, found 736.1931.

**Synthetic procedures and characterization data of luminescent α-aryl-substituted DO3A via Petasis reaction:**

**Luminescent α-aryl-substituted DO3A (DO3AAMCS124^CF3^PMP) 6a.** With vigorous stirring, **2b** (44 mg, 0.10 mmol) was introduced to the mixture of **3a** (18 mg, 0.12 mmol) and tri-sodium DO3A salt (124 mg, 0.30 mmol) in HFIP (1 mL). The reaction mixture was stirred for 24 h at 40℃. White solid. Isolated yield: 41 % (44 mg). Analytical HPLC: Gradient V, retention time: 16.0 min. ^1^H NMR (600 MHz, DMSO-*d*_6_) δ 12.26 (s, 1H), 10.59 (s, 1H), 7.73 (s, 1H), 7.64 (dd, *J* = 9.1, 2.1 Hz, 1H), 7.58 (d, *J* = 8.8 Hz, 1H), 7.39 (d, *J* = 8.8 Hz, 2H), 6.93 (d, *J* = 8.8 Hz, 2H), 6.83 (s, 1H), 5.05 (s, 1H), 3.75 (s, 3H), 4.60 – 2.19 (m, 22H). ^13^C NMR (101 MHz, DMSO-*d*_6_) δ 172.52, 170.59, 169.04, 160.56, 159.46, 141.63, 140.68, 136.43 (q, *J* = 31.6 Hz), 131.49, 125.89, 125.03, 122.66 (q, *J* = 274.8 Hz), 119.66, 115.39, 114.15, 109.21, 105.47, 65.22, 55.18, 53.67, 51.45, 48.19, 45.54. HRMS (ESI-QTOF): calc. C_33_H_40_F_3_N_6_O_9_^+^ [M+H]^+^ for 721.2803, found 721.2811.

**Luminescent α-aryl-substituted DO3A-azide 6b.** With vigorous stirring, **2b** (89 mg, 0.20 mmol) was introduced to the mixture of (4-(2-azidoethoxy)phenyl)boronic acid (50 mg, 0.24 mmol) and tri-sodium DO3A salt (247 mg, 0.60 mmol) in HFIP (2 mL). The reaction mixture was stirred for 48 h at 40℃. White solid. Isolated yield: 34 % (76 mg). Analytical HPLC: Gradient X, retention time: 14.6 min. ^1^H NMR (400 MHz, DMSO-*d*_6_) δ 12.24 (s, 1H), 10.61 (s, 1H), 7.81 (s, 1H), 7.64 (dd, *J* = 9.1, 2.1 Hz, 1H), 7.54 (d, *J* = 8.8 Hz, 1H), 7.41 (d, *J* = 8.8 Hz, 2H), 6.97 (d, *J* = 8.8 Hz, 2H), 6.82 (s, 1H), 5.01 (s, 1H), 4.17 (t, *J* = 5.0 Hz, 2H), 3.64 (t, *J* = 5.0 Hz, 2H), 4.46 – 2.53 (m, 22H). ^13^C NMR (151 MHz, DMSO-*d*_6_) δ 172.48, 170.75, 169.33, 160.54, 158.17, 141.58, 140.69, 136.40 (q, *J* = 29.9 Hz), 131.51, 126.67, 125.04, 122.65 (q, *J* = 275.5 Hz), 119.64, 115.32, 114.68, 109.18, 105.41, 66.91, 65.55, 53.80, 51.58, 49.64, 48.17, 46.02. HRMS (ESI-QTOF): calc. C_34_H_41_F_3_N_9_O_9_^+^ [M+H]^+^ for 776.2974, found 776.2975.

**General procedures of metalation of luminescent α-aryl-substituted DO3A:**

The luminescent α-aryl-substituted DO3A (0.1 M) and lanthanide nitrate hydrate (3 equiv.) were stirred in 1M sodium acetate buffer (pH = 6) at 45℃ for 16 hrs. The metal complex was purified with a preparative HPLC.

**6a-Nd.** The reaction proceeded with **6a** (8 mg, 0.0075 mmol), Nd(NO_3_)_3_· 6H_2_O (10 mg, 0.023 mmol), and 75 µL buffer. White solid. Isolated yield: 93% (6.0 mg). Analytical HPLC: Gradient X, retention time: 13.7 min. HRMS (ESI-QTOF): calc. C_33_H_36_F_3_N_6_NdO_9_Na^+^ [M+Na]^+^ for 884.1496, found 884.1500.

**6a-Sm.** The reaction proceeded with **6a** (8 mg, 0.0075 mmol), Sm(NO_3_)_3_· 6H_2_O (10 mg, 0.023 mmol), and 75 µL buffer. White solid. Isolated yield: 91% (5.9 mg). Analytical HPLC: Gradient X, retention time: 13.7 min. HRMS (ESI-QTOF): calc. C_33_H_36_F_3_N_6_SmO_9_Na^+^ [M+Na]^+^ for 892.1587, found 892.1590.

**6a-Eu.** The reaction proceeded with **6a** (8 mg, 0.0075 mmol), Eu(NO_3_)_3_· 6H_2_O (10 mg, 0.023 mmol), and 75 µL buffer. White solid. Isolated yield: 92% (6.0 mg). Analytical HPLC: Gradient X, retention time: 13.6 min. HRMS (ESI-QTOF): calc. C_33_H_36_F_3_N_6_EuO_9_Na ^+^ [M+Na]^+^ for 893.1604, found 893.1605.

**6a-Tb.** The reaction proceeded with **6a** (8 mg, 0.0075 mmol), Tb(NO_3_)_3_· 6H_2_O (10 mg, 0.023 mmol), and 75 µL buffer. White solid. Isolated yield: 94% (6.2 mg). Analytical HPLC: Gradient X, retention time: 13.5 min. HRMS (ESI-QTOF): calc. C_33_H_36_F_3_N_6_TbO_9_Na ^+^ [M+Na]^+^ for 899.1642, found 899.1640.

**6a-Yb.** The reaction proceeded with **6a** (8 mg, 0.0075 mmol), Yb(NO_3_)_3_· 5H_2_O (10 mg, 0.023 mmol), and 75 µL buffer. White solid. Isolated yield: 90% (6.0 mg). Analytical HPLC: Gradient X, retention time: 13.4 min. HRMS (ESI-QTOF): calc. C_33_H_36_F_3_N_6_YbO_9_Na ^+^ [M+Na]^+^ for 914.1782, found 914.1779.

**6b-Eu.** The reaction proceeded with **6b** (34 mg, 0.030 mmol), Eu(NO_3_)_3_· 6H_2_O (40 mg, 0.090 mmol), and 300 µL buffer. White solid. Isolated yield: 98% (27 mg). Analytical HPLC: Gradient X, retention time: 15.2 min. HRMS (ESI-QTOF): calc. C_34_H_38_EuF_3_N_9_O_9_^+^ [M+H]^+^ for 926.1955, found 926.1949.

**6b-Tb.** The reaction proceeded with **6b** (34 mg, 0.030 mmol), Tb(NO_3_)_3_· 6H_2_O (41 mg, 0.090 mmol), and 300 µL buffer. White solid. Isolated yield: 99% (28 mg). Analytical HPLC: Gradient X, retention time: 15.1 min. HRMS (ESI-QTOF): calc. C_34_H_38_TbF_3_N_9_O_9_^+^ [M+H]^+^ for 932.1993, found 932.1996.

**Synthesis of Ln-DO3A with CS124^CF3^ for comparison with 6a-Ln:**

**Scheme S7.** The synthesis of DO3AAMCS124^CF3^ **7**.

**DO3AAMCS124^CF3^ 7. S7** was synthesized according to the previous literature.^3^ The solution of tris-*^t^*Bu-DO3A (15 mg, 0.030 mmol), **S7** (23 mg, 0.075 mmol), and DIPEA (13 µL, 0.075 mmol) was stirred in DMF (600 µL) at 100°C for 3 hours. The reaction was diluted with ethyl acetate (1.2 ml). Three partitions with H_2_O of the same volume removed most of the DMF from the organic layer. Having been desiccated by Na_2_SO_4_, the organic layer was concentrated and completely dried in vacuo. The crude intermediate was treated with TFA/TIPS/H_2_O, v/v/v, 95/2.5/2.5 (1.2 ml) for 3 hours at 40℃. The reaction mixture was diluted with Et_2_O ten times the volume for precipitation of **7**. The precipitate was collected and washed with Et_2_O (1 ml × 2). Drying the precipitate resulted in crude **7**, which was used without further purification. LC/MS: Gradient V (FA instead of TFA), retention time: 5.3 min. MS (ESI): calc. C_26_H_34_F_3_N_6_O_8_^+^ [M+H]^+^ for 615.2, found 615.3.

**Metalation of 7.** The crude **7** (approx. 0.006 mmol) and lanthanide nitrate hydrate (0.018 mmol) were stirred in 1M sodium acetate buffer (60 µL, pH = 6) at 45℃ for 16 hrs. The metal complex was purified with a preparative HPLC.

**7-Nd.** White solid. Isolated yield: 48% (2.2 mg, 3 steps). Analytical HPLC: Gradient V, retention time: 12.2 min. HRMS (ESI-QTOF): calc. C_26_H_31_F_3_N_6_NdO_8_^+^ [M+H]^+^ for 756.1256, found 756.1255.

**7-Sm.** White solid. Isolated yield: 46% (2.1 mg, 3 steps). Analytical HPLC: Gradient V, retention time: 12.2 min. HRMS (ESI-QTOF): calc. C_26_H_31_F_3_N_6_SmO_8_^+^ [M+H]^+^ for 764.1348, found 764.1347.

**7-Eu.** White solid. Isolated yield: 49% (2.2 mg, 3 steps). Analytical HPLC: Gradient V, retention time: 12.2 min. HRMS (ESI-QTOF): calc. C_26_H_31_F_3_N_6_EuO_8_^+^ [M+H]^+^ for 765.1364, found 765.1359.

**7-Tb.** White solid. Isolated yield: 53% (2.5 mg, 3 steps). Analytical HPLC: Gradient V, retention time: 12.2 min. HRMS (ESI-QTOF): calc. C_26_H_31_F_3_N_6_TbO_8_^+^ [M+H]^+^ for 771.1403, found 771.1404.

**7-Yb.** White solid. Isolated yield: 51% (2.4 mg, 3 steps). Analytical HPLC: Gradient V, retention time: 12.1 min. HRMS (ESI-QTOF): calc. C_26_H_31_F_3_N_6_YbO_8_^+^ [M+H]^+^ for 786.1543, found 786.1543.

**Synthetic procedures of bio-conjugated Ln complexes:**

**Scheme S8.** The synthesis of Gd-DOTA-SSTR **8**.

**Linear peptide S8. S8** was prepared using the general procedures of solid-phase peptide synthesis, starting with fCFwKTCT-bearing resin (loading 0.213 mmol/g, 541 mg, 0.12 mmol), Fmoc-Pra-OH (154 mg, 0.46 mmol), PyBOP (239 mg, 0.46 mmol), and DIPEA (160 μL, 0.92 mmol). **S8** was purified using preparative HPLC. White solid. Isolated yield: 60% (94 mg). Analytical HPLC: Gradient V, retention time: 18.5 min. HRMS (ESI-QTOF): calc. C_54_H_73_N_12_O_11_S_2_^+^ [M+H]^+^ for 1129.4958, found 1129.4962.

**Cyclic peptide S9.** The linear peptide **S8** (1.5 mg, 0.0011 mmol) and DMSO (100 µl) were stirred in a 10 mM ammonium bicarbonate aqueous solution (20 ml) for 3 days in open air. The reaction mixture was concentrated to 5 ml before purification with preparative HPLC. White solid. Isolated yield: 70% (1.1 mg). Analytical HPLC: Gradient V, retention time: 17.5 min. HRMS (ESI-QTOF): calc. C_54_H_71_N_12_O_11_S_2_^+^ [M+H]^+^ for 1127.4801, found 1127.4820.

**Gd-DOTA-SSTR 8.** Having been purged with N_2_, the solution containing **4b-Gd** (1.0 mg, 0.0014 mmol), **S9** (1.1 mg, 0.00077 mmol), Cu(CH_3_CN)_4_PF_6_ (1.0 mg, 0.0027 mmol), TBTA (1.0 mg, 0.0019 mmol), and DIPEA (1.0 μL, 0.0057 mmol) was stirred in DMF (0.5 ml) at rt for 16 hours. The reaction mixture was diluted with 0.1% TFA (aq) before purification with preparative HPLC. White solid. Isolated yield: 42% (0.7 mg). Analytical HPLC: Gradient V, retention time: 15.9 min. HRMS (ESI-QTOF): calc. C_78_H_104_GdN_19_O_20_S_2_^2+^ [M+2H]^2+^ for 924.3195, found 924.3199.

**Scheme S9.** The synthesis of luminescent Tb-DO3A-NLS **9**.

**Luminescent Tb-DO3A-NLS 9.** Having been purged with N_2_, the solution containing **6b-Tb** (6.0 mg, 0.006 mmol), **S10** (8.9 mg, 0.005 mmol), Cu(CH_3_CN)_4_PF_6_ (2.4 mg, 0.006 mmol), TBTA (3.4 mg, 0.006 mmol), and DIPEA (7.9 μl, 0.045 mmol) was stirred in DMF (1 ml) at rt for 16 hours. The reaction mixture was diluted with 0.1% TFA in water (3 ml) before purification with preparative HPLC. White solid. Isolated yield: 43% (7.4 mg). Analytical HPLC: Gradient V, retention time: 11.0 min. HRMS (ESI-QTOF): calc. C_85_H_134_F_3_N_26_O_18_Tb^2+^ [M+2H]^2+^ for 1011.4782, found 1011.4781.

**Scheme S20.** The synthesis of luminescent Eu-DO3A-Taxol **10**.

**Alkyne-bearing Taxol S11.** A solution of Docetaxel (100 mg, 0.124 mmol) in 4 mL formic acid was shaken at rt. The Boc protecting group was completely removed after 30 min (confirmed by LC-MS). The excess formic acid was removed by airflow, and the residue was put under vacuum overnight. Then, a solution of hept-6-ynoic acid (25.2 mg, 0.2 mmol), HATU (76.0 mg, 0.2 mmol), and DIPEA (34.9 μl, 0.4 mmol) in 1 mL DMF was added to the dried residue, and shaken at rt for 2 h. The reaction mixture was diluted with 0.1% TFA (aq) before purification with preparative HPLC. White solid. Isolated yield: 44 % (45 mg). Analytical HPLC: Gradient XX, retention time: 15.1 min. ^1^H NMR (400 MHz, DMSO-*d*_6_) δ 8.43 (d, *J* = 9.0 Hz, 1H), 7.98 (d, *J* = 7.1 Hz, 2H), 7.70 (t, *J* = 7.4 Hz, 1H), 7.61 (t, *J* = 7.6 Hz, 2H), 7.37 (t, *J* = 7.5 Hz, 2H), 7.31 (d, *J* = 7.4 Hz, 2H), 7.21 (t, *J* = 7.2 Hz, 1H), 5.90 (t, *J* = 9.0 Hz, 1H), 5.41 (d, *J* = 7.2 Hz, 1H), 5.26 (dd, *J* = 9.0, 5.9 Hz, 1H), 5.09 (s, 1H), 4.91 (dd, *J* = 9.6, 2.2 Hz, 1H), 4.60 (s, 1H), 4.40 (d, *J* = 6.0 Hz, 1H), 4.08 – 3.99 (m, 3H), 3.68 (d, *J* = 7.2 Hz, 1H), 2.74 (t, *J* = 2.6 Hz, 1H), 2.31 – 2.25 (m, 1H), 2.24 (s, 3H), 2.18 (t, *J* = 7.3 Hz, 2H), 2.11 (td, *J* = 7.3, 2.6 Hz, 2H), 1.96 (dd, *J* = 15.3, 9.2 Hz, 1H), 1.84 – 1.77 (m, 1H), 1.74 (s, 3H), 1.66 (t, *J* = 12.7 Hz, 1H), 1.60 – 1.54 (m, 2H), 1.52 (s, 3H), 1.43 – 1.36 (m, 2H), 1.02 (s, 3H), 0.98 (s, 3H). ^13^C NMR (101 MHz, DMSO) δ 209.39, 172.70, 171.71, 169.76, 165.30, 139.64, 136.81, 135.96, 133.39, 130.07, 129.62, 128.73, 128.22, 127.19, 84.37, 83.76, 80.34, 76.96, 75.49, 74.84, 73.81, 73.62, 71.24, 70.84, 70.06, 57.02, 55.08, 46.00, 42.97, 36.51, 35.07, 34.79, 27.49, 26.57, 24.50, 22.44, 20.86, 17.45, 13.71, 9.84. HRMS (ESI-QTOF): calc. C_45_H_53_NNaO_13_^+^ [M+H]^+^ for 838.3409, found 838.3417.

**Luminescent Eu-DO3A-Taxol 10.** Having been purged with N_2_, the solution containing **6b-Eu** (6.0 mg, 0.006 mmol), **S11** (5.0 mg, 0.006 mmol), Cu(CH_3_CN)_4_PF_6_ (2.4 mg, 0.006 mmol), TBTA (3.4 mg, 0.006 mmol), and DIPEA (7.9 μl, 0.045 mmol) was stirred in DMF (1 ml) at rt for 16 hours. The reaction mixture was diluted with 0.1% TFA in water (3 ml) before purification with preparative HPLC. White solid. Isolated yield: 74% (8.3 mg). Analytical HPLC: Gradient X, retention time: 17.4 min. HRMS (ESI-QTOF): calc. C_79_H_92_EuF_3_N_10_O_22_^2+^ [M+2H]^2+^ for 871.2777, found 871.2776.

**Scheme S31.** The synthesis of luminescent Tb-DO3A-*c*(RGDyK) **11**.

**Alkyne-bearing *c*(RGDyK) S12.** 6-Heptynoic acid (11 μl, 0.083 mmol) was pre-incubated with PyBOP (23.1 mg, 0.044 mmol) and DIPEA (39 μl, 0.22 mmol) in DMF (1 ml) for 5 min. *c*(RGDyK) di-trifluoroacetate (47 mg, 0.055 mmol) was added to the reaction mixture, stirring for 16 hrs. The reaction mixture was diluted with 0.1% TFA in water (3 ml) before purification with preparative HPLC. White solid. Isolated yield: 47% (18 mg). Analytical HPLC: Gradient V, retention time: 13.7 min. HRMS (ESI-QTOF): calc. C_34_H_50_N_9_O_9_^+^ [M+H]^+^ for 728.3726, found 728.3729.

**Luminescent Tb-DO3A-*c*(RGDyK) 11.** Having been purged with N_2_, the solution containing **6b-Tb** (7.6 mg, 0.0082 mmol), **S12** (8.2 mg, 0.0098 mmol), Cu(CH_3_CN)_4_PF_6_ (3.1 mg, 0.0082 mmol), TBTA (4.3 mg, 0.0082 mmol), and DIPEA (5.7 μl, 0.033 mmol) was stirred in DMF (1 ml) at rt for 16 hours. The reaction mixture was diluted with 0.1% TFA in water (3 ml) before purification with preparative HPLC. White solid. Isolated yield: 93% (14 mg). Analytical HPLC: Gradient V, retention time: 14.3 min. HRMS (ESI-QTOF): calc. C_68_H_87_F_3_N_18_O_18_Tb^2+^ [M+2H]^2+^ for 830.2860, found 830.2863.

**Scheme S42.** The synthesis of luminescent Eu-DO3A-P_19_ **12**.

**Luminescent Eu-DO3A-P_19_ 12.** Having been purged with N_2_, the solution containing **6b-Eu** (11.5 mg, 0.012 mmol), **Pra-P_19_** (52.8 mg, 0.011 mmol), Cu(CH_3_CN)_4_PF_6_ (4.6 mg, 0.012 mmol), TBTA (6.6 mg, 0.012 mmol), and DIPEA (11 μl, 0.062 mmol) was stirred in DMF (1 ml) at rt for 16 hours. The reaction mixture was diluted with 0.1% TFA (aq) before purification with preparative HPLC. White solid. Isolated yield: 44% (28 mg). Analytical HPLC: Gradient X, retention time: 13.9 min. HRMS (ESI-QTOF): calc. C_199_H_309_EuF_3_N_54_O_43_S^3+^ [M+3H]^3+^ for 1462.0856, found 1462.0875.

**Scheme S53.** The synthesis of Gd-DOTA-L_2_P_4_ **13**.

**Gd-DOTA-L_2_P_4_ 13.** The solution containing **4f-Gd** (3.8 mg, 0.0046 mmol) and **L_2_P_4_** (11.5 mg, 0.0046 mmol) in 200 mM phosphate buffer solution (pH 6.8, 1 ml) was stirred for 16 hours at rt. The reaction mixture was diluted with 0.1% TFA in water (3 ml) before purification with preparative HPLC. Brick red solid. Isolated yield: 60% (9 mg). Analytical HPLC: Gradient X, retention time: 13.7 min. HRMS (ESI-QTOF): calc. C_123_H_176_GdN_31_O_28_S_2_^2+^ [M+2H]^2+^ for 1378.5998, found 1378.6019.

# Coordination stability, kinetics, and magnetic resonance experiments

Procedures for the coordination stability test of metal complexes

The coordination stability of **4a-Gd** and DOTAREM^®^ was tested with LC-DAD. The time count started as a complex for testing (1 mM) was dissolved in 0.5 M HCl in a 2 ml HPLC vial. The solution was automatically injected for HPLC analysis at certain time intervals. The robustness of the metal complex can be inferred by monitoring the change in peak ratio of the metal complex to its corresponding ligand (i.e., dissociated complex) throughout a certain period of time.

Procedures for the coordination kinetic test of ligands

The coordination kinetics of **4a** and **5** were tested with LC-DAD. The ligand for testing (50 μM) was dissolved in 0.5 M acetate buffer (pH 5.6) in a 2 ml HPLC vial. After the solution of 10-fold metal chloride was added to the vial, the time count started, and the vial was vigorously shaken for a few seconds. The well-mixed solution was automatically injected for HPLC analysis at certain time intervals. The coordination kinetics can be inferred by monitoring the change in peak ratio of the ligand to its corresponding metal complex throughout a certain period of time.

Procedures for determining *T*_1_ relaxivity of Gd chelates

Gd chelates housing in NMR borosilicate tubes (5 mm, SynthwareTM) were measured in DI water on a 1.41 Tesla (60 MHz) NMR instrument SpinsolveTM 60 from Magritek at 293K. Triplicates were performed to obtain the data.

# Photophysical experiments

Photophysical properties of **6a-Ln** and **7-Ln** were measured in 10 mM HEPES aqueous buffer, unless specifically indicated. The ultraviolet-visible (UV-Vis) absorption spectra were measured in the range of 200-800 nm using an Agilent Technologies Cary 60 UV-Vis spectrophotometer. The emission spectra at room temperature were recorded by an Edinburgh FLS1000 spectrometer, using a 450 W xenon lamp as the light source and PMT-900 detector and PMT-1700 detector for visible and NIR range, respectively. The decay measurements were performed with a µF2 pulsed lamp. The quantum yield was measured by QYPro integrating sphere from Edinburgh Instruments.


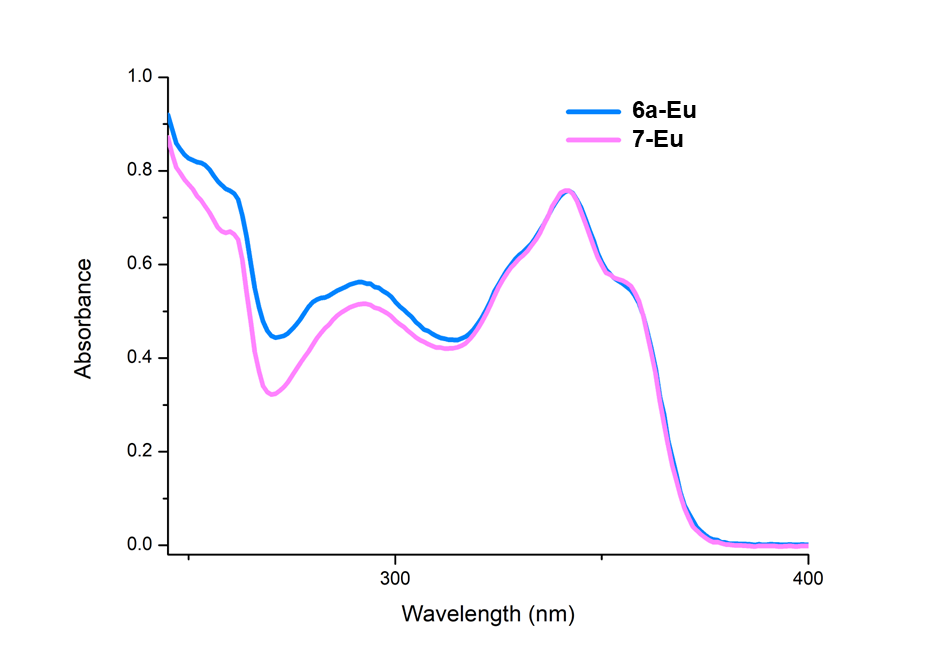


**Figure S2.** Absorption spectra of **6a-Eu** and **7-Eu** of 50 µM.


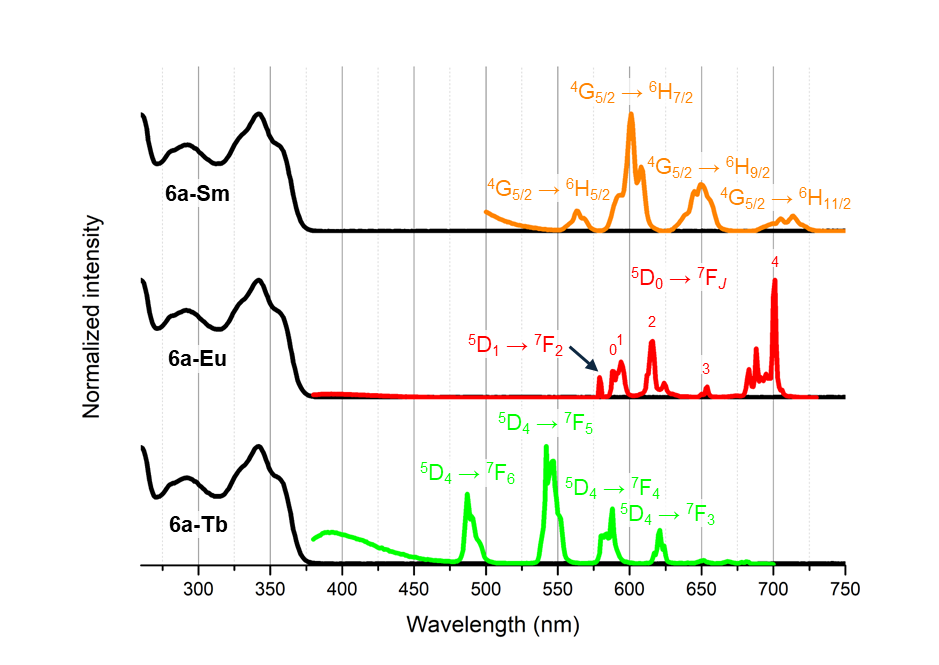


**Figure S3.** Normalized absorption spectra (in black lines) and normalized emission spectra (in coloured lines) in the visible region of **6a-Sm**, **6a-Eu**, and **6a-Tb** (*λ*_ex_ = 342 nm).


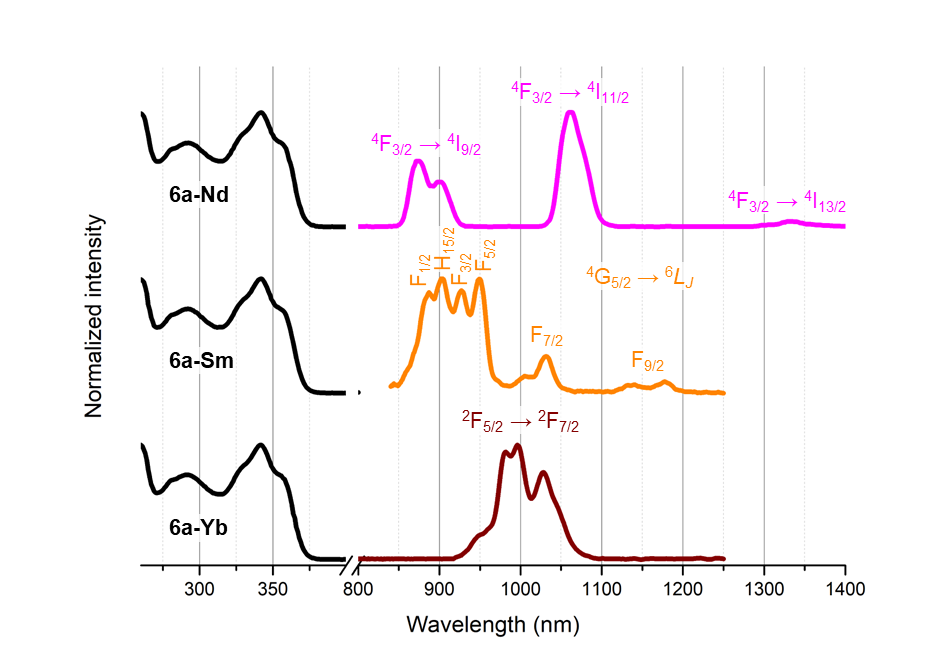


**Figure S4.** Normalized absorption spectra (in black lines) and normalized emission spectra (in coloured lines) in the NIR region of **6a-Nd**, **6a-Sm**, and **6a-Yb** (*λ*_ex_ = 342 nm).


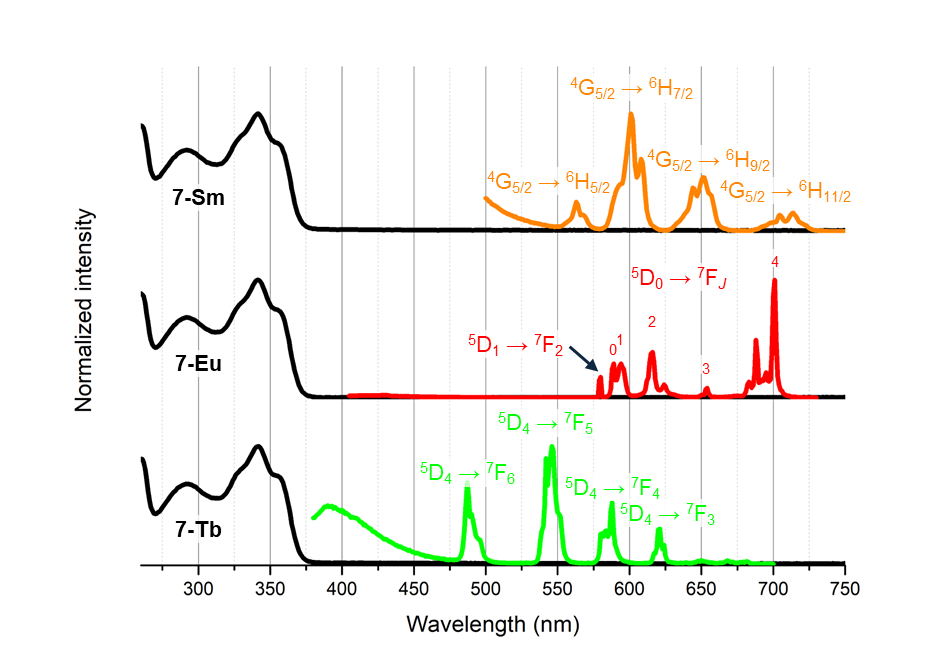


**Figure S5.** Normalized absorption spectra (in black lines) and normalized emission spectra (in coloured lines) in the visible region of **7-Sm**, **7-Eu**, and **7-Tb** (*λ*_ex_ = 342 nm).


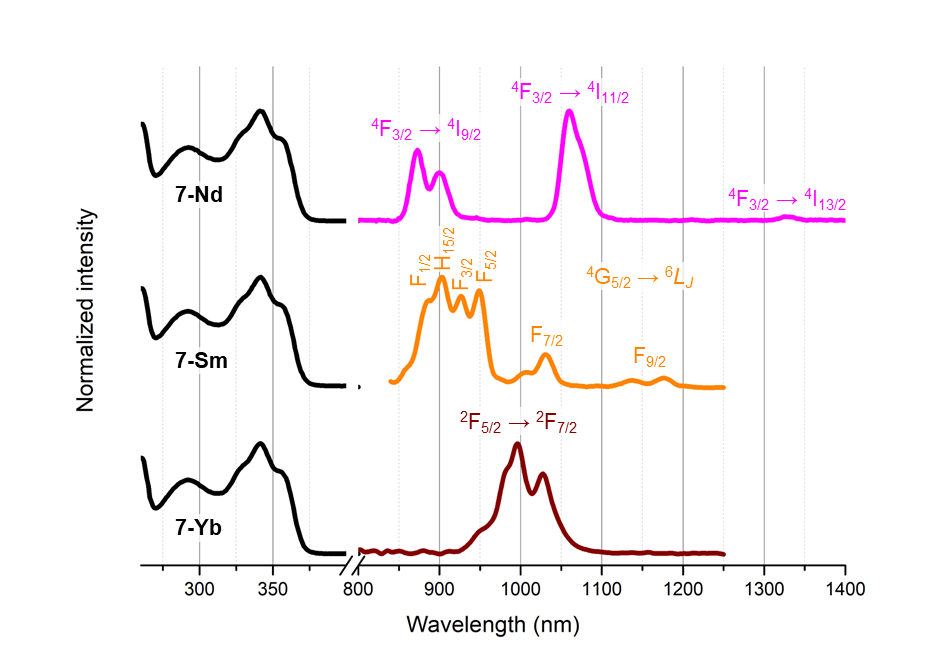


**Figure S6.** Normalized absorption spectra (in black lines) and normalized emission spectra (in coloured lines) in the NIR region of **7-Nd**, **7-Sm**, and **7-Yb** (*λ*_ex_ = 342 nm).

# Biological experiments

HeLa (cervical adenocarcinoma cell line), MDA-MB-231 (breast adenocarcinoma cell line), C17 (nasopharyngeal carcinoma cell line) and MRC-5 (lung fibroblast cell line) were used in the cellular imaging of the synthesized probes in this study. HeLa and MDA-MB-231 were cultured in Dulbecco's Modified Eagle Medium (DMEM; Gibco), C17 was cultured in Roswell Park Memorial Institute 1640 medium (RPMI 1640; Gibco). and MRC5 was cultured in Minimum Essential Medium (MEM; Gibco). DMEM and MEM were supplemented with 10% v/v fetal bovine serum (FBS; Gibco) and 1% v/v 10,000 U/mL Penicillin-Streptomycin (PS; Gibco), while RPMI 1640 was supplemented with 10% v/v FBS, 1% v/v PS, and 4 μM Y-27632.  All cell lines were purchased from American Type Culture Collection (ATCC) and maintained at 37°C with 5% CO_2_. Cells were incubated with luminescent lanthanide probes **9**–**12** for 4 hours. For subcellular localization studies, HeLa cells were treated separately with probes **9** and **10**. For target specificity, MRC5, MDA-231, and C17 cells were incubated with probes **11** and **12**, both individually and in combination. After incubation, cells were washed with PBS to remove excess probes and imaged using Nikon AXR confocal microscope to visualize probe localization upon 375 nm excitation.


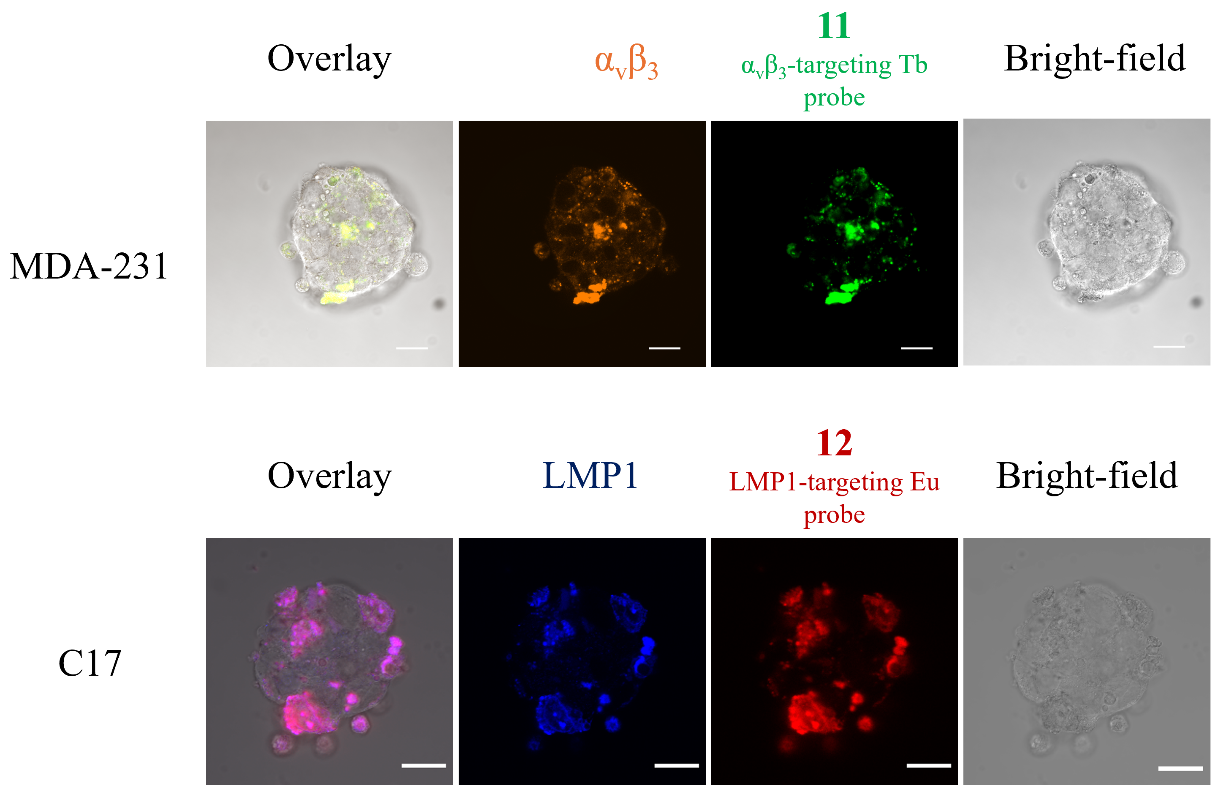


**Figure S7.** The cellular imaging of synthesized probes **11** and **12** with the corresponding antibodies of protein of interest. (scale bar: 20 µm, concentration of probe: 10 µM)

# Reference

1. Wan, F., Liu, M., Zhang, J., Li, Y. & Jiang, L. Synthesis and characterization of DOTA-mono-adamantan-1-ylamide. *Res. Chem. Intermed.* **41**, 5109–5119 (2015).

2. Kathuria, A. *et al.* Substrate specificity of acetoxy derivatives of coumarins and quinolones towards Calreticulin mediated transacetylation: Investigations on antiplatelet function. *Bioorganic Med. Chem.* **20**, 1624–1638 (2012).

3. Kovacs, D. *et al.* Photophysics of Coumarin and Carbostyril-Sensitized Luminescent Lanthanide Complexes: Implications for Complex Design in Multiplex Detection. *J. Am. Chem. Soc.* **139**, 5756–5767 (2017).

# HPLC chromatograms and HRMS spectra of products

Analytical HPLC chromatogram of Boc-Ser(*^t^*Bu)-CS124^CF3^ **S1**

_
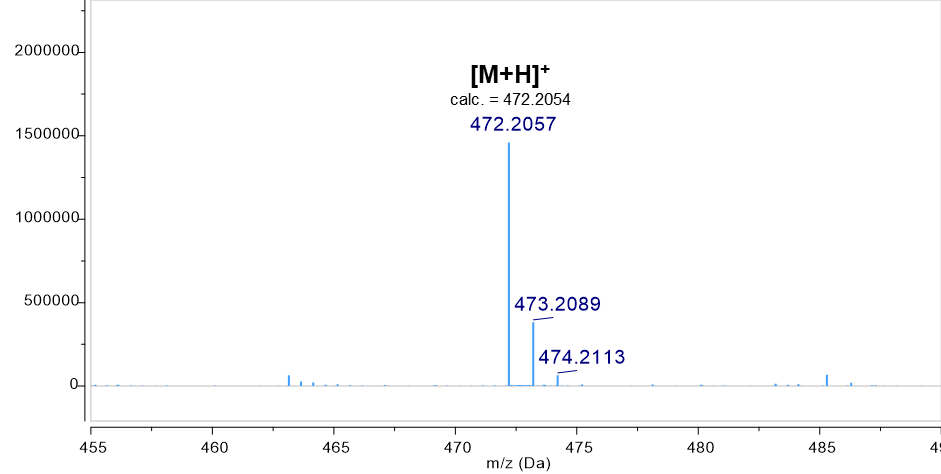
_

HRMS (ESI-QTOF) spectrum of Boc-Ser(*^t^*Bu)-CS124^CF3^ **S1**

Analytical HPLC chromatogram of H-Ser-CS124^CF3^ **S2**


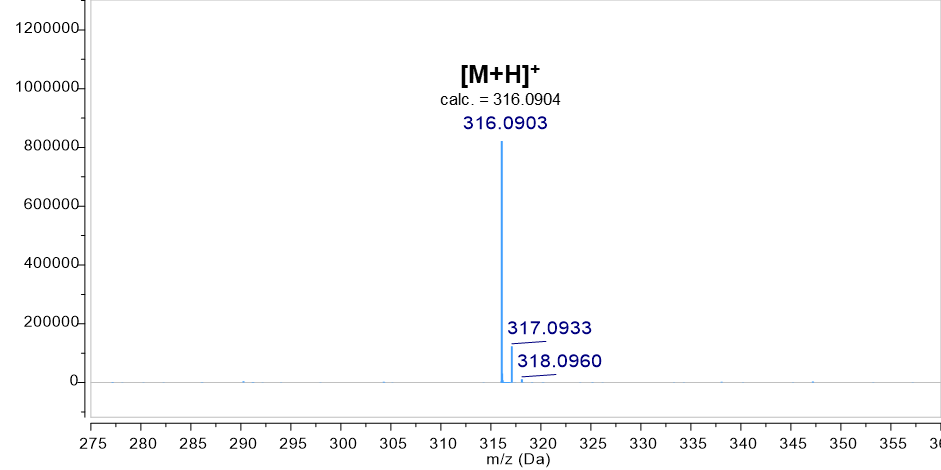


HRMS (ESI-QTOF) spectrum of H-Ser-CS124^CF3^ **S2**

Analytical HPLC chromatogram of Oxo-Ac-CS124^CF3^ **2b**


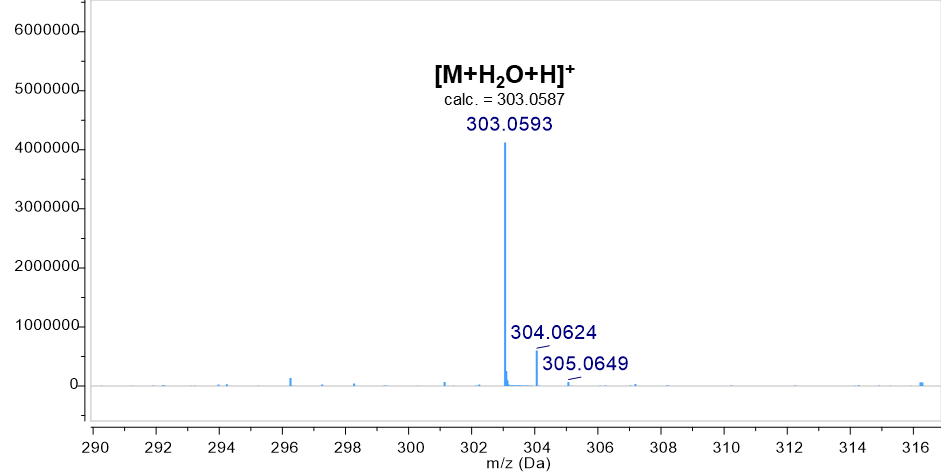


HRMS (ESI-QTOF) spectrum of Oxo-Ac-CS124^CF3^ **2b**

Analytical HPLC chromatogram of *t*-butyl ester **S3**


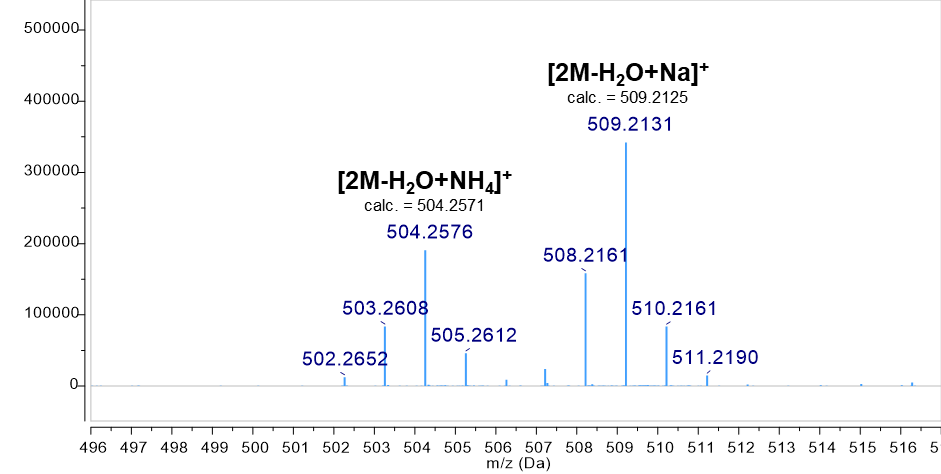


HRMS (ESI-QTOF) spectrum of *t*-butyl ester **S3**

Analytical HPLC chromatogram of acetic acid **S4**


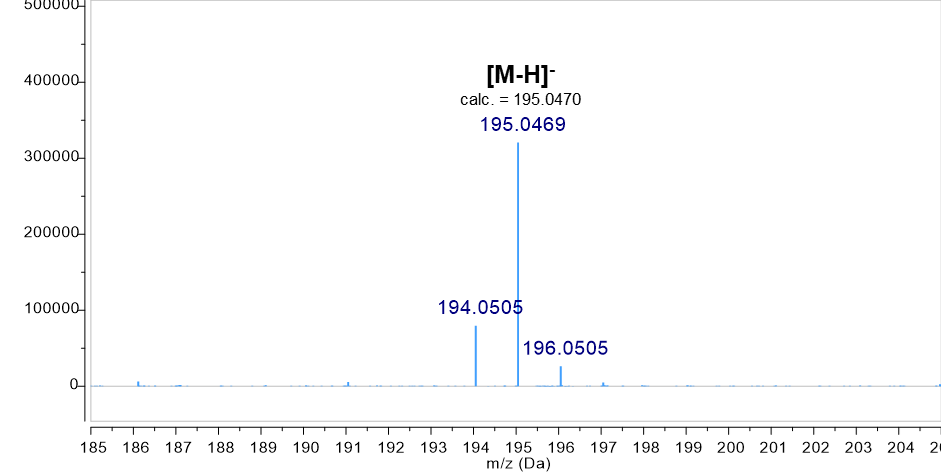


HRMS (ESI-QTOF) spectrum of acetic acid **S4**

Analytical HPLC chromatogram of methyl hexanoate **S5**


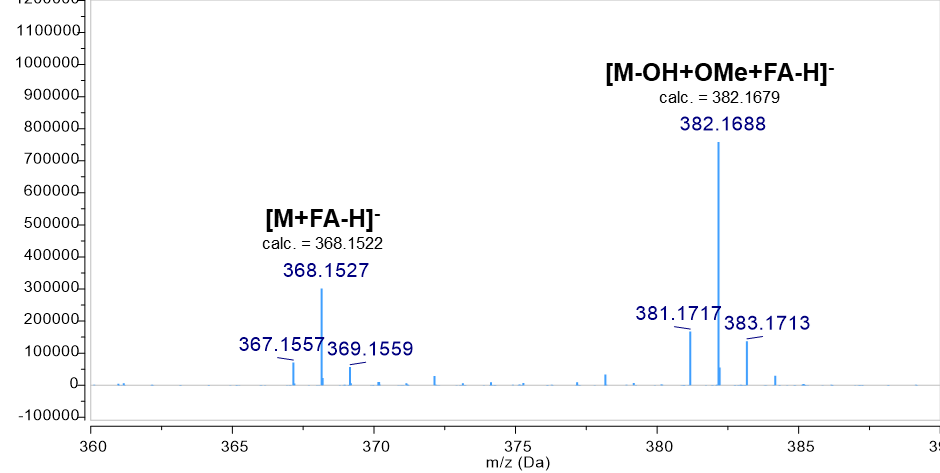


HRMS (ESI-QTOF) spectrum of methyl hexanoate **S5**

Analytical HPLC chromatogram of hexanoic acid **S6**


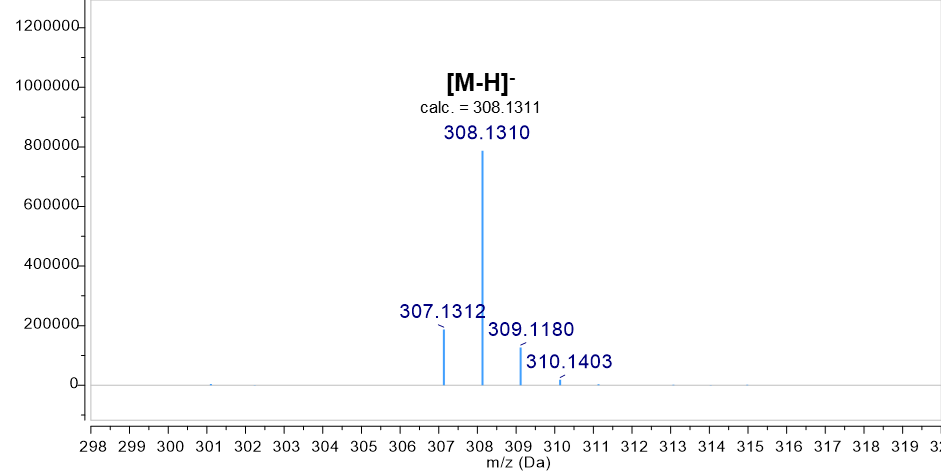


HRMS (ESI-QTOF) spectrum of hexanoic acid **S6**

Analytical HPLC chromatogram of alkyne-bearing phenylboronic acid **3c**


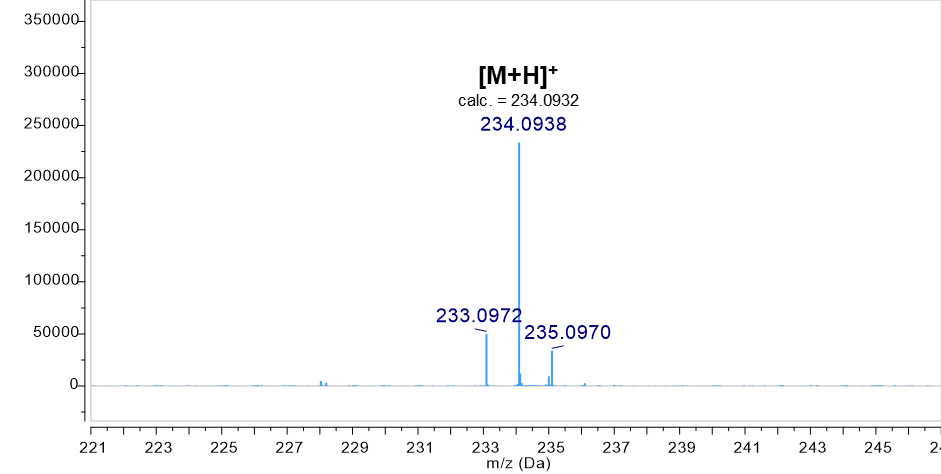


HRMS (ESI-QTOF) spectrum of alkyne-bearing phenylboronic acid **3c**

Analytical HPLC chromatogram of tetrazine-bearing phenylboronic acid **3d**


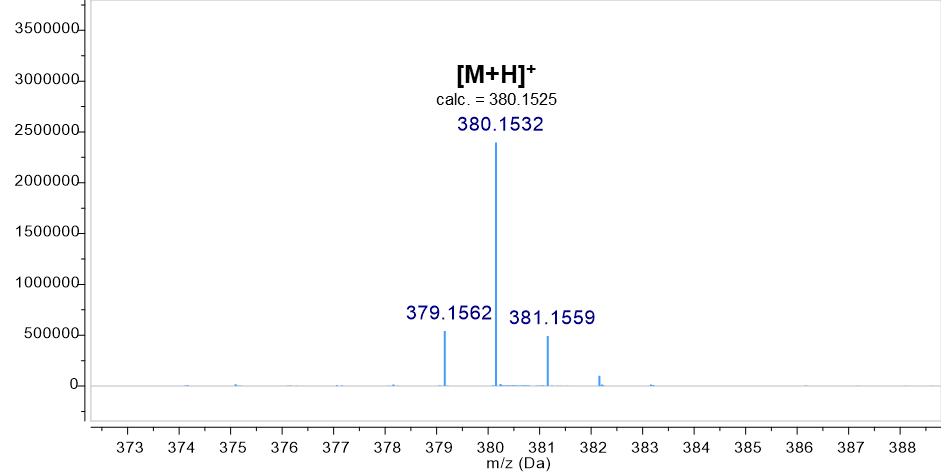


HRMS (ESI-QTOF) spectrum of tetrazine-bearing phenylboronic acid **3d**

Analytical HPLC chromatogram of DBCO-bearing phenylboronic acid **3e**


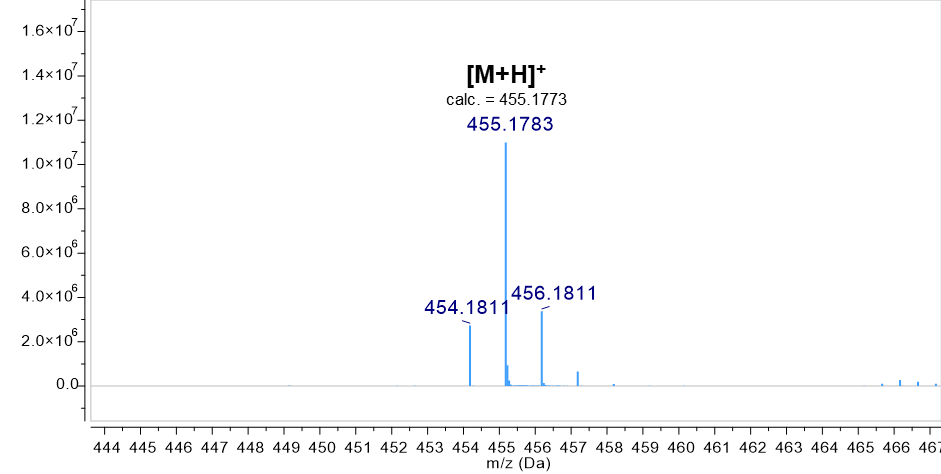


HRMS (ESI-QTOF) spectrum of DBCO-bearing phenylboronic acid **3e**

Analytical HPLC chromatogram of maleimide-bearing phenylboronic acid **3f**


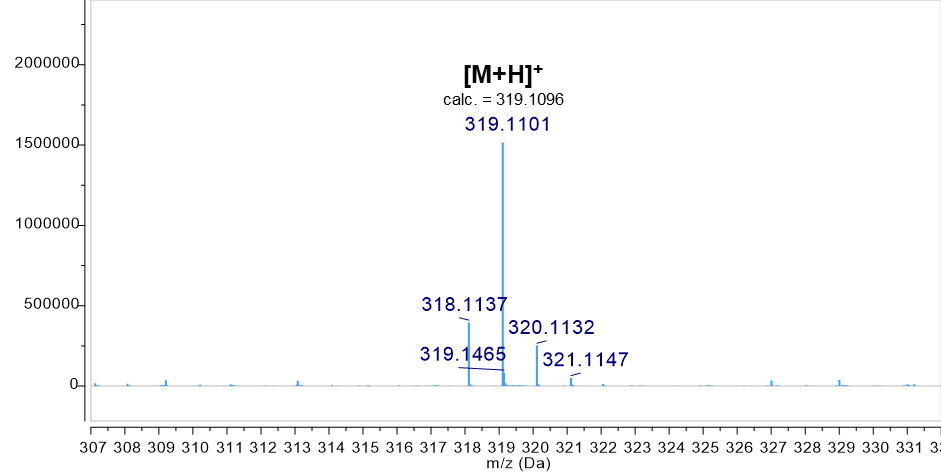


HRMS (ESI-QTOF) spectrum of maleimide-bearing phenylboronic acid **3f**

Analytical HPLC chromatogram of (*N*-Boc)Amine-bearing phenylboronic acid **3f-NHBoc**


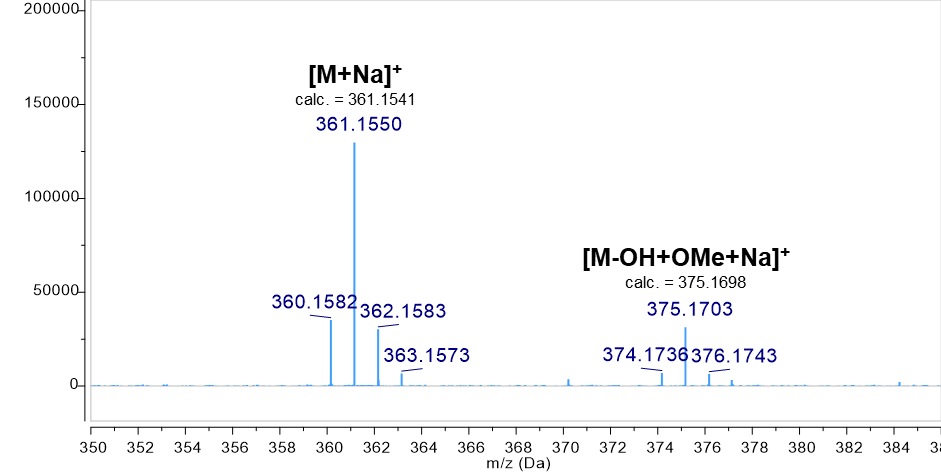


HRMS (ESI-QTOF) spectrum of (*N*-Boc)Amine-bearing phenylboronic acid **3f-NHBoc**

Analytical HPLC chromatogram of phenylboronic acid of RGD conjugate **3h**


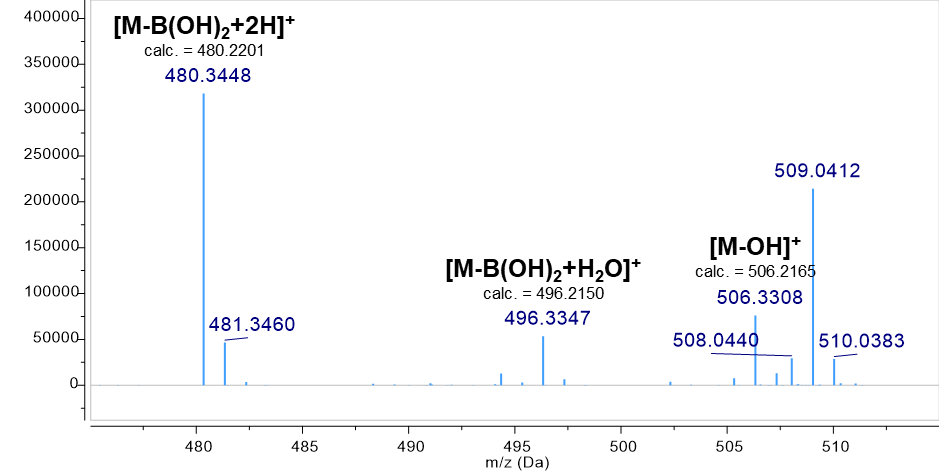


HRMS (MALDI-TOF) spectrum of phenylboronic acid of RGD conjugate **3h**

Analytical HPLC chromatogram of phenylboronic acid of *c*(RGDyK) conjugate **3k**


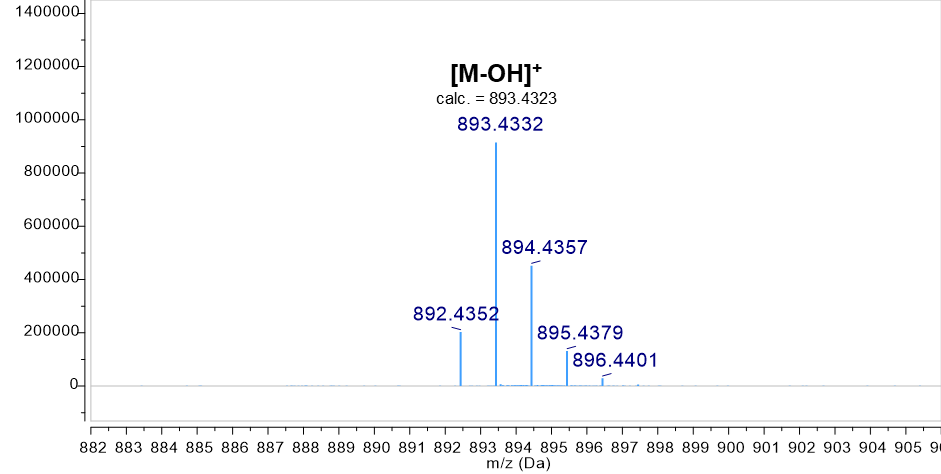


HRMS (ESI-QTOF) spectrum of phenylboronic acid of *c*(RGDyK) conjugate **3k**

Analytical HPLC chromatogram of α-aryl-substituted DOTA **4a**


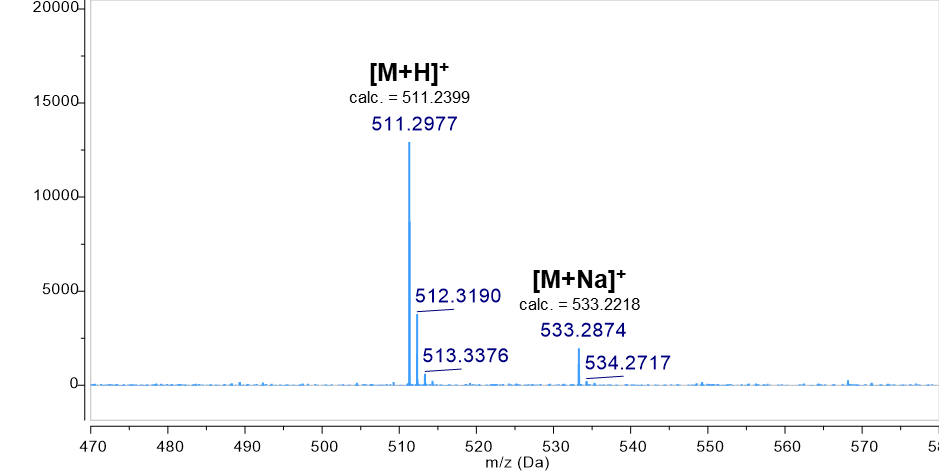


HRMS (MALDI-TOF) spectrum of α-aryl-substituted DOTA **4a**

Analytical HPLC chromatogram of α-aryl-substituted DOTA-azide **4b**


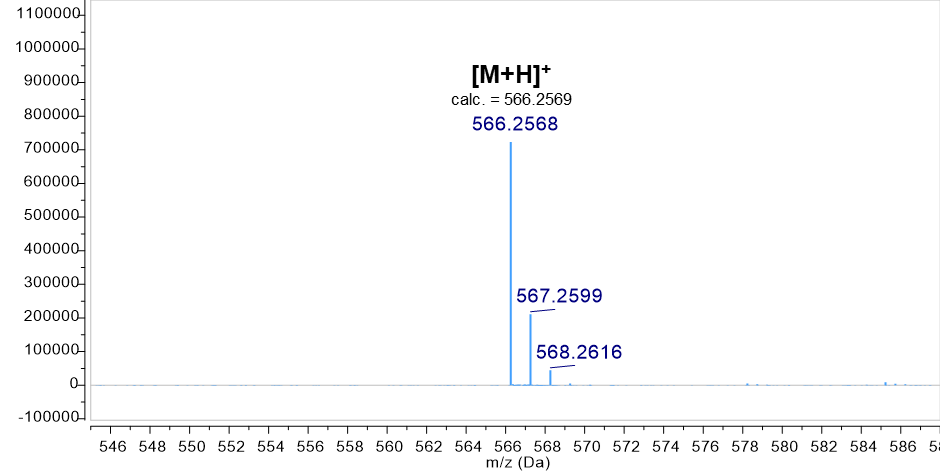


HRMS (ESI-QTOF) spectrum of α-aryl-substituted DOTA-azide **4b**

Analytical HPLC chromatogram of α-aryl-substituted DOTA-alkyne **4c**


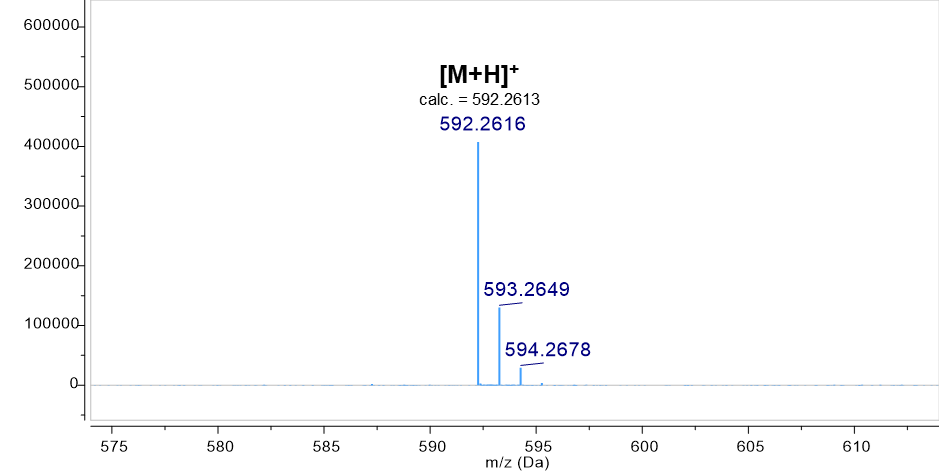


HRMS (ESI-QTOF) spectrum of α-aryl-substituted DOTA-alkyne **4c**

Analytical HPLC chromatogram of α-aryl-substituted DOTA-tetrazine **4d**


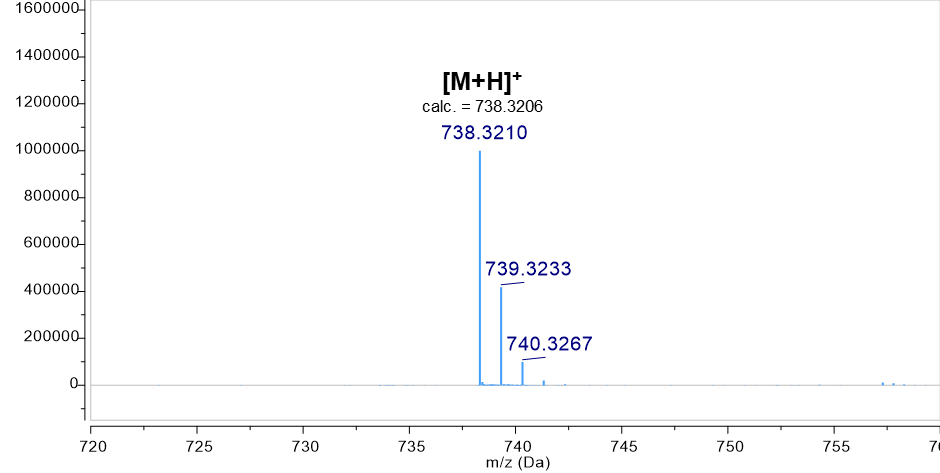


HRMS (ESI-QTOF) spectrum of α-aryl-substituted DOTA-tetrazine **4d**

Analytical HPLC chromatogram of α-aryl-substituted DOTA-DBCO **4e**


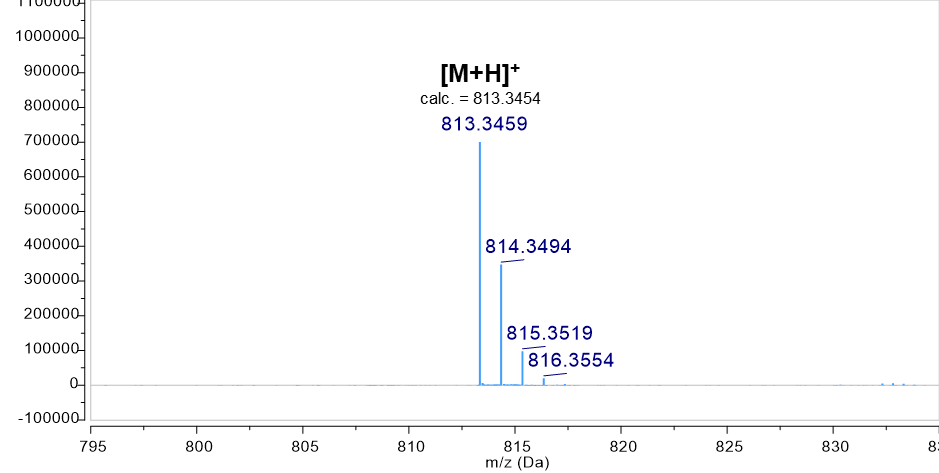


HRMS (ESI-QTOF) spectrum of α-aryl-substituted DOTA-DBCO **4e**

Analytical HPLC chromatogram of α-aryl-substituted DOTA-Et-*N*(Boc) precursor **4f-NHBoc**


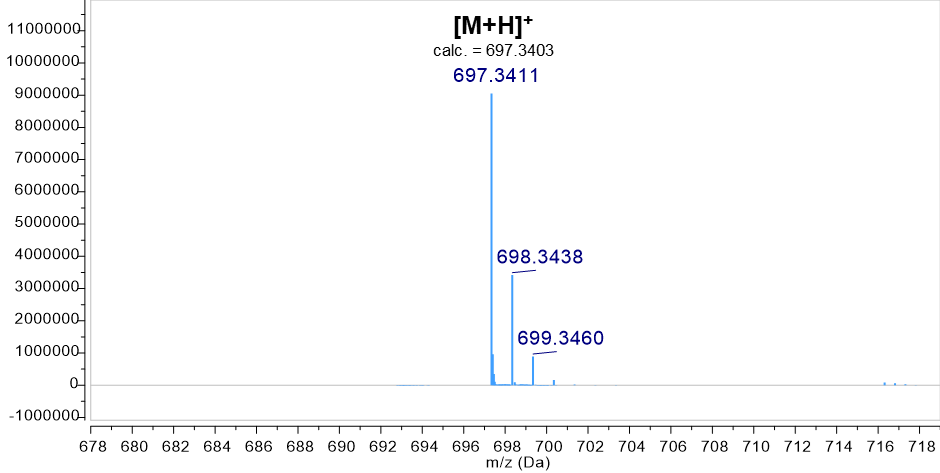


HRMS (ESI-QTOF) spectrum of α-aryl-substituted DOTA-Et-*N*(Boc) precursor **4f-NHBoc**

Analytical HPLC chromatogram of α-aryl-substituted DOTA-Et-NH_2_ precursor **4f-NH_2_**


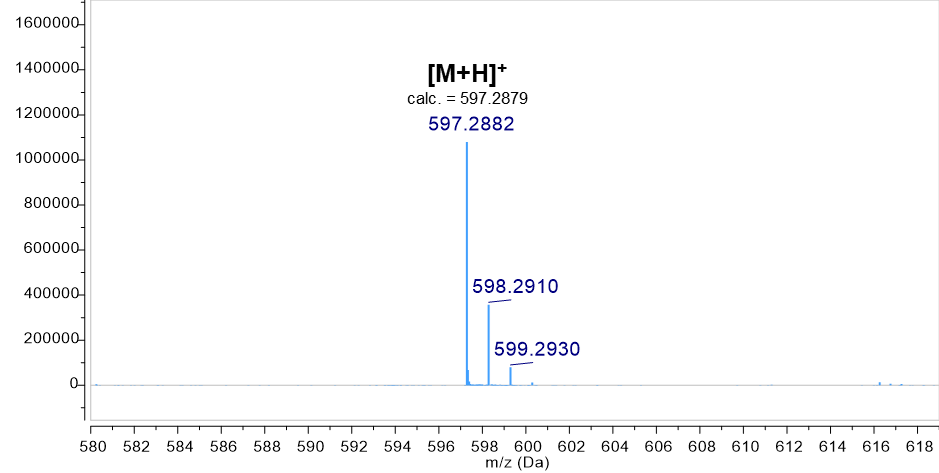


HRMS (ESI-QTOF) spectrum of α-aryl-substituted DOTA-Et-NH_2_ precursor **4f-NH_2_**

Analytical HPLC chromatogram of α-aryl-substituted DOTA-maleimide **4f**


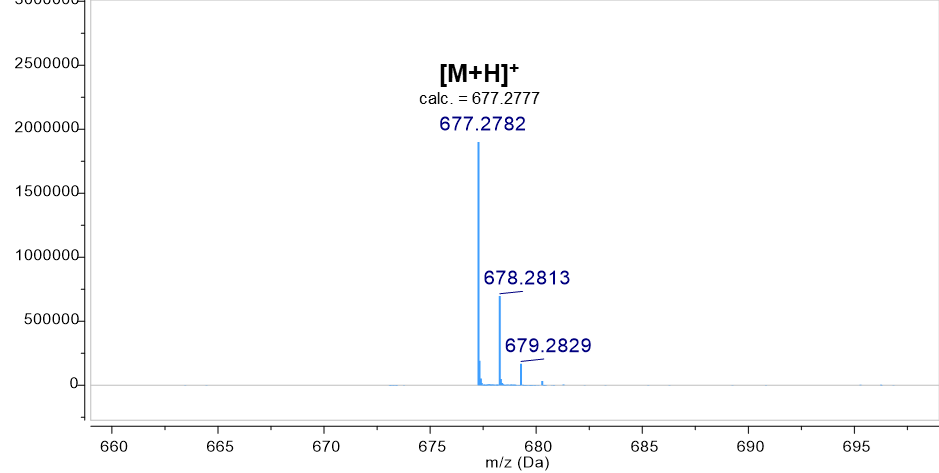


HRMS (ESI-QTOF) spectrum of α-aryl-substituted DOTA-maleimide **4f**

Analytical HPLC chromatogram of α-aryl-substituted DOTA-*N*(Boc) precursor **4g-NHBoc**


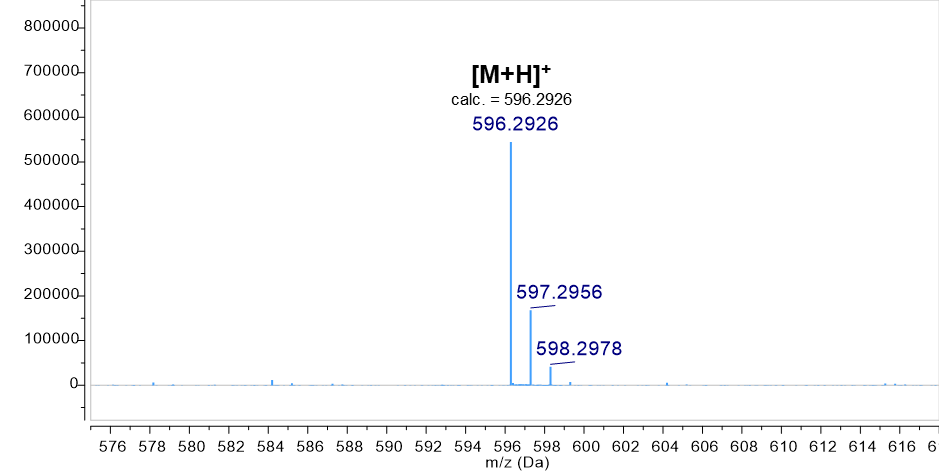


HRMS (ESI-QTOF) spectrum of α-aryl-substituted DOTA-*N*(Boc) precursor **4g-NHBoc**

Analytical HPLC chromatogram of α-aryl-substituted DOTA-isothiocyanate **4g**


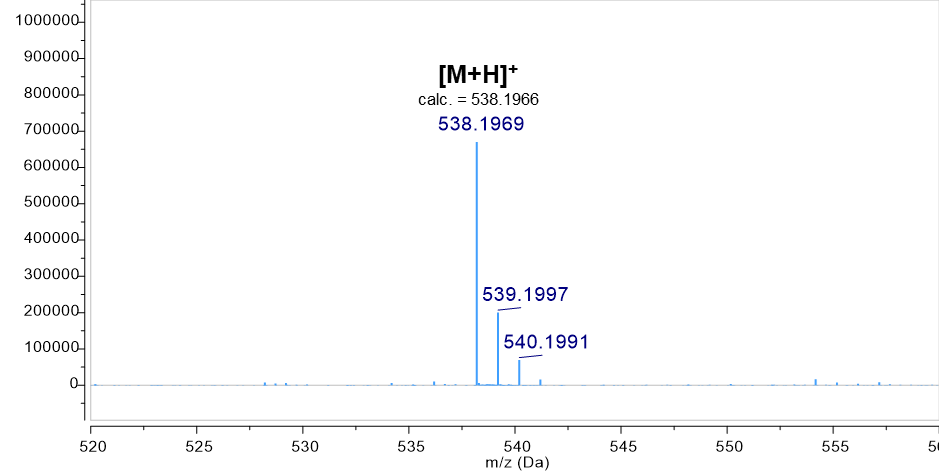


HRMS (ESI-QTOF) spectrum of α-aryl-substituted DOTA-isothiocyanate **4g**

Analytical HPLC chromatogram of α-aryl-substituted DOTA of RGD conjugate **4h**


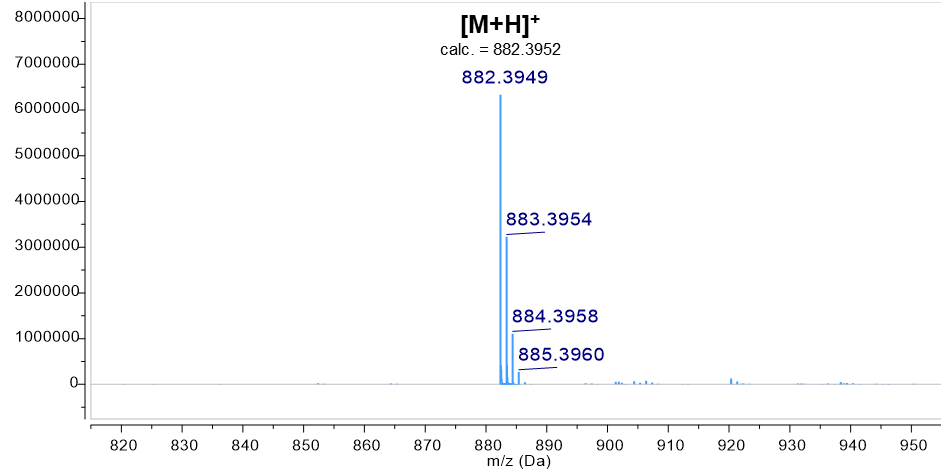


HRMS (ESI-QTOF) spectrum of α-aryl-substituted DOTA of RGD conjugate **4h**

Analytical HPLC chromatogram of α-aryl-substituted DOTA of PSMA-sequence conjugate **4i**


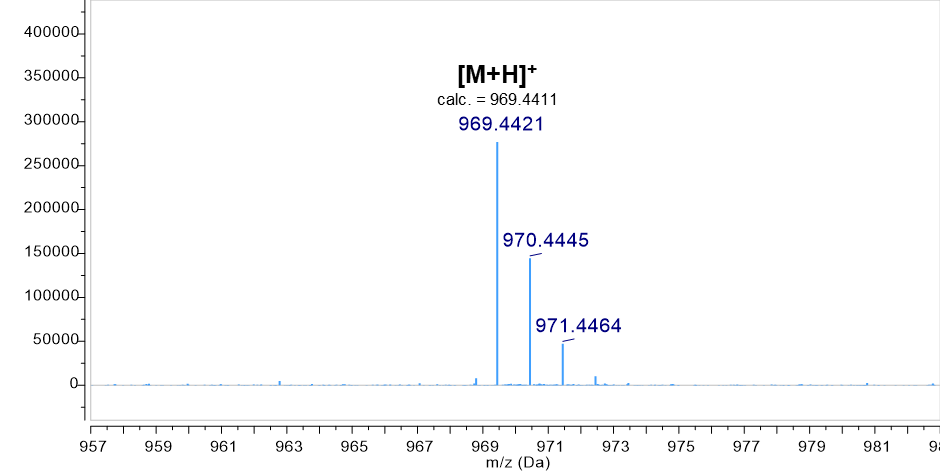


HRMS (ESI-QTOF) spectrum of α-aryl-substituted DOTA of PSMA-sequence conjugate **4i**

Analytical HPLC chromatogram of α-aryl-substituted DOTA of GE11 conjugate **4j**


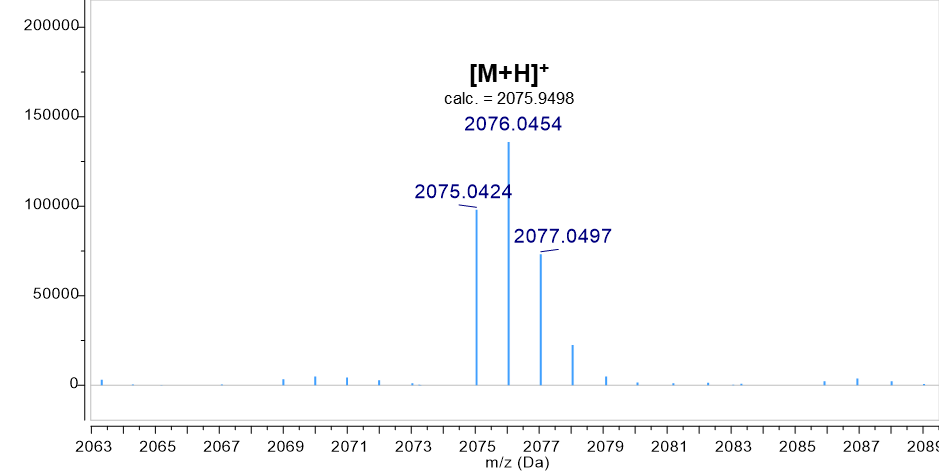


HRMS (MALDI-TOF) spectrum of α-aryl-substituted DOTA of GE11 conjugate **4j**

Analytical HPLC chromatogram of α-aryl-substituted DOTA of *c*(RGDyK) conjugate **4k**


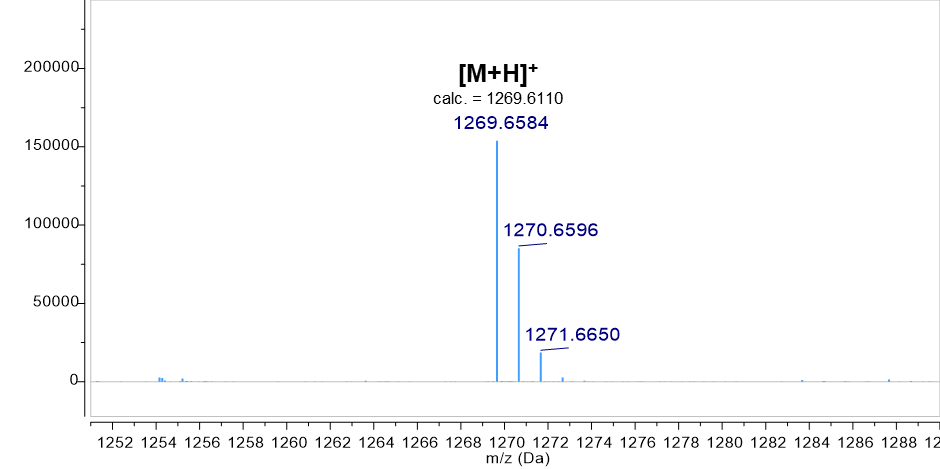


HRMS (ESI-QTOF) spectrum of α-aryl-substituted DOTA of *c*(RGDyK) conjugate **4k**

Analytical HPLC chromatogram of α-aryl-substituted Gd-DOTA **4a-Gd**


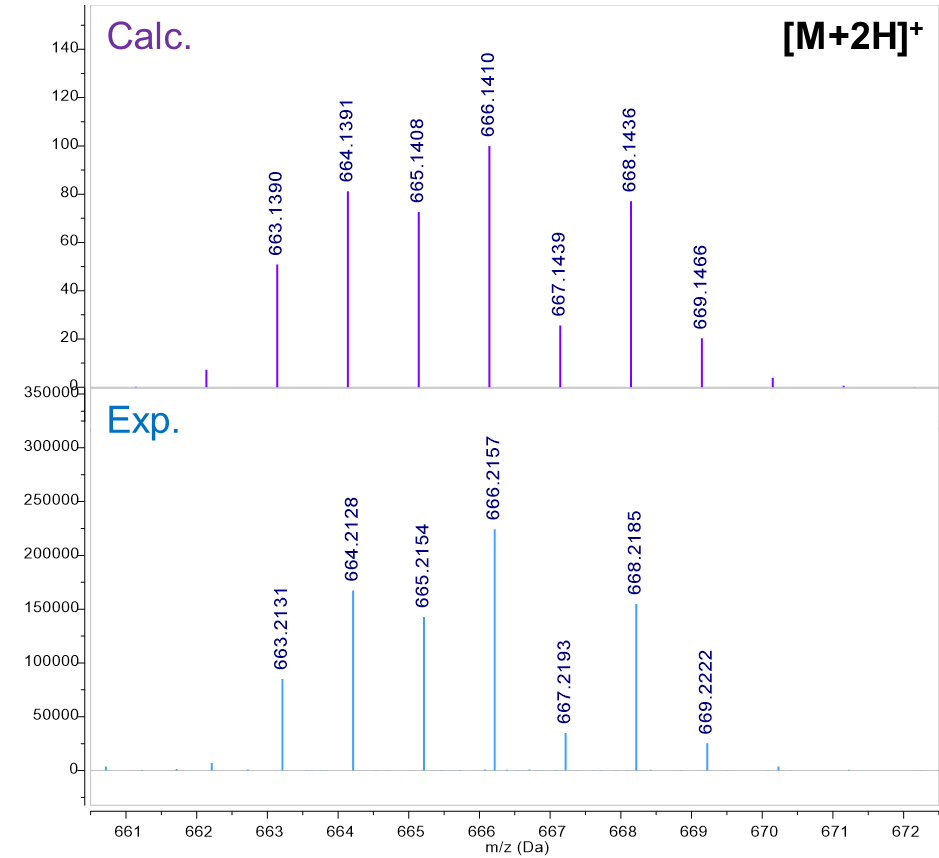


HRMS (MALDI-TOF) spectrum of α-aryl-substituted Gd-DOTA **4a-Gd**

Analytical HPLC chromatogram of α-aryl-substituted Gd-DOTA-azide **4b-Gd**


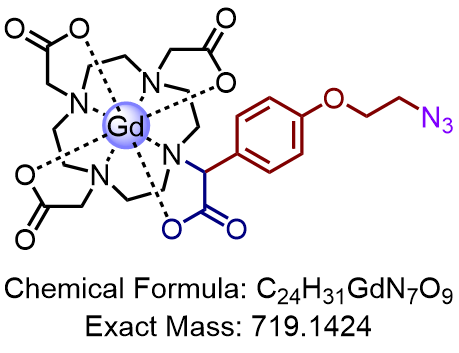


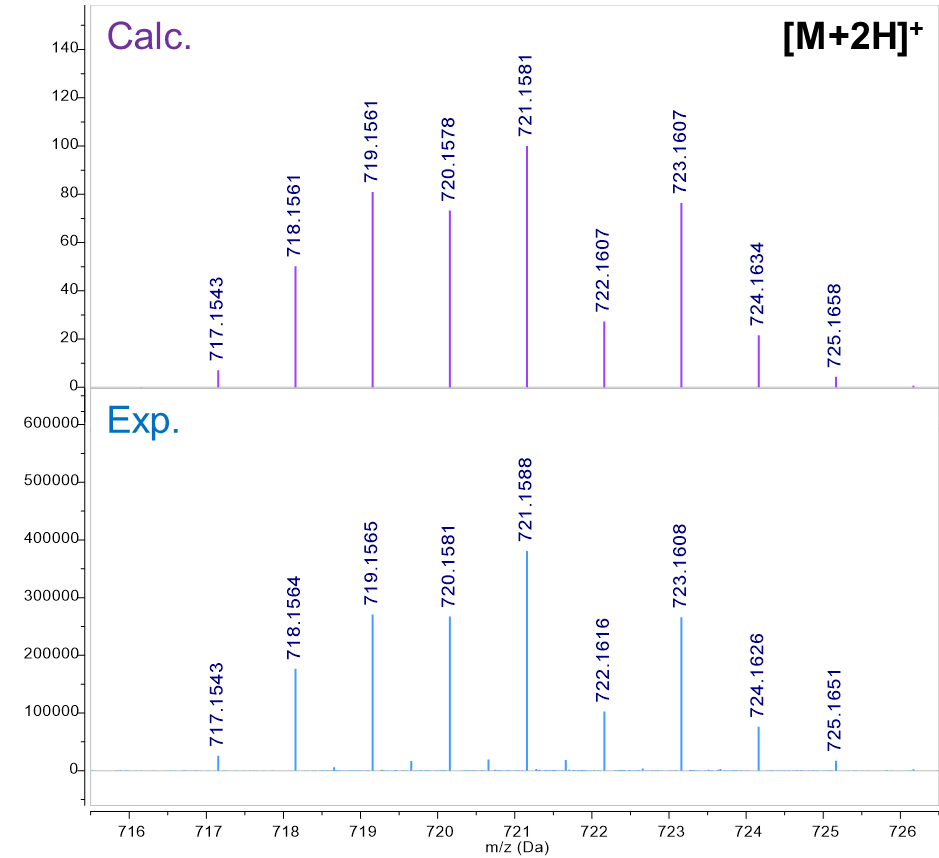


HRMS (ESI-QTOF) spectrum of α-aryl-substituted Gd-DOTA-azide **4b-Gd**

Analytical HPLC chromatogram of α-aryl-substituted Gd-DOTA-tetrazine **4d-Gd**


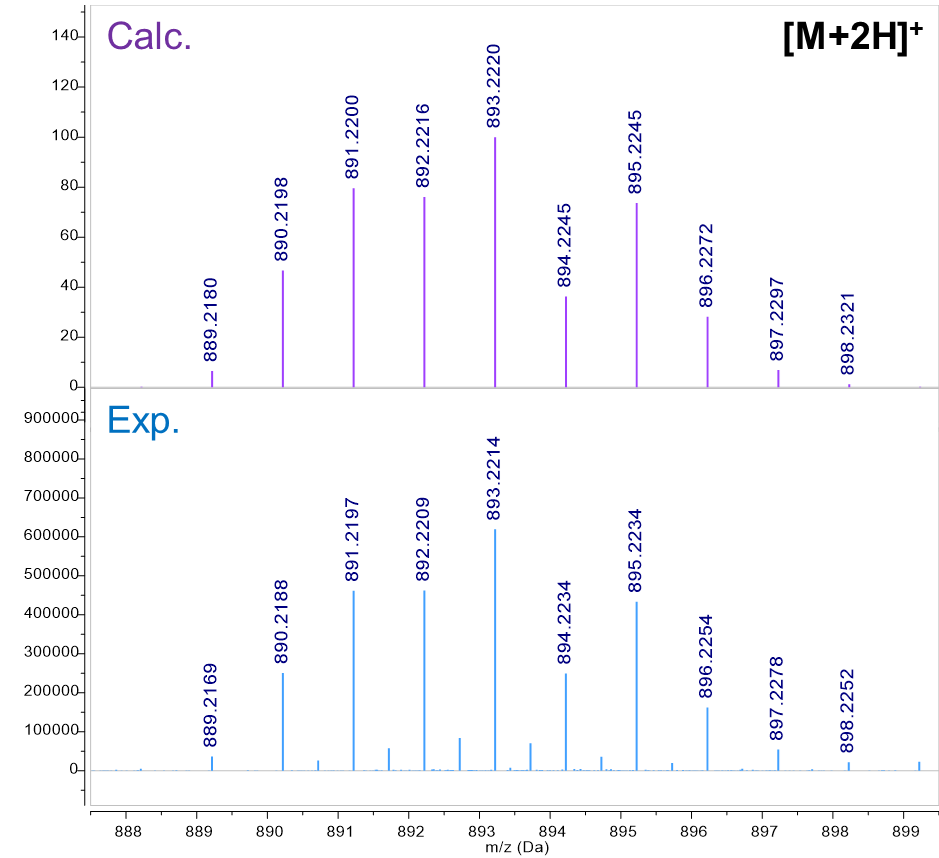


HRMS (ESI-QTOF) spectrum of α-aryl-substituted Gd-DOTA-tetrazine **4d-Gd**

Analytical HPLC chromatogram of α-aryl-substituted Gd-DOTA-maleimide **4f-Gd**


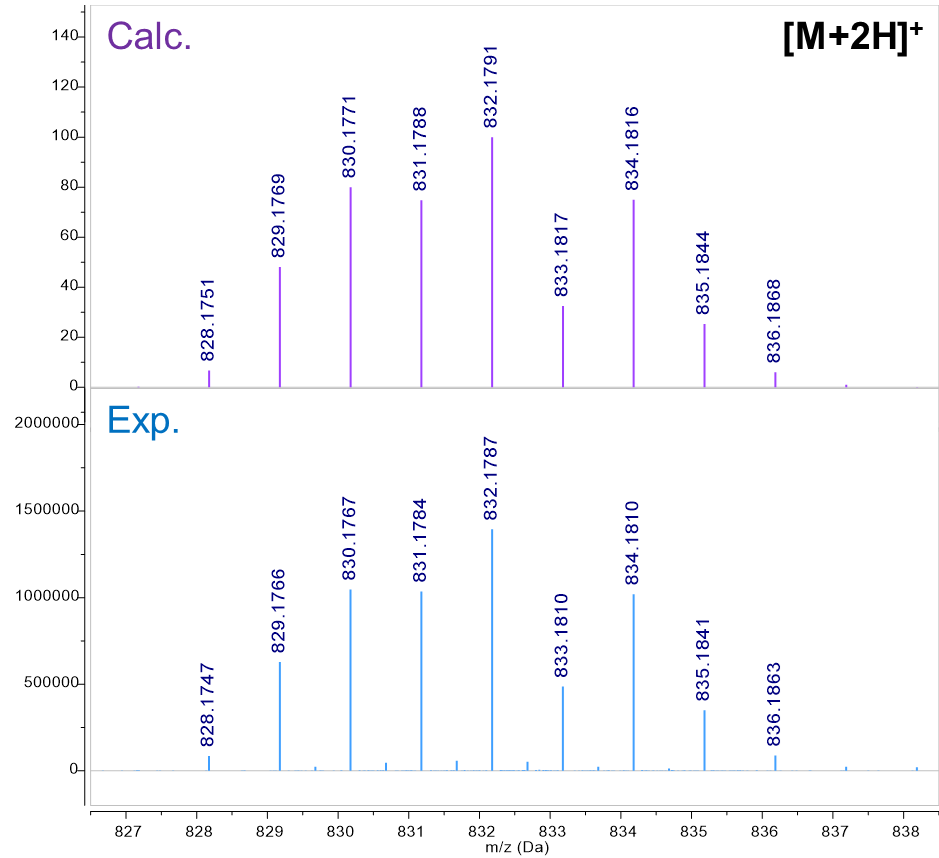


HRMS (ESI-QTOF) spectrum of α-aryl-substituted Gd-DOTA-maleimide **4f-Gd**

Analytical HPLC chromatogram of α-aryl-substituted Gd-DOTA-isothiocyanate **4g-Gd**


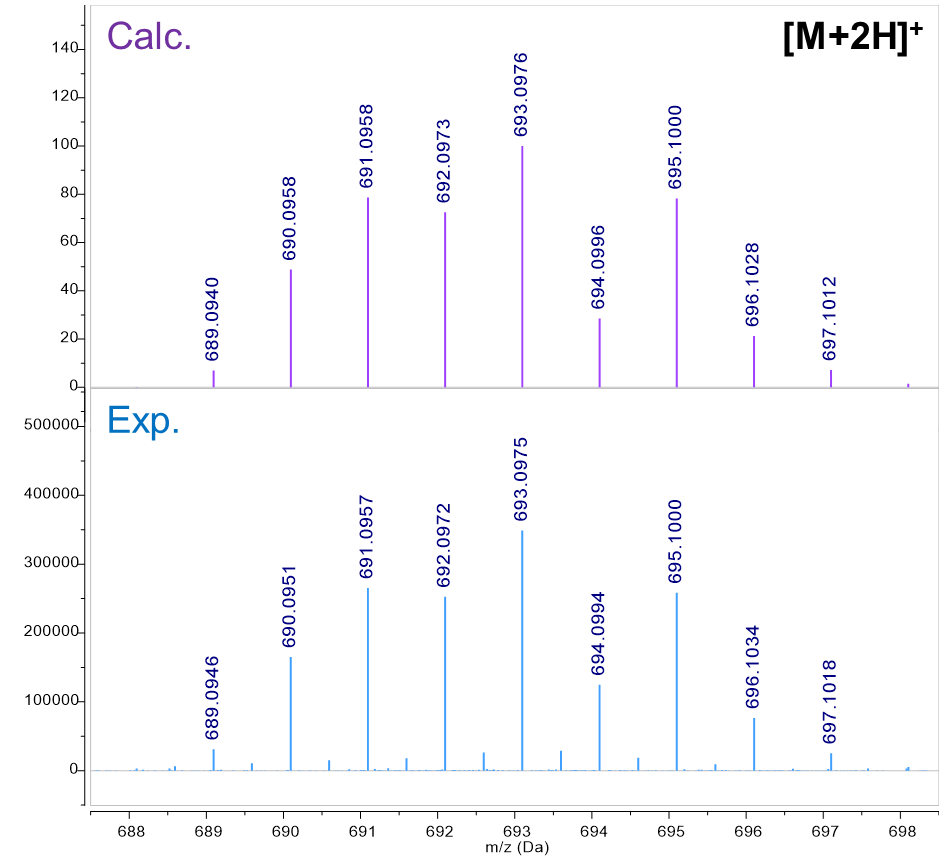


HRMS (ESI-QTOF) spectrum of α-aryl-substituted Gd-DOTA-isothiocyanate **4g-Gd**

Analytical HPLC chromatogram of α-aryl-substituted Gd-DOTA of RGD conjugate **4h-Gd**


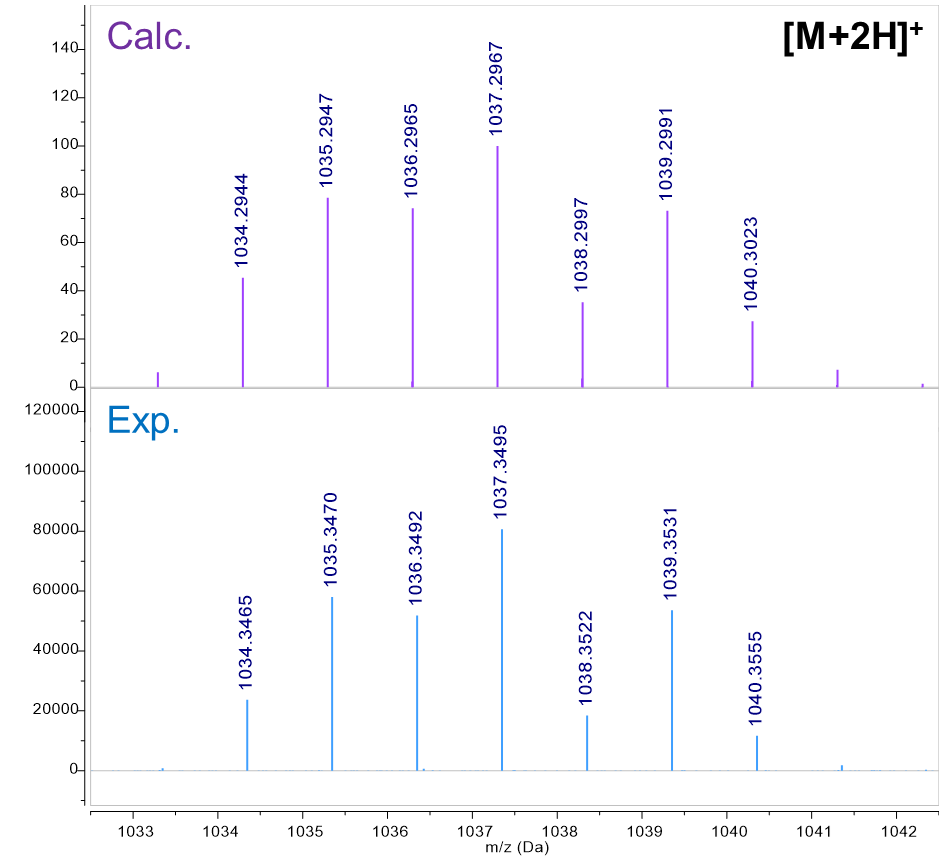


HRMS (MALDI-TOF) spectrum of α-aryl-substituted Gd-DOTA of RGD conjugate **4h-Gd**

Analytical HPLC chromatogram of α-aryl-substituted Gd-DOTA of PSMA-sequence conjugate **4i-Gd**


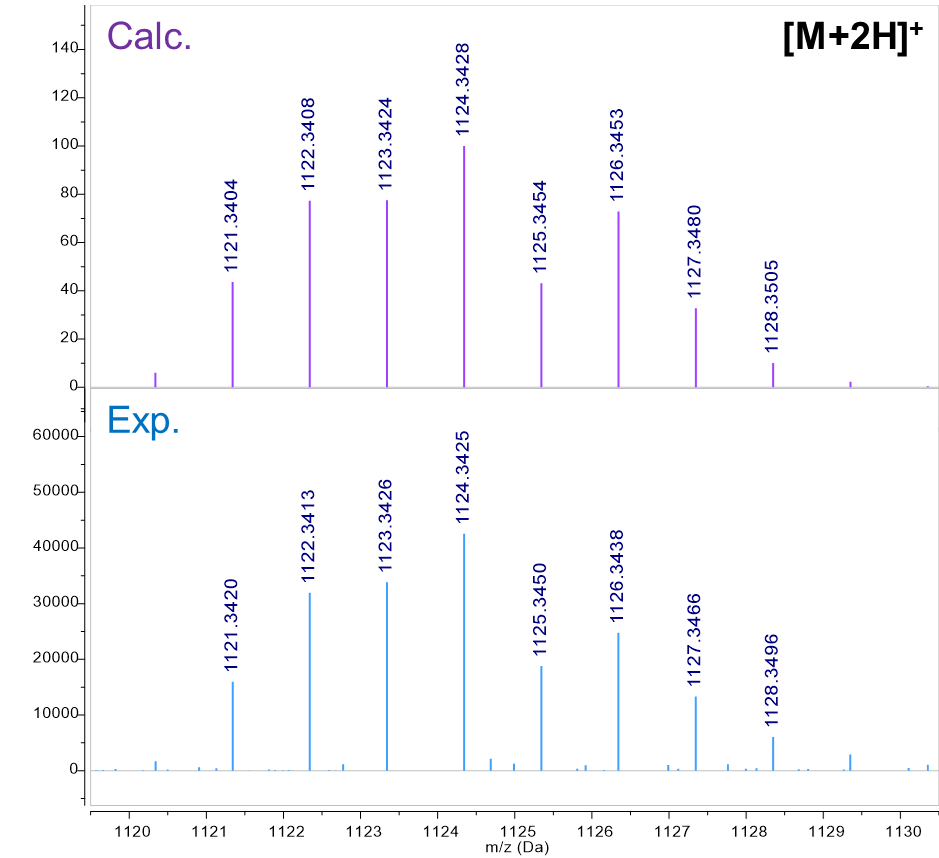


HRMS (ESI-QTOF) spectrum of α-aryl-substituted Gd-DOTA of PSMA-sequence conjugate **4i-Gd**

Analytical HPLC chromatogram of α-aryl-substituted Gd-DOTA of GE11 conjugate **4j-Gd**


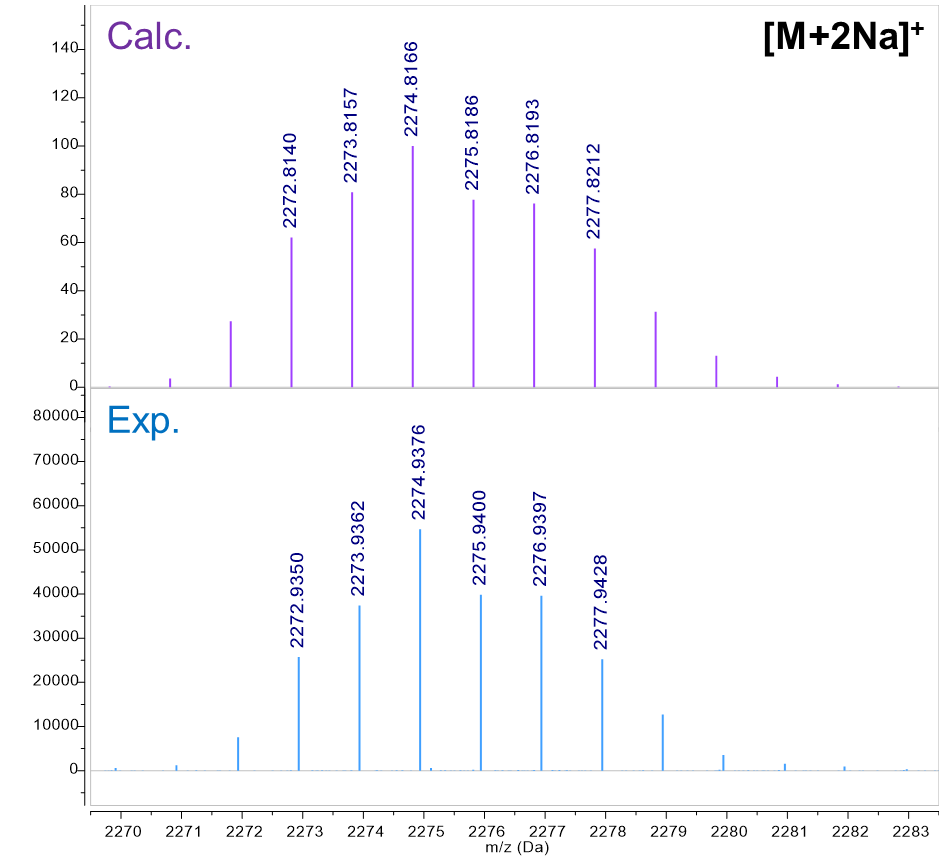


HRMS (MALDI-TOF) spectrum of α-aryl-substituted Gd-DOTA of GE11 conjugate **4j-Gd**

Analytical HPLC chromatogram of α-aryl-substituted Gd-DOTA of *c*(RGDyK) conjugate **4k-Gd**


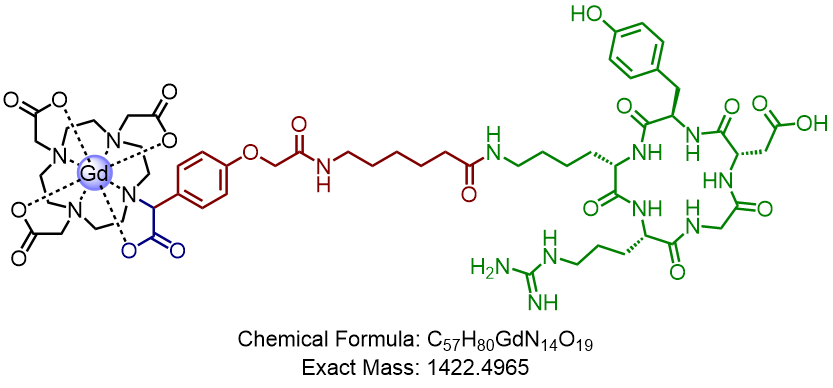


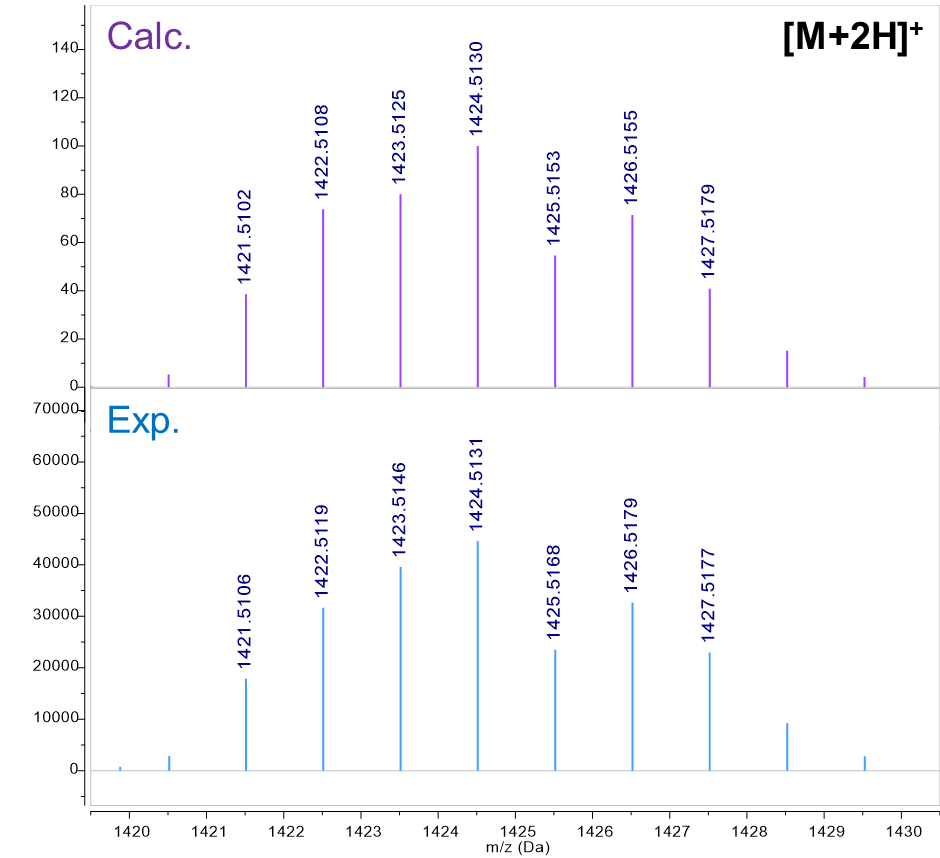


HRMS (ESI-QTOF) spectrum of α-aryl-substituted Gd-DOTA of *c*(RGDyK) conjugate **4k-Gd**

Analytical HPLC chromatogram of DO3A-Gly-Tyr(Me)-CONH_2_ **5**


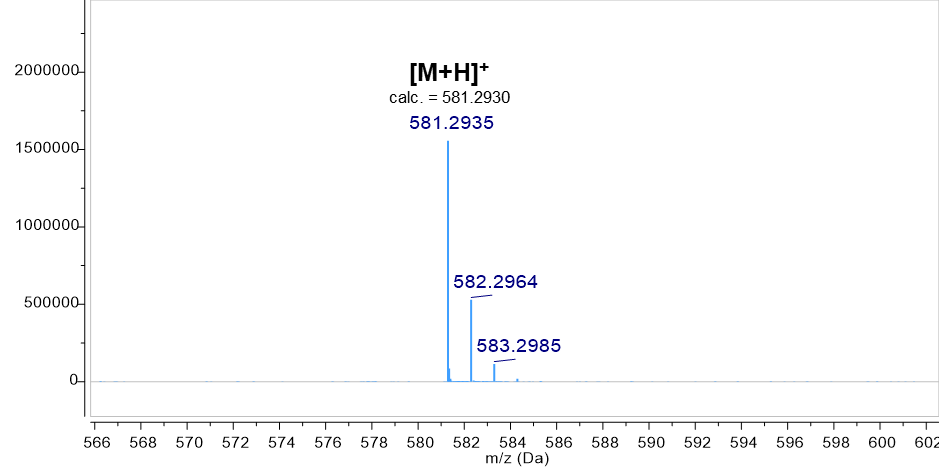


HRMS (ESI-QTOF) spectrum of DO3A-Gly-Tyr(Me)-CONH_2_ **5**

Analytical HPLC chromatogram of Gd-DO3A-Gly-Tyr(Me)-CONH_2_ **5-Gd**


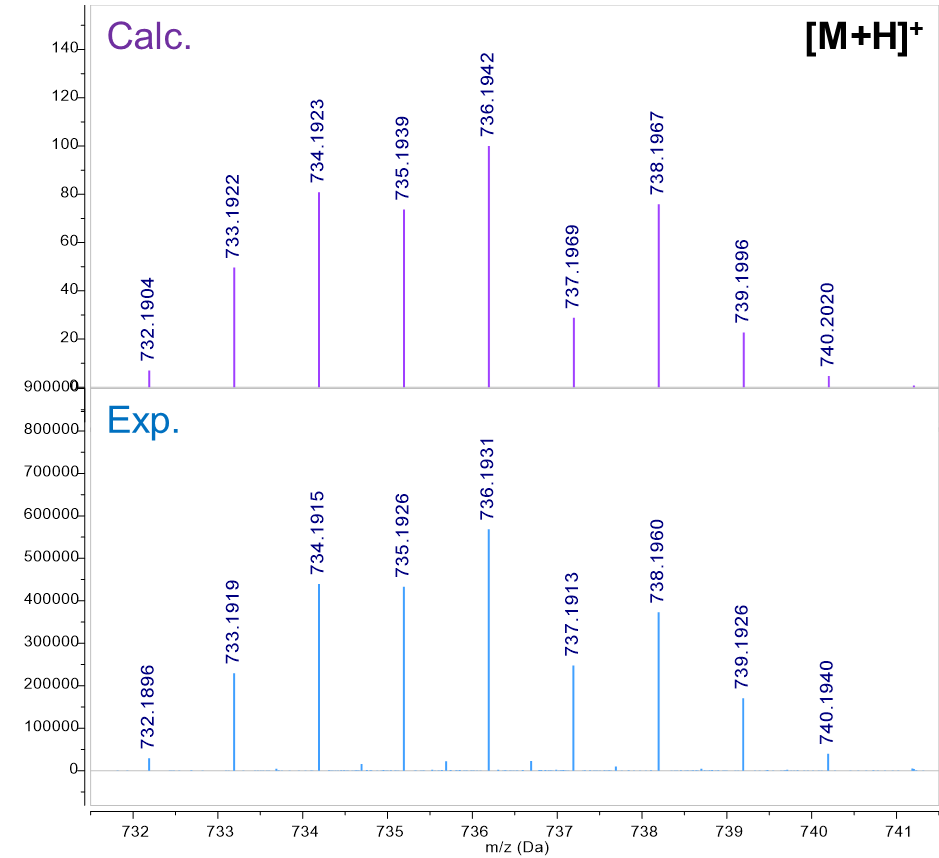


HRMS (ESI-QTOF) spectrum of Gd-DO3A-Gly-Tyr(Me)-CONH_2_ **5-Gd**

Analytical HPLC chromatogram of luminescent α-aryl-substituted DO3A **6a**


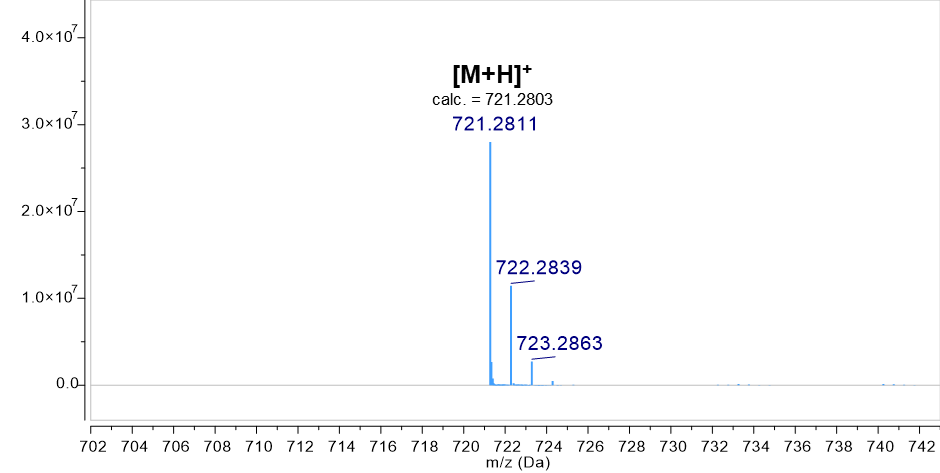


HRMS (ESI-QTOF) spectrum of luminescent α-aryl-substituted DO3A **6a**

Analytical HPLC chromatogram of **6a-Nd**


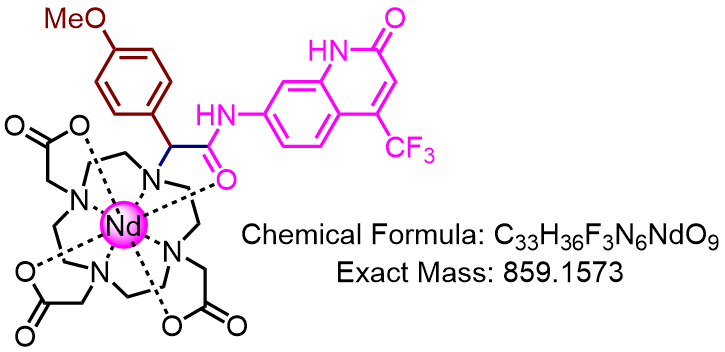


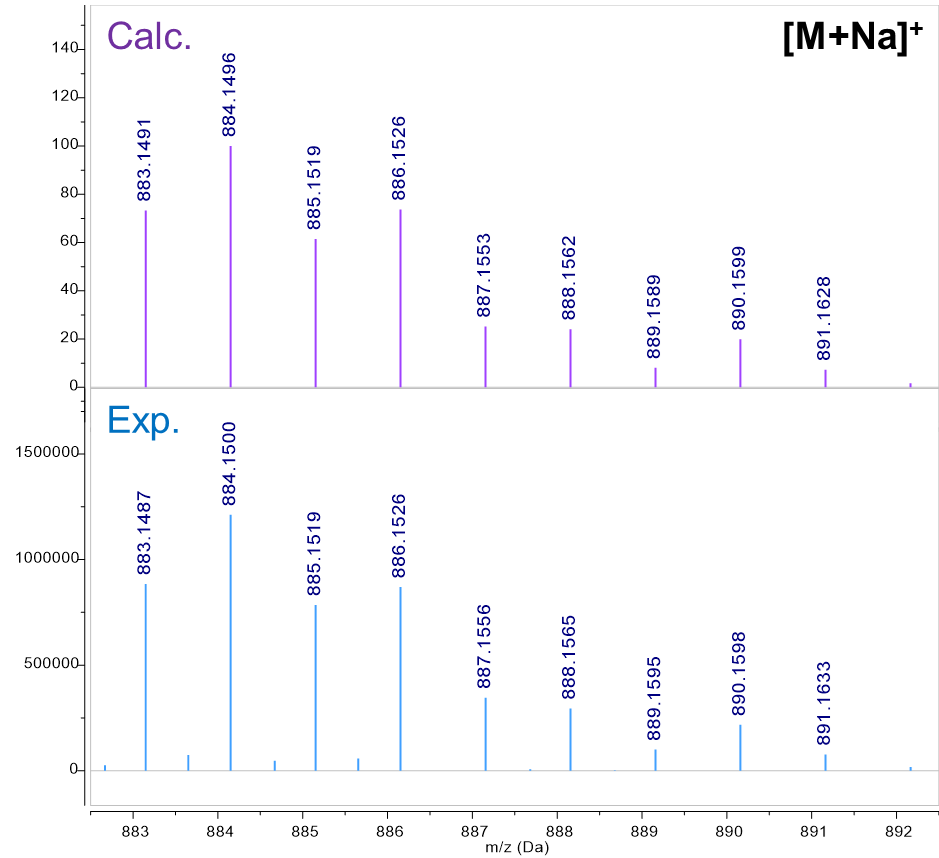


HRMS (ESI-QTOF) spectrum of **6a-Nd**

Analytical HPLC chromatogram of **6a-Sm**


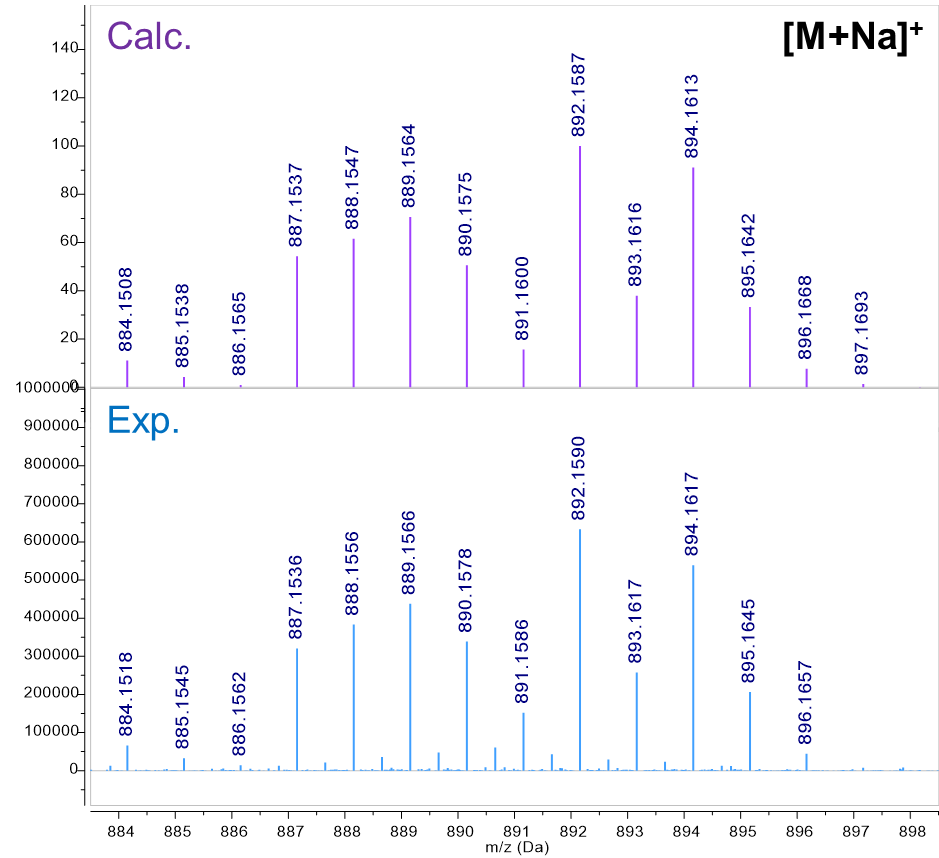


HRMS (ESI-QTOF) spectrum of **6a-Sm**

Analytical HPLC chromatogram of **6a-Eu**


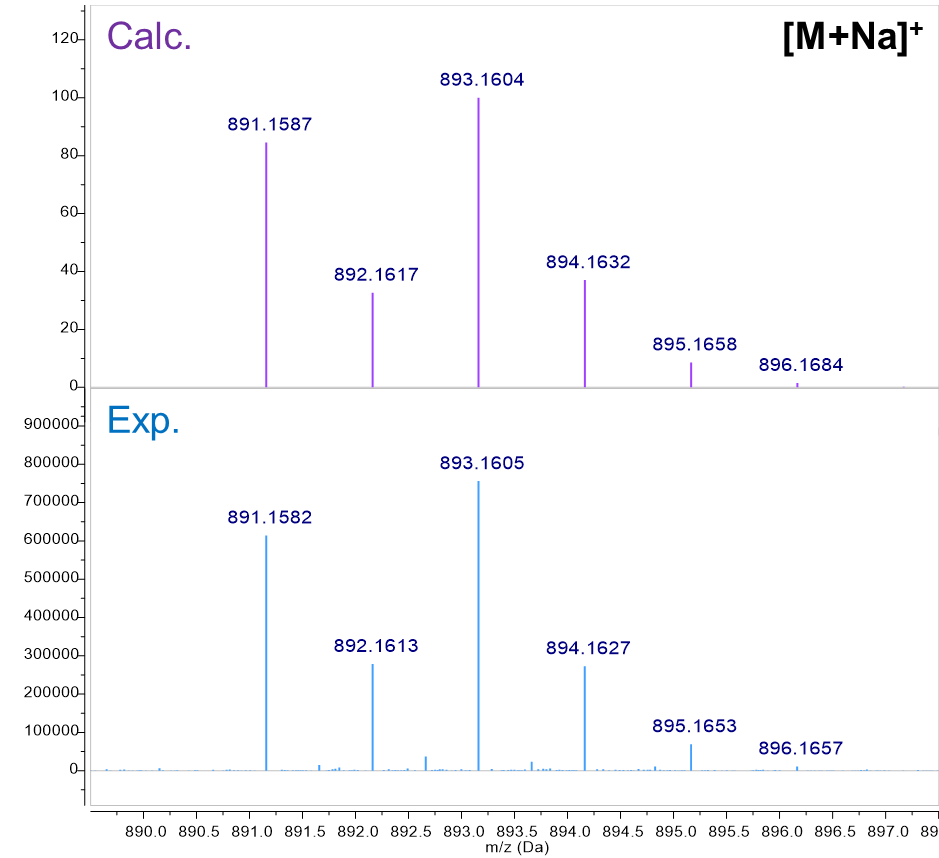


HRMS (ESI-QTOF) spectrum of **6a-Eu**

Analytical HPLC chromatogram of **6a-Tb**


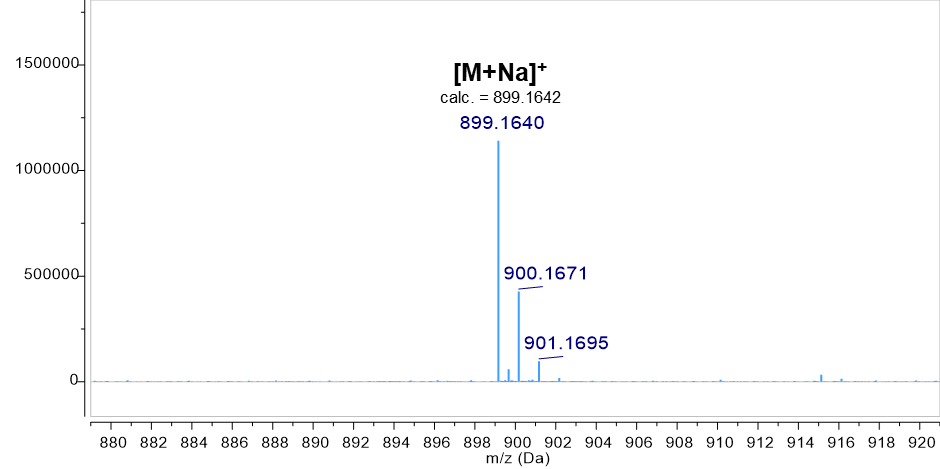


HRMS (ESI-QTOF) spectrum of **6a-Tb**

Analytical HPLC chromatogram of **6a-Yb**


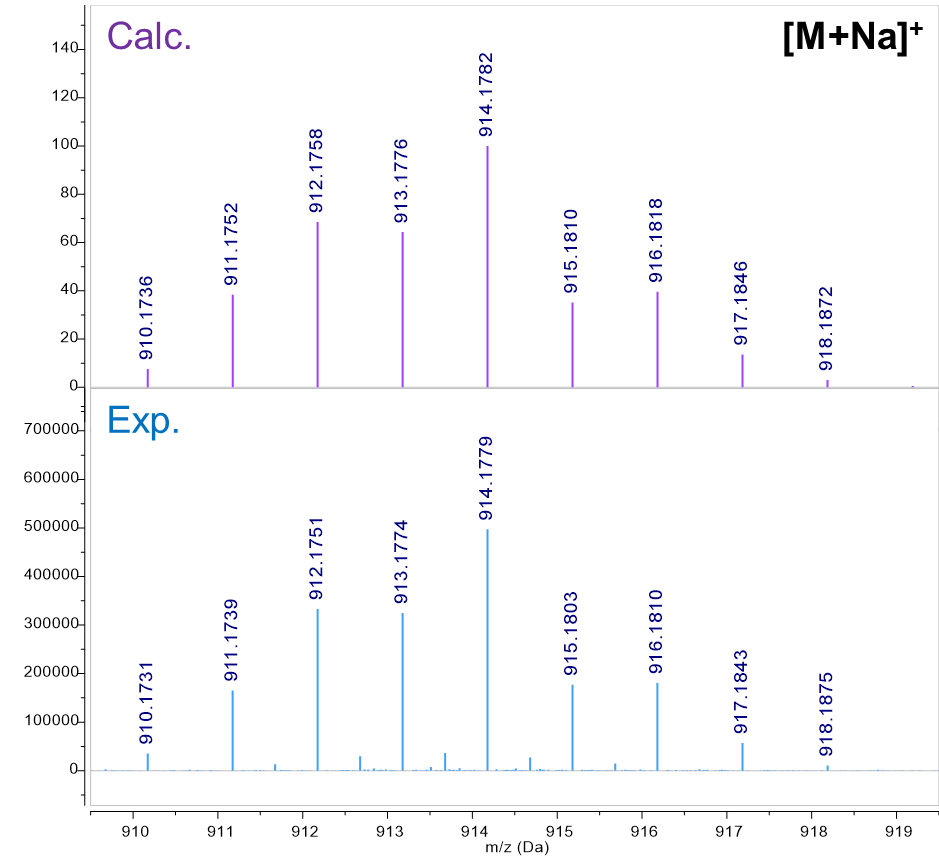


HRMS (ESI-QTOF) spectrum of **6a-Yb**

Analytical HPLC chromatogram of luminescent α-aryl-substituted DO3A-azide **6b**


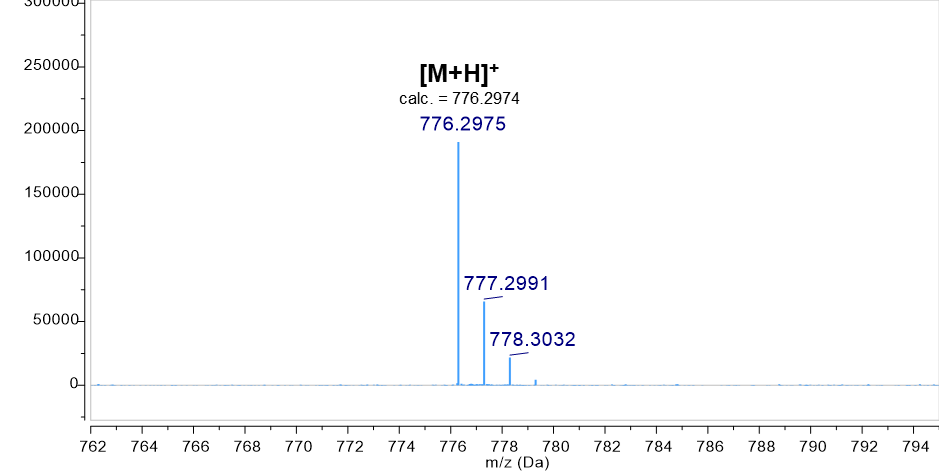


HRMS (ESI-QTOF) spectrum of luminescent α-aryl-substituted DO3A-azide **6b**

Analytical HPLC chromatogram of **6b-Eu**


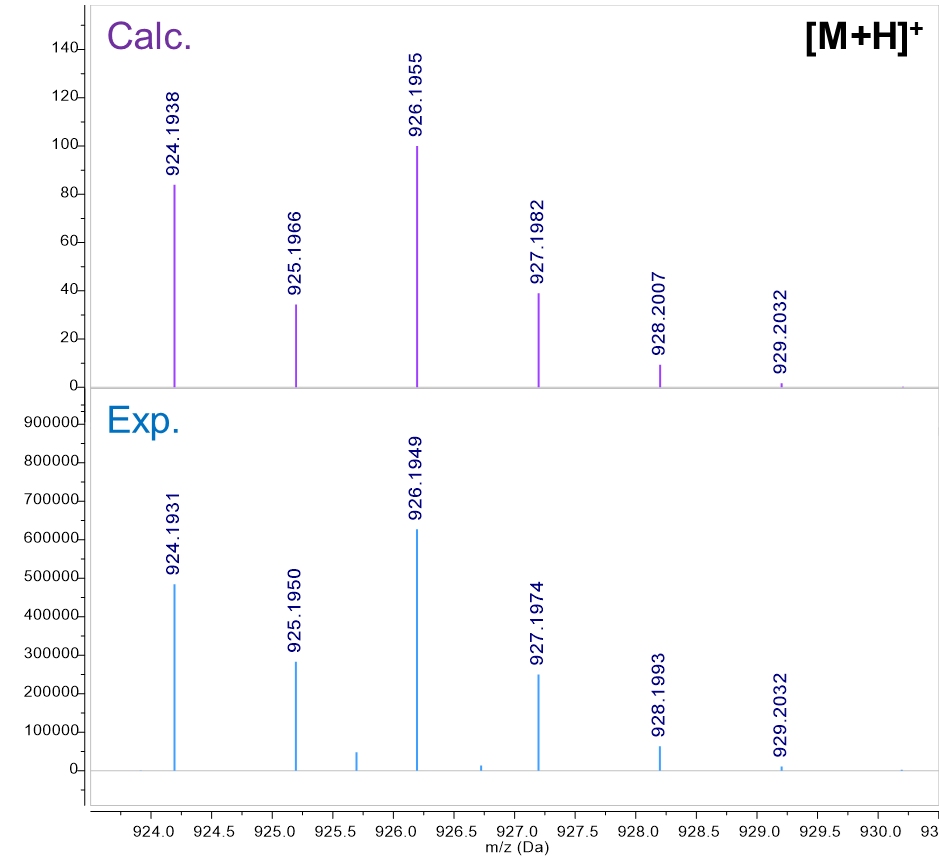


HRMS (ESI-QTOF) spectrum of **6b-Eu**

Analytical HPLC chromatogram of **6b-Tb**


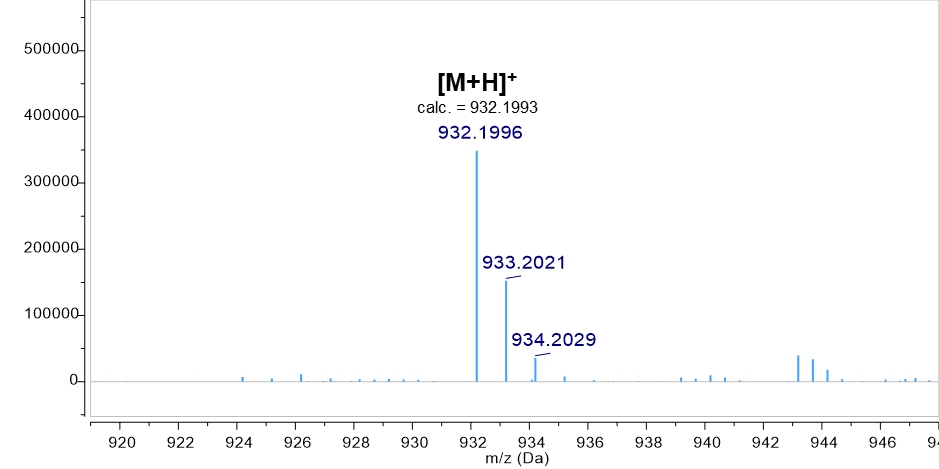


HRMS (ESI-QTOF) spectrum of **6b-Tb**

Analytical HPLC chromatogram of **7-Nd**


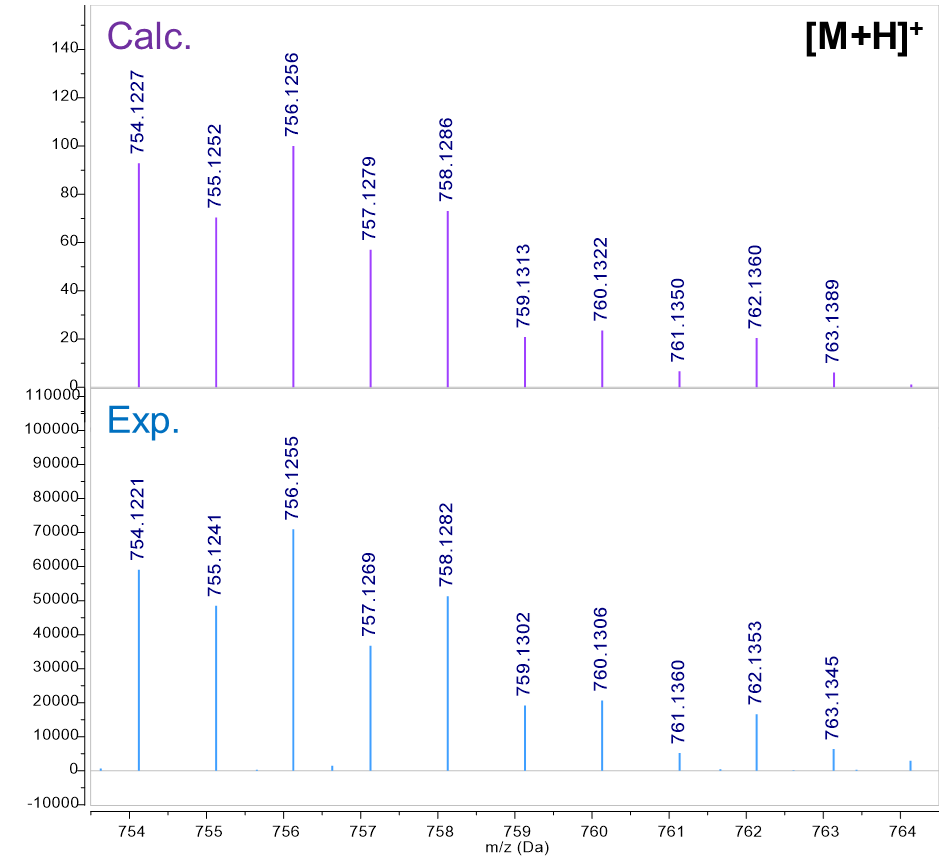


HRMS (ESI-QTOF) spectrum of **7-Nd**

Analytical HPLC chromatogram of **7-Sm**


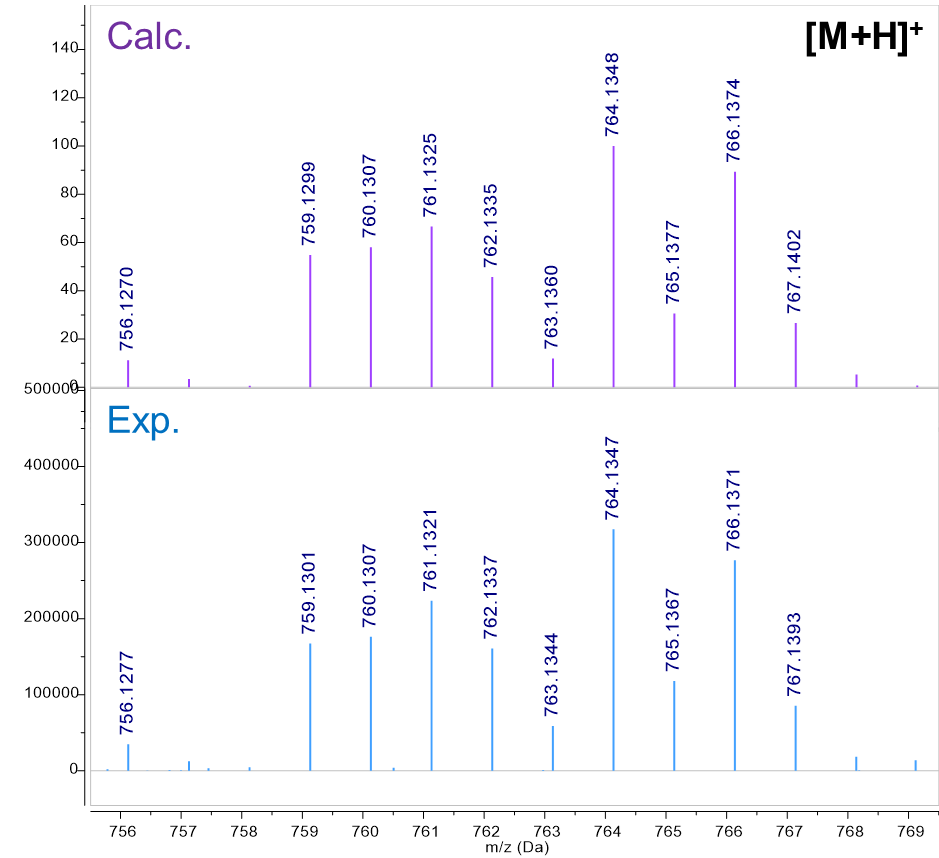


HRMS (ESI-QTOF) spectrum of **7-Sm**

Analytical HPLC chromatogram of **7-Eu**


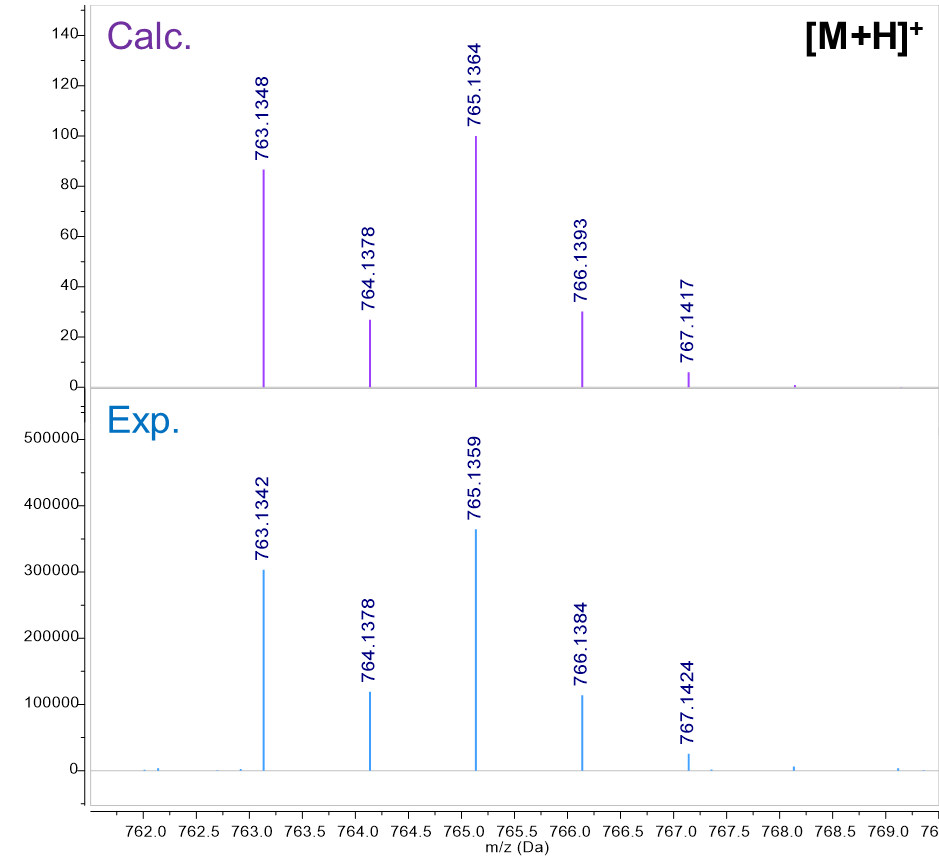


HRMS (ESI-QTOF) spectrum of **7-Eu**

Analytical HPLC chromatogram of **7-Tb**


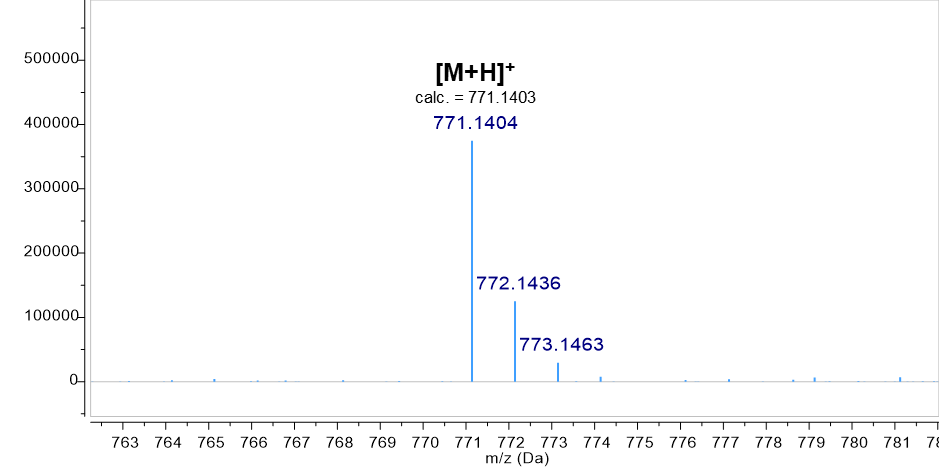


HRMS (ESI-QTOF) spectrum of **7-Tb**

Analytical HPLC chromatogram of **7-Yb**


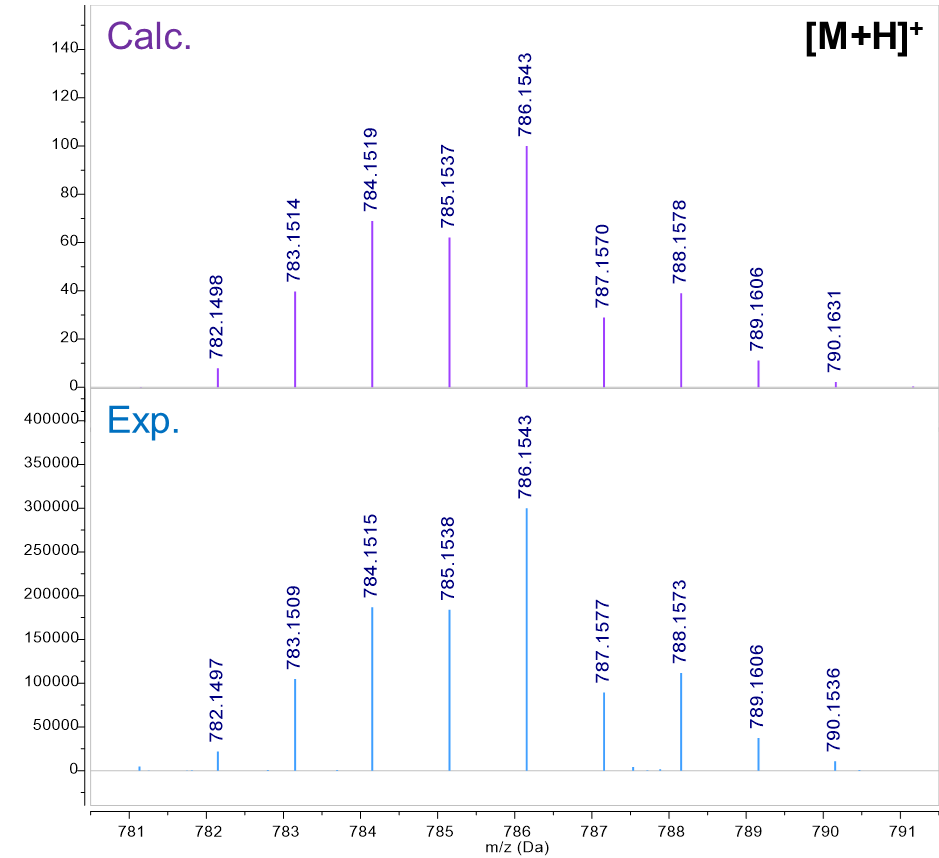


HRMS (ESI-QTOF) spectrum of **7-Yb**

Analytical HPLC chromatogram of **S8**


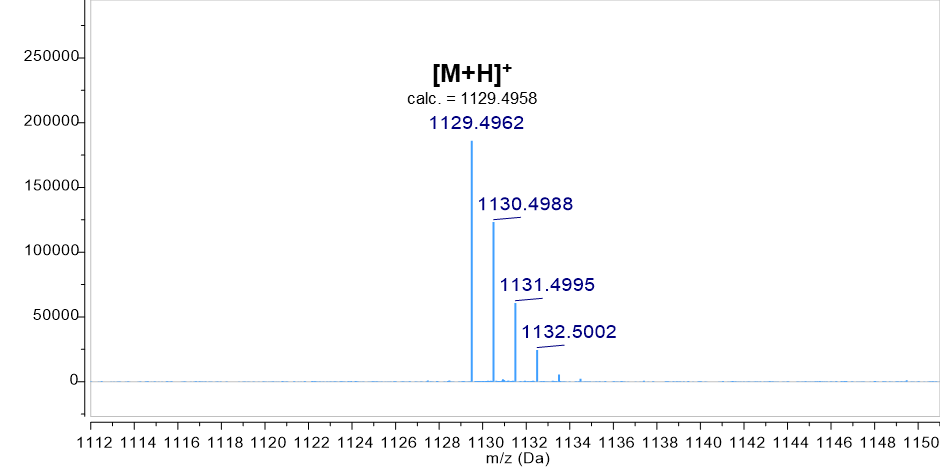


HRMS (ESI-QTOF) spectrum of **S8**

Analytical HPLC chromatogram of **S9**


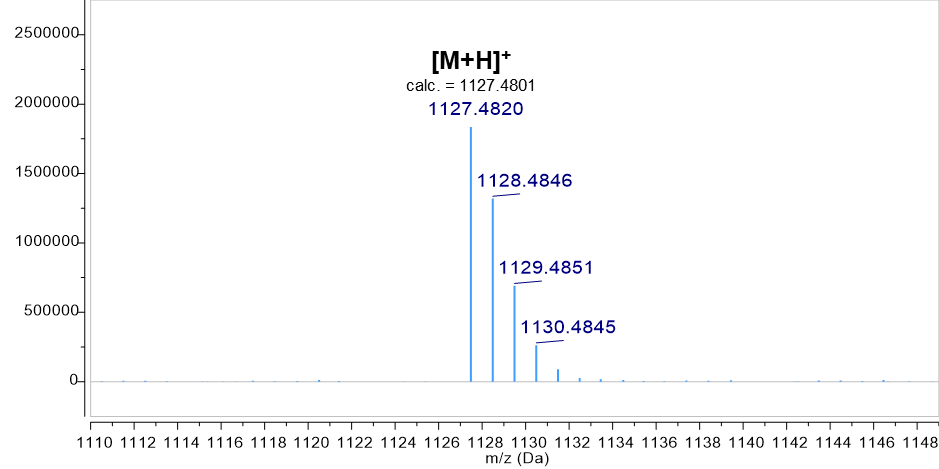


HRMS (ESI-QTOF) spectrum of **S9**

Analytical HPLC chromatogram of **8**


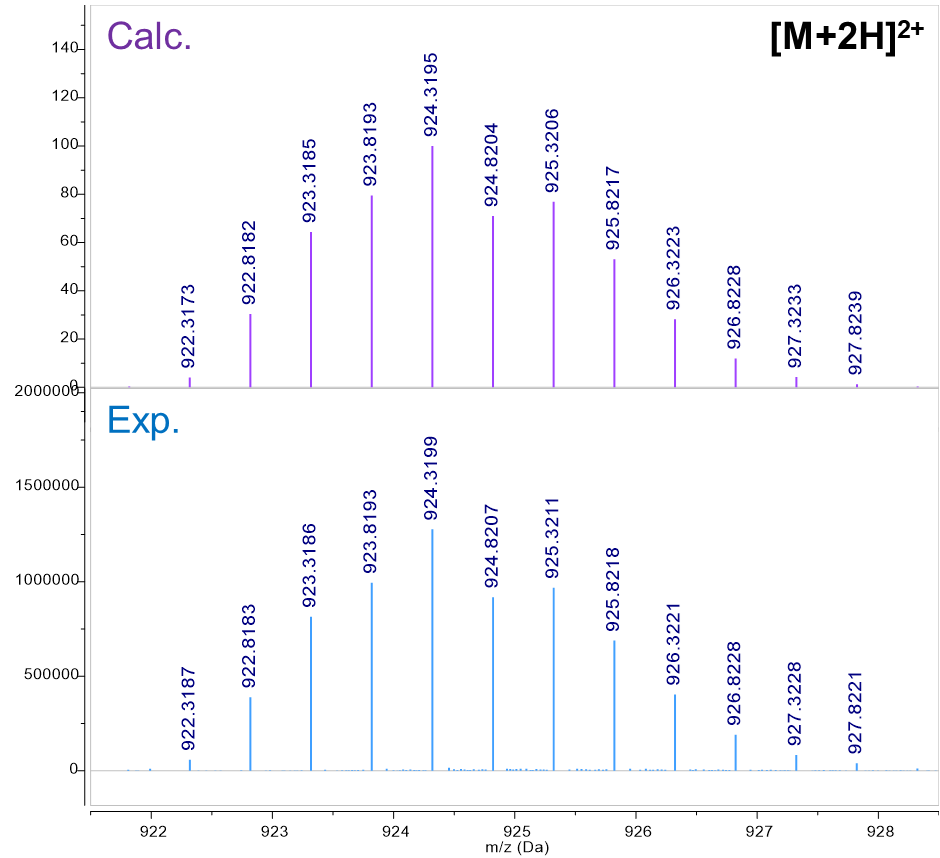


HRMS (ESI-QTOF) spectrum of **8**

Analytical HPLC chromatogram of **9**


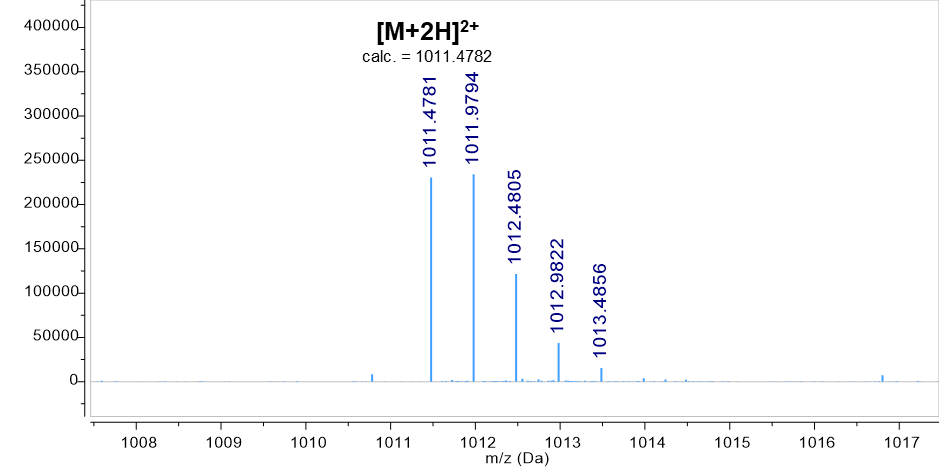


HRMS (ESI-QTOF) spectrum of **9**

Analytical HPLC chromatogram of **S11**


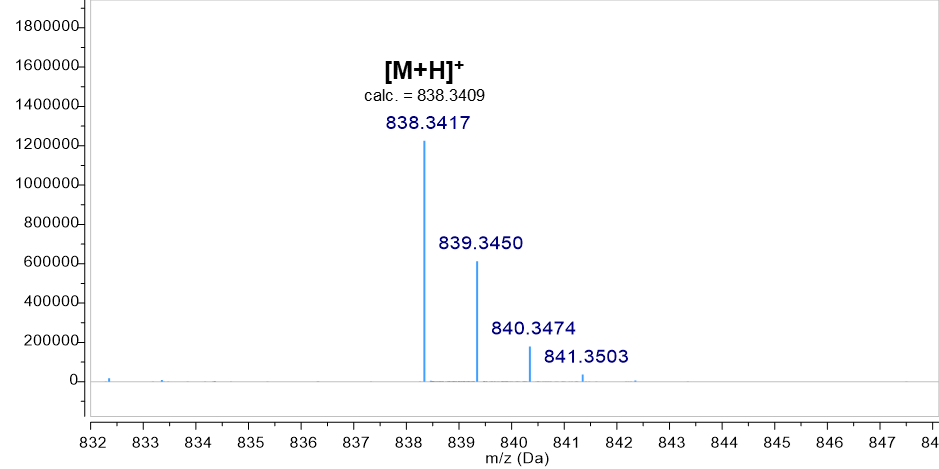


HRMS (ESI-QTOF) spectrum of **S11**

Analytical HPLC chromatogram of **10**


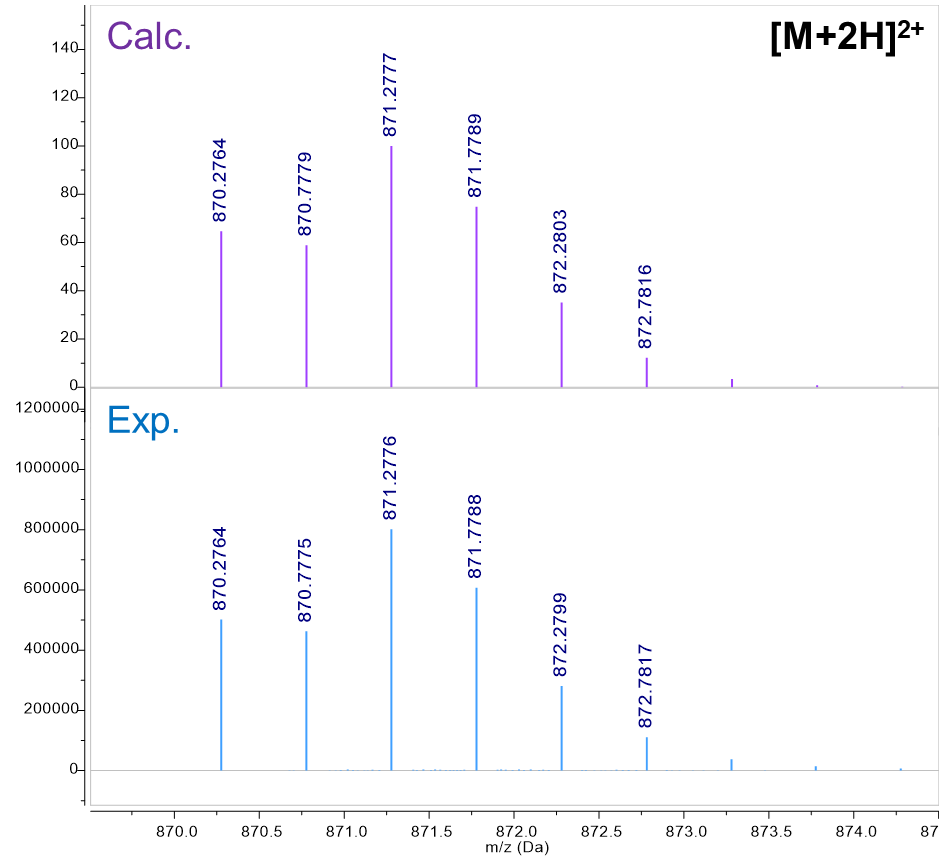


HRMS (ESI-QTOF) spectrum of **10**

Analytical HPLC chromatogram of **S12**


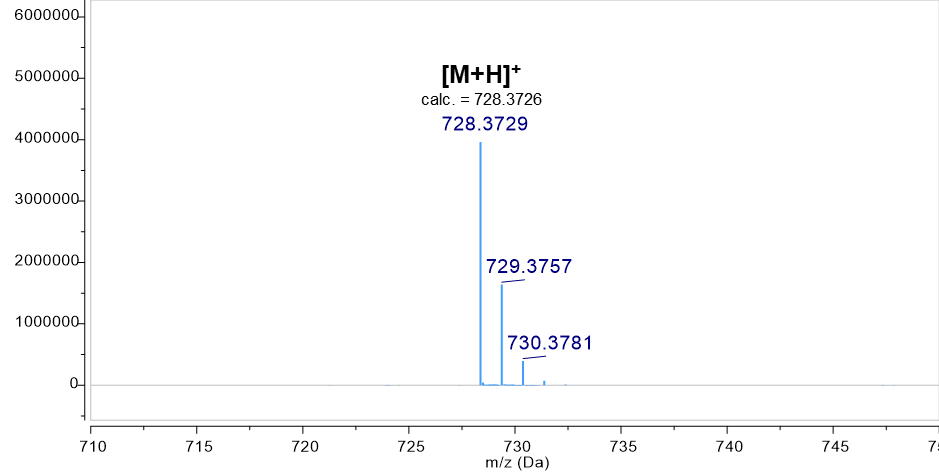


HRMS (ESI-QTOF) spectrum of **S12**

Analytical HPLC chromatogram of **11**


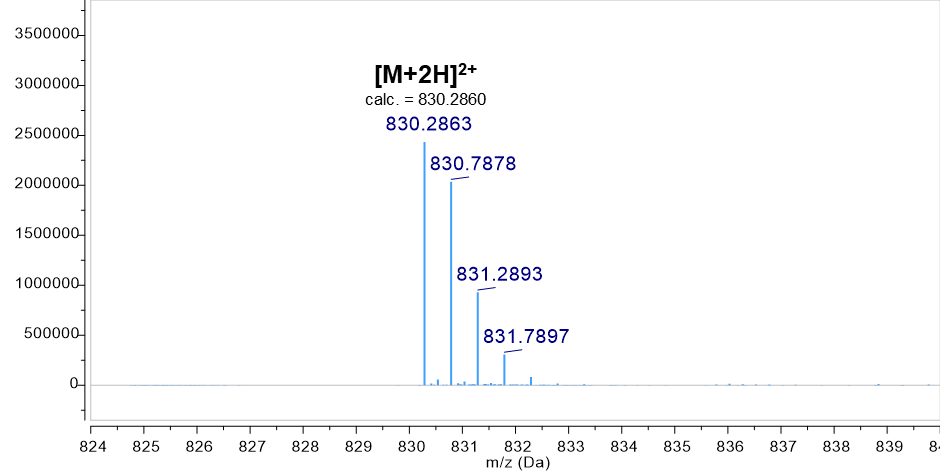


HRMS (ESI-QTOF) spectrum of **11**

Analytical HPLC chromatogram of **12**


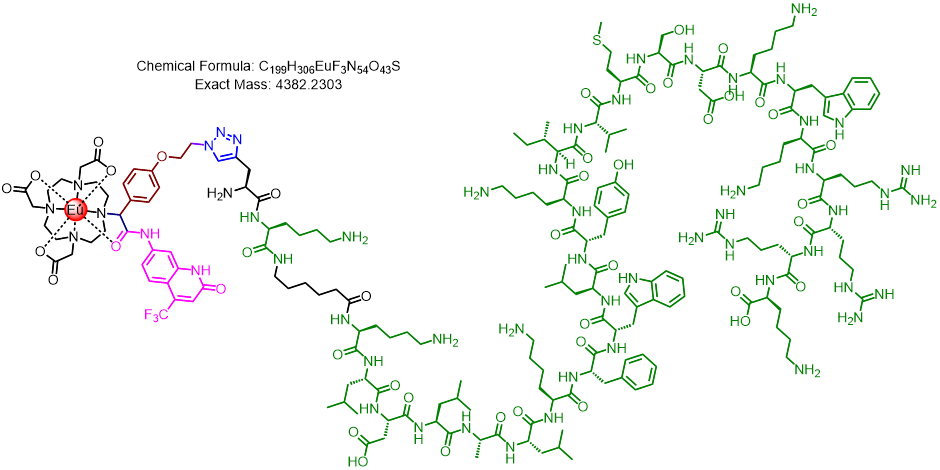


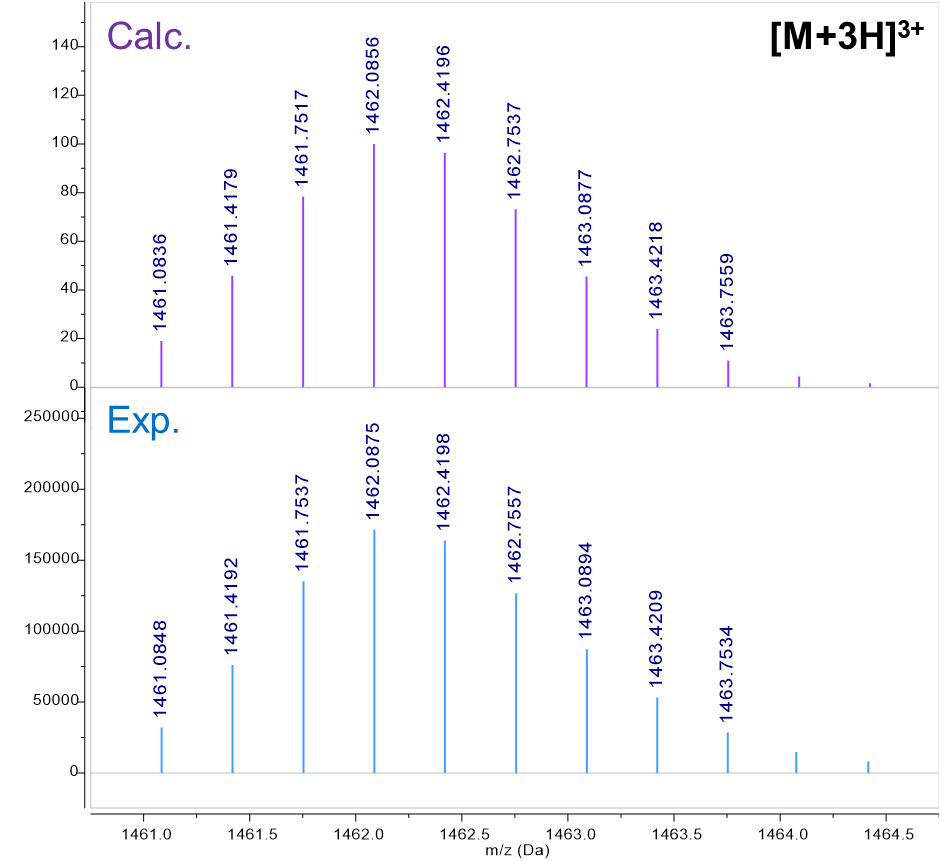


HRMS (ESI-QTOF) spectrum of **12**

Analytical HPLC chromatogram of **13**


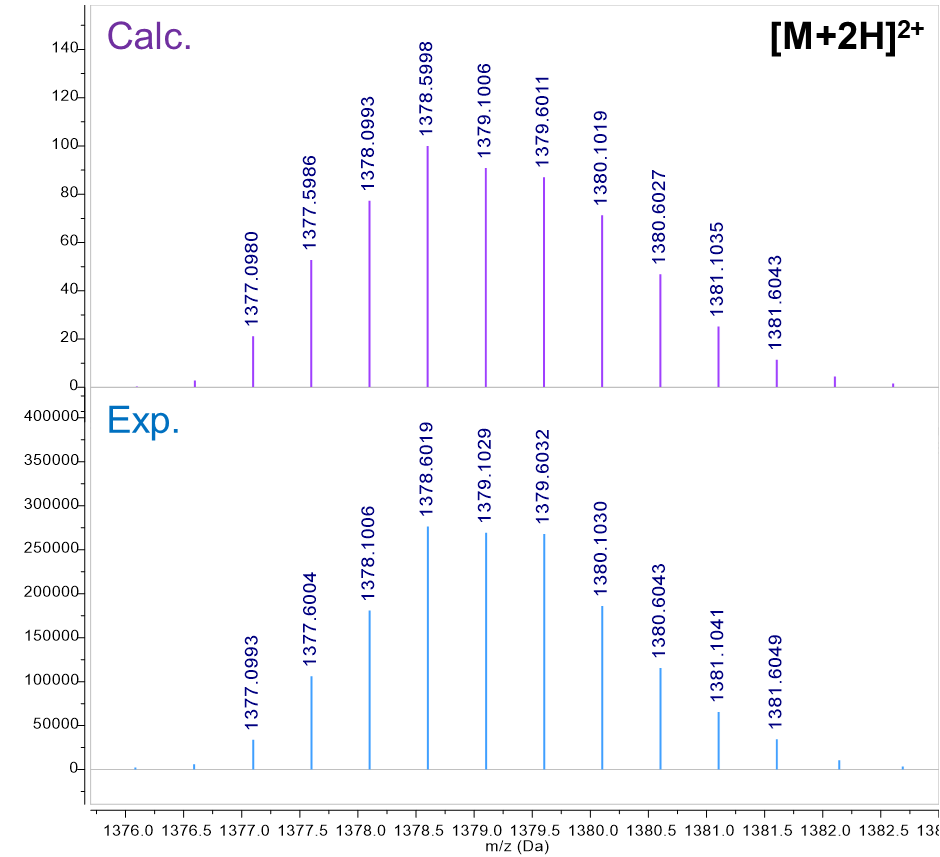


HRMS (ESI-QTOF) spectrum of **13**

# NMR spectra of products

^1^H NMR spectrum of Boc-Ser(*^t^*Bu)-CS124^CF3^ **S1** in DMSO-*d*_6_

^13^C NMR spectrum of Boc-Ser(*^t^*Bu)-CS124^CF3^ **S1** in DMSO-*d*_6_

^1^H NMR spectrum of H-Ser-CS124^CF3^ **S2** in DMSO-*d*_6_

^13^C NMR spectrum of H-Ser-CS124^CF3^ **S2** in DMSO-*d*_6_

^1^H NMR spectrum of Oxo-Ac-CS124^CF3^ **2b** (in hydrated form) in 5% D_2_O in MeOH-*d*_4_

^13^C NMR spectrum of Oxo-Ac-CS124^CF3^ **2b** (in hydrated form) in 5% D_2_O in MeOH-*d*_4_

^1^H NMR spectrum of *t*-butyl ester **S3** in DMSO-*d*_6_

^13^C NMR spectrum of *t*-butyl ester **S3** in DMSO-*d*_6_

^1^H NMR spectrum of acetic acid **S4** in DMSO-*d*_6_

^13^C NMR spectrum of acetic acid **S4** in DMSO-*d*_6_

^1^H NMR spectrum of methyl hexanoate **S5** in DMSO-*d*_6_

^13^C NMR spectrum of methyl hexanoate **S5** in DMSO-*d*_6_

^1^H NMR spectrum of hexanoic acid **S6** in DMSO-*d*_6_

^13^C NMR spectrum of hexanoic acid **S6** in DMSO-*d*_6_

^1^H NMR spectrum of alkyne-bearing phenylboronic acid **3c** in DMSO-*d*_6_

^13^C NMR spectrum of alkyne-bearing phenylboronic acid **3c** in DMSO-*d*_6_

^1^H NMR spectrum of tetrazine-bearing phenylboronic acid **3d** in DMSO-*d*_6_

^13^C NMR spectrum of tetrazine-bearing phenylboronic acid **3d** in DMSO-*d*_6_

^1^H NMR spectrum of DBCO-bearing phenylboronic acid **3e** in DMSO-*d*_6_

^13^C NMR spectrum of DBCO-bearing phenylboronic acid **3e** in DMSO-*d*_6_

^1^H NMR spectrum of maleimide-bearing phenylboronic acid **3f** in DMSO-*d*_6_

^13^C NMR spectrum of maleimide-bearing phenylboronic acid **3f** in DMSO-*d*_6_

^1^H NMR spectrum of phenylboronic acid of RGD conjugate **3h** in DMSO-*d*_6_

^13^C NMR spectrum of phenylboronic acid of RGD conjugate **3h** in DMSO-*d*_6_

^1^H NMR spectrum of phenylboronic acid of *c*(RGDyK) conjugate **3k** in DMSO-*d*_6_

^13^C NMR spectrum of phenylboronic acid of *c*(RGDyK) conjugate **3k** in DMSO-*d*_6_

^1^H NMR spectrum of α-aryl-substituted DOTA **4a** in DMSO-*d*_6_

^13^C NMR spectrum of α-aryl-substituted DOTA **4a** in DMSO-*d*_6_

^1^H NMR spectrum of α-aryl-substituted DOTA-azide building block **4b** in DMSO-*d*_6_

^13^C NMR spectrum of α-aryl-substituted DOTA-azide building block **4b** in DMSO-*d*_6_

^1^H NMR spectrum of α-aryl-substituted DOTA-alkyne building block **4c** in DMSO-*d*_6_

^13^C NMR spectrum of α-aryl-substituted DOTA-alkyne building block **4c** in DMSO-*d*_6_

^1^H NMR spectrum of α-aryl-substituted DOTA-tetrazine building block **4d** in DMSO-*d*_6_

^13^C NMR spectrum of α-aryl-substituted DOTA-tetrazine building block **4d** in DMSO-*d*_6_

^1^H NMR spectrum of α-aryl-substituted DOTA-DBCO building block **4e** in DMSO-*d*_6_

^13^C NMR spectrum of α-aryl-substituted DOTA-DBCO building block **4e** in DMSO-*d*_6_

^1^H NMR spectrum of α-aryl-substituted DOTA-Et-*N*(Boc) precursor **4f-NHBoc** in DMSO-*d*_6_

^13^C NMR spectrum of α-aryl-substituted DOTA-Et-*N*(Boc) precursor **4f-NHBoc** in DMSO-*d*_6_

^1^H NMR spectrum of α-aryl-substituted DOTA-Et-NH_2_ precursor **4f-NH_2_** in DMSO-*d*_6_

^13^C NMR spectrum of α-aryl-substituted DOTA-Et-NH_2_ precursor **4f-NH_2_** in DMSO-*d*_6_

^1^H NMR spectrum of α-aryl-substituted DOTA-maleimide building block **4f** in DMSO-*d*_6_

^13^C NMR spectrum of α-aryl-substituted DOTA-maleimide building block **4f** in DMSO-*d*_6_

^1^H NMR spectrum of α-aryl-substituted DOTA-*N*(Boc) precursor **4g-NHBoc** in DMSO-*d*_6_

^13^C NMR spectrum of α-aryl-substituted DOTA-*N*(Boc) precursor **4g-NHBoc** in DMSO-*d*_6_

^1^H NMR spectrum of α-aryl-substituted DOTA-isothiocyanate building block **4g** in DMSO-*d*_6_

^13^C NMR spectrum of α-aryl-substituted DOTA-isothiocyanate building block **4g** in DMSO-*d*_6_

^1^H NMR spectrum of α-aryl-substituted DOTA of RGD conjugate **4h** in DMSO-*d*_6_

^13^C NMR spectrum of α-aryl-substituted DOTA of RGD conjugate **4h** in DMSO-*d*_6_

^1^H NMR spectrum of α-aryl-substituted DOTA of PSMA-sequence conjugate **4i** in DMSO-*d*_6_

^13^C NMR spectrum of α-aryl-substituted DOTA of PSMA-sequence conjugate **4i** in DMSO-*d*_6_

^1^H NMR spectrum of α-aryl-substituted DOTA of *c*(RGDyK) conjugate **4k** in DMSO-*d*_6_

^13^C NMR spectrum of α-aryl-substituted DOTA of *c*(RGDyK) conjugate **4k** in DMSO-*d*_6_

^1^H NMR spectrum of DO3A-Gly-Tyr(Me)-CONH_2_ **5** in DMSO-*d*_6_

^13^C NMR spectrum of DO3A-Gly-Tyr(Me)-CONH_2_ **5** in DMSO-*d*_6_

^1^H NMR spectrum of luminescent α-aryl-substituted DO3A **6a** in DMSO-*d*_6_

^13^C NMR spectrum of luminescent α-aryl-substituted DO3A **6a** in DMSO-*d*_6_

^1^H NMR spectrum of luminescent α-aryl-substituted DO3A-azide **6b** in DMSO-*d*_6_

^13^C NMR spectrum of luminescent α-aryl-substituted DO3A-azide **6b** in DMSO-*d*_6_

^1^H NMR spectrum of alkyne-bearing Taxol **S11** in DMSO-*d*_6_

^13^C NMR spectrum of alkyne-bearing Taxol **S11** in DMSO-*d*_6_
